# Supplementary material for: Controllable DNA hybridization by host–guest complexation-mediated ligand invasion
Source: Nat Commun. 2022 Oct 8;13:5936. doi: 10.1038/s41467-022-33738-3 (PMC9547909; doi:10.1038/s41467-022-33738-3)
Supplement: Supplementary file 1 — Supplementary Information [file 41467_2022_33738_MOESM1_ESM.pdf]

Supplementary Information for

Controllable DNA Hybridization by Host-Guest Complexation-Mediated Ligand Invasion

Lin Xiao[1,†], Liang-Liang Wang[1,†], Chao-Qun Wu[1], Han Li[1], Qiu-Long Zhang[1], Yang Wang[1] and Liang Xu[1,*]

[1] MOE Key Laboratory of Bioinorganic and Synthetic Chemistry, School of Chemistry, Sun Yat-Sen University, Guangzhou, 510275, China.

* E-mail: xuliang33@mail.sysu.edu.cn

[†] These authors contribute equally to this work.

Table of Contents

Supplementary Methods 2

Synthesis 2

Supplementary Tables 16

Supplementary Table 1. 16

Supplementary Table 2. 17

Supplementary Figures 18

Supplementary Fig. 1. 18

Supplementary Fig. 2. 19

Supplementary Fig. 3. 20

Supplementary Fig. 4. 21

Supplementary Fig. 5. 22

Supplementary Fig. 6. 22

Supplementary Fig. 7. 23

Supplementary Fig. 8. 24

Supplementary Fig. 9. 25

Supplementary Fig. 10. 26

Supplementary Fig. 11. 26

Supplementary Fig. 12. 27

Supplementary Fig. 13. 27

Supplementary Fig. 14. 28

Supplementary Fig. 15. 29

Supplementary Fig. 16. 29

Supplementary Fig. 17. 30

Supplementary Fig. 18. 30

Supplementary Fig. 19. 31

Supplementary Fig. 20. 32

Supplementary Figures (NMR spectra of guest-containing phosphoramidites) 33

Supplementary Figures (ESI spectra of guest-containing ODNs) 45

Supplementary Figures (Uncropped gel images) 49

Supplementary References 51

Supplementary Methods

Synthesis

The abbreviations used here are listed as followings: Ad, adamantane; Fc, ferrocene; AD, 1-adamantanemethyl(amine); FC, ferrocenyl methyl(amine); BA, 1,4-benzenedimethan(amine); TB, 4-tert-butylbenzyl(amine); TBDMS, tert-butyldimethylsilyl; TBDMS-Cl, tertbutylchlorodimethylsilane; DMF, N, N- dimethylformamide; DMSO, dimethyl sulfoxide; HCl, hydrogen chloride; $NaHCO_3$, sodium hydrogen carbonate; NaCl, sodium chloride; Na_2SO_4, sodium sulfate; DMAP, 4-dimethylaminopyridine; DCM, dichloromethane; DIPEA, N, N- diisopropylethylamine; N_2 , nitrogen; TPS, 2,4,6-triisopropylbenzenesulfonyl chloride; TBAF, tetrabutylammonium fluoride; THF, tetrahydrofuran; DMTr-Cl, 4, 4'-dimethoxytritylchloride; Py, pyridine; $(OCH_2CH_2CN)(i\text{Pr}_2N)PCl$, 2-cyanoethoxy-N, N-diisopropylaminochlorophosphine; BOP, 1H-benzotriazol-1-yloxytris(dimethylamino)phosphonium hexafluorophosphate; Cs_2CO_3, cesium carbonate; K_2CO_3, potassium carbonate; DME, 1,2-dimethoxyethane; NEt_3, triethylamine; r.t, room temperature.

Path A:

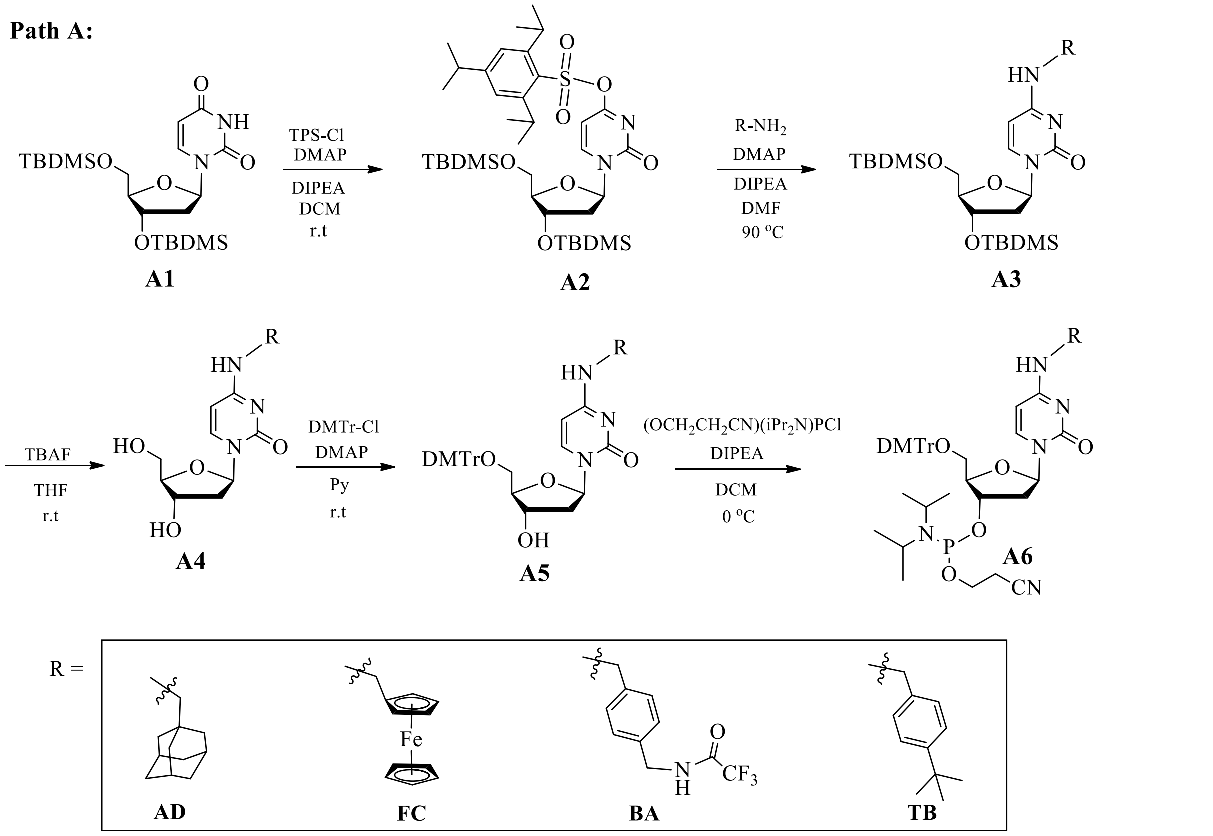

For synthesis of guest-modified cytidine (Path A), the TBDMS-protected uridine was first conjugated with the triisopropylbenzenesulfonyl activation group at the O^4 position, which was then followed by reactions with nucleophilic amine compounds to obtain the guest-modified cytidine

3'-5'-*O*-Bis-(tertbutyldimethylsilyl)-2'-deoxyuridine (A1)

2'-Deoxyuridine (6.9 g, 30.2 mmol), imidazole (10.3 g, 151 mmol, 5 eq.) and tertbutylchlorodimethylsilane (13.7 g, 90.7 mmol, 3 eq.) were placed in a 100 mL round bottom flask. The compounds were dissolved in dry DMF (50 mL) and the resulting solution was stirred at 50 °C overnight. After cooling to room temperature, the reaction mixture was treated with ethanol (20 mL) and stirred for additional 15 minutes. Then the solvent was evaporated; the residue was dissolved in ethyl acetate and washed consecutively with aq. HCl (1 M), sat. aq. $NaHCO_3$ and sat. aq. NaCl, then dried with Na_2SO_4. After evaporation of the solvent, the product was purified via flash chromatography with petroleum ether/ethyl acetate 9: 1 → 7:3, to give a white foam. Yield: 11.20 g (81 %), TLC (petroleum ether/ethyl acetate 1:1): Rf = 0.30. ^{1}H NMR (400 MHz, CDCl3-d_3) δ 8.53 (s, 1H, N-*H*), 7.91 (d, *J* = 8.2 Hz, *H*5), 6.28 (t, *J* = 6.2 Hz, 1H, 1'-*H*), 5.68 (d, *J* = 8.1 Hz, 1H, *H*6), 4.41 (m, 1H, 3'-*H*), 3.93–3.88 (m, 2H, 4'-H, 5'-*H*), 3.78–3.74 (m, 1H, 5'-*H*), 2.35–2.29 (m, 1H, 2'-*H*), 2.09–2.03 (m, 1H, 2'-*H*), 0.91 (s, 9H, SiC(CH_3)$_3$), 0.88 (s, 9H, SiC(CH_3)$_3$), 0.10 (s, 6H, 2x SiCH_3), 0.07 (s, 6H, 2x SiCH_3) ppm. ^{13}C NMR (101 MHz, $CDCl_3$-d_3) δ 164.0, 150.6, 140.3, 102.3(*C*=O, *C*=*C*), 87.8, 85.3, 71. 2, 62.4, 42.0 (sugar), 26.0, 25.8, 18.4, 18.1, - 4.5, -4.8, -5.4, -5.5 (Si(CH_3)$_2$(*C*(*C*H_3)$_3$)) ppm. MS(ESI) calcd for $C_{21}H_{40}N_2O_5Si_2$ 456.25, found $[M + H]^+$ 457.70.

3'-5'-*O*-Bis-(tertbutyldimethylsilyl)-*O*^{4}-(2,4,6-triisopropylbenzenesulfonyl)-2'-deoxyuridine (A2)

Compound A1 (10.0 g, 21.95 mmol) was dissolved in dry dichloromethane (135 mL) in a 250 mL round bottom flask. N, N-dimethylaminopyridine (267.9 mg, 2.19 mmol, 0.1 eq.) and N, N- diisopropylethylamine (15.3 mL, 87.8 mmol, 4 eq.) were added into the mixture. The solution was cooled in an ice bath to 0 °C for 10 min, followed by addition of 2,4,6-triisopropylbenzenesulfonylchloride (13.3, 43.90 mmol, 2.0 eq.). The solution was stirred for 20 minutes at 0 °C and then for additional 5 hours at room temperature. The reaction mixture was diluted with dichloromethane, washed with sat. aq. $NaHCO_3$, and then dried with Na_2SO_4. After evaporation of the solvent, the product was purified via flash chromatography with petroleum ether/ethyl acetate 97:3→ 92:6 to give a white foam. Yield: 10.28 g (65 %) TLC (petroleum ether/ethyl acetate 9:1): Rf = 0.51. ^{1}H NMR (400 MHz, $CDCl_3$-d_3): δ = 8.48 (d, *J* = 7.3 Hz, 1H, *H*5), 7.20 (s, 2 H, Ar-*H*), 6.08 (t, *J* = 6.5 Hz, 1H, 1'-*H*), 6.01 (d, *J* = 7.3 Hz, 1H, *H*6), 4.32 (m, 1H, 3'-*H*), 4.26 (hept, *J* = 6.9 Hz, 2 H, 2x *i*-Pr-C*H*), 3.95 (m, 1H, 4'-*H*), 3.93 (m, 1H, 5'-*H*), 3.75 (m, 1H, 5'-*H*), 2.91 (hept, *J* = 6.9 Hz, 1 H, *i*-Pr-C*H*), 2.54–2.45 (m, 1H, 2'-*H*), 2.16–2.10 (m, 1H, 2'-*H*), 1.35–1.22 (m, 18 H, *i*-Pr-CH_3), 0.90 (s, 9H, SiC(CH_3)$_3$), 0.86 (s, 9H, SiC(CH_3)$_3$), 0.10 (s, 6H, 2x SiCH_3), 0.04 (s, 6H, 2x SiCH_3) ppm. ^{13}C NMR (101 MHz, $CDCl_3$-d_3) δ 167.1, 154.6, 154.0, 151.3, 146.2, 130.8, 124.2, 94.6, 87.9, 87.4, 77.2, 69.6, 61.6, 42.2, 34.4, 29.8, 27.0, 26.0, 25.8, 24.8, 24.5, 23.6, 18.5, 18.0, -4.4, -4.8, -5.4 ppm. MS(ESI) calcd

for $C_{36}H_{62}N_2O_7SSi_2$ 722.38, found $[M + H]^+$ 723.53.

3'-5'-*O*-Bis-(tertbutyldimethylsilyl)-*N⁴*-(1-methyladamantane)-2'-deoxycytosine (A3^{AD})

Compound A2 (1.59 g, 2.20 mmol) and 1-aminomethyladamantane (701.5 µL, 3.95 mmol, 1.8 eq.), were dissolved in 30 ml dry DMF. N, N- diisopropylethylamine (1.15 mL 6.60 mmol, 3 eq.) were added and the reaction mixture was stirred at 90 °C overnight. The solvent was removed and the residue was dissolved in dichloromethane. After evaporation of the solvent, the product was purified via flash chromatography with petroleum ether/ethyl acetate 9:1→ 7:3 to give a white foam. Yield: 1.06 g (80 %) TLC (petroleum ether/ethyl acetate 1:1): Rf = 0.45. ^{1}H NMR (400 MHz, $CDCl_3$-d_3) δ 7.87 (d, *J* = 7.4 Hz, 1 H, *H*5), 6.30 (t, 1H, 1'-*H*), 5.54 (d, *J* = 7.7 Hz, 1 H, *H*6), 4.36 (br m, *J* = 5.7 Hz, 1H, 3'-*H*), 3.90 (m, 1H, 5'-*H*), 3.84 (m, 1H, 4'- *H*), 3.78 (m, 1H, 5'- *H*), 3.27–3.21 (m, 2H, N-CH_2), 2.84 (s, 1H, N-*H*), 2.39 (m, 1H, 2'-*H*), 2.08 (m, 1H, 2'-*H*), 1.98 (s, 3H, Ad-C*H*), 1.77–1.67 (m, 12H, Ad-CH_2), 0.95–0.84 (m, 18H, SiC(CH_3)$_3$), 0.10 (s, 6H, 2x SiCH_3), 0.04 (s, 6H, 2x SiCH_3) ppm. ^{13}C NMR (101 MHz, $CDCl_3$-d_3) δ 164.3, 155.5, 140.0, 94.6, 87.2, 85.8, 70.3, 62.1, 52.1, 42.3, 40.3, 37.1, 33.8, 28.4, 26.1, 25.9, 18.5, 18.1, -4.4, -4.8, -5.4, -5.4 ppm. MS(ESI) calcd for $C_{32}H_{57}N_3O_4Si_2$ 603.39, found $[M + H]^+$ 604.46.

3'-5'-*O*-Bis-(tertbutyldimethylsilyl)-*N⁴*-(1-methylferrocene)-2'-deoxycytosine (A3^{FC})

Following the same procedure as synthesis of A3^{AD}, ferrocenyl methylamine (813.7 mg, 3.95mmol, 1.8 eq.) reacted with Compound (A2) (1.59 g, 2.20 mmol) and the product was purified via flash chromatography with petroleum ether/ethyl acetate 9:1→ 7:3 to give a light-yellow foam. Yield: 1.00 g (70 %) TLC (petroleum ether/ethyl acetate 1:1): Rf = 0.50. ^{1}H NMR (400 MHz, $CDCl_3$-d_3) δ 7.89 (d, *J* = 7.2 Hz, 1 H, *H*5), 6.29 (t, *J* = 5.8 Hz, 1H, 1'-*H*), 5.47 (d, J = 7.3 Hz, 1 H, *H*6), 5.05 (m, 1H, N-*H*) ,4.36 (m, 3H, 3'- *H*, N-CH_2), 4.22–4.11 (m, 9H, Fc-*H*), 3.95–3.83 (m, 2H, 5'- *H*, 4'- *H*), 3.75 (m, 1H, 5'- *H*), 2.41 (dt, *J* = 12.8, 6.1 Hz, 1H, 2'-*H*), 2.08 (dt, *J* = 12.5, 5.8 Hz, 1H, 2'-*H*), 0.89 (s, 18H, SiC(CH_3)$_3$), 0.08 (s, 6H, 2x SiCH_3), 0.04 (s, 6H, 2x SiCH_3) ppm. ^{13}C NMR (101 MHz, $CDCl_3$-d_3) δ 164.3, 155.5, 140.0, 94.6, 87.2, 85.8, 70.3, 62.1, 52.1, 42.3, 40.3, 37.1, 28.4, 26.1, 25.9, 18.5, 18.1, -4.4, -4.8, -5.4, -5.4 ppm. MS calcd for $C_{32}H_{51}FeN_3O_4Si_2$ 653.28, found $[M + H]^+$ 654.36.

3'-5'-*O*-Bis-(tertbutyldimethylsilyl)-*N⁴*-(4-(aminomethyl)benzyl)-trifluoroacetamide)-2'-deoxycytosine (A3^{BA})

Following the same procedure as synthesis of A3^{AD}, N-(4-(aminomethyl)benzyl)-trifluoroacetamide (916.4mg, 3.95 mmol, 1.8 eq.) reacted with Compound (A2) (1.59 g, 2.20 mmol) and the product was purified via flash chromatography with petroleum ether/ethyl acetate 9:1→ 5:5 to give a white foam. Yield: 0.88 g (60 %) TLC (petroleum ether/ethyl acetate 1:1):

Rf = 0.20. ^{1}H NMR (400 MHz, $CDCl_3$-d_3) δ 8.03 (s, 1H, N-*H*), 7.87 (d, *J* = 7.4 Hz, 1H, *H*5), 7.14 (s, 4H, Ar-*H*), 6.21 (t, *J* = 5.8 Hz, 1H, 1'-*H*), 5.97 (s, 1H, N-*H*), 5.65 (d, *J* = 7.4 Hz, 1H, *H*6), 4.41 (m, 5H, 3'-*H*, N-CH_2), 3.87 (m, 2H, 5'-*H*, 4'-*H*), 3.75 (d, J = 10.9 Hz, 1H, 5'-*H*), 2.36 (dt, *J* = 12.9, 6.0 Hz, 1H, 2'-*H*), 2.03 (dt, *J* = 13.1, 6.0 Hz, 1H, 2'-*H*), 0.09 (s, 18H, SiC(CH_3)$_3$), 0.08 (s, 6H, 2x SiCH_3), 0.05 (s, 6H, 2x SiCH_3) ppm. ^{13}C NMR (101 MHz, $CDCl_3$-d_3) δ 163.6, 162.7, 157.8, 157.4, 156.5, 140.1, 137.9, 135.8, 117.6, 114.7, 95.0, 87.4, 85.9, 77.5, 77.2, 76.8, 70.5, 62.1, 44.3, 43.4, 42.2, 36.6, 31.6, 26.0, 25.8, 18.5, 18.1, -4.4, -4.8, -5.4, -5.4 ppm. MS(ESI) calcd for $C_{31}H_{49}F_3N_4O_5Si_2$ 670.32, found $[M + H]^+$ 671.56.

3'-5'-*O*-Bis-(tertbutyldimethylsilyl)-*N*^{4}-(methyl(4-(tert-butyl)phenyl))-2'-deoxycytosine (A3^{TB})

Following the same procedure as synthesis of A3^{AD}, (4-(tert-butyl)phenyl)methanamine (692.3 µL, 3.95 mmol, 1.8 eq.) reacted with Compound (A2) (1.59 g, 2.20 mmol) and the product was purified via flash chromatography with petroleum ether/ethyl acetate 9:1→ 7:3 to give a white foam. Yield: 1.03 g (78 %) TLC (petroleum ether/ethyl acetate 1:1): Rf = 0.45. ^{1}H NMR (400 MHz, $CDCl_3$-d_3) δ 7.95 (d, *J* = 7.4 Hz, 1H, *H*5), 7.36 (dd, *J* = 8.3 Hz, 2H, Ar-*H*), 7.25 (dd, *J* = 7.2 Hz, 2H, Ar-*H*), 6.29 (t, *J* = 5.8 Hz, 1H, 1'-*H*), 5.52 (d, *J* = 7.4 Hz, 1H, *H*6), 5.11 (m, 1H, N-*H*), 4.64 (m, 2H, N-CH_2), 4.36 (m, 1H, 3'-*H*), 3.95–3.84 (m, 2H, 5'- *H*, 4'- *H*), 3.76 (d, *J* = 11.3 Hz, 1H, 5'- *H*), 2.42 (dt, *J* = 13.1, 7.1, 5.3 Hz, 1H, 2'-*H*), 2.10 (dt, *J* = 12.9, 5.8 Hz, 1H, 2'-*H*), 1.31 (s, 9H, C(CH_3)$_3$), 0.89 (s, 18H, SiC(CH_3)$_3$), 0.11–0.03 (s, 12H, 2x SiCH_3) ppm. ^{13}C NMR (101 MHz, $CDCl_3$-d_3) δ 150.9, 140.4, 135.0, 128.2, 127.8, 125.8, 94.4, 87.3, 86.0, 77.2, 70.2, 62.0, 42.3, 34.7, 31.5, 26.0, 25.9, 18.5, 18.1, -4.4, -4.8, -5.4, -5.4 ppm. MS(ESI) calcd for $C_{32}H_{55}N_3O_4Si_2$ 601.37, found $[M + H]^+$ 602.46.

***N*^{4}-(1-methyladamantane)-2'-deoxycytosine (A4^{AD})**

Compound A3^{AD} (800 mg, 1.33 mmol) was dissolved in 15 mL dry THF. 2.78 mL (2.65 mmol, 2 eq.) TBAF solution (1M in THF) was added and the solution was stirred for 3 hours at room temperature. The solvent was removed under reduced pressure. The residue was dissolved in dichloromethane and purified by flash chromatography (dichloromethane/ methanol 97:3→95:5→9:1) to give a white solid. Yield: 442.7 mg (89 %) TLC (CH_2Cl_2/ methanol 9:1): Rf = 0.28. ^{1}H NMR (400 MHz, DMSO-d_6) δ 7.70 (d, *J* = 7.5 Hz, 1H, *H*5), 7.52 (d, *J* = 6.3 Hz, 1H, N-*H*), 6.16 (t, *J* = 6.8 Hz, 1H, 1'-*H*), 5.87 (d, *J* = 7.5 Hz, 1H, *H*6), 5.18 (m, *J* = 4.1 Hz, 1H, 3'-O*H*), 4.95 (d, *J* = 5.6 Hz, 1H, 5'-O*H*), 4.20 (m, 1H, 3'-*H*), 3.78 –3.72 (m, 1H, 4'-*H*), 3.54 (q, J = 4.9 Hz, 2H, 5'-*H*), 3.02 (d, J = 6.0 Hz, 2H, N-CH_2), 2.14–2.03 (m, 1H, 2'-*H*), 1.94 (m, 1H, 2'-*H*), 1.94 (m, 3H, Ad-C*H*), 1.69–1.54 (m, 6H, Ad-CH_2), 1.47 (s, 6H, Ad-CH_2) ppm. ^{13}C NMR (101 MHz, DMSO-d_6) δ 164.1, 155.2, 139.5, 94.9, 87.1, 84.8, 70.5, 61.5, 51.0, 45.7, 40.2, 39.8, 36.5, 33.8, 27.7 ppm. MS(ESI) calcd for $C_{20}H_{29}N_3O_4$ 375.22, found $[M + H]^+$ 376.41.

***N*^{4}-(1-methylferrocene)-2'-deoxycytosine (A4^{FC})**

Following the same procedure as synthesis of A4^{AD}, A3^{FC} (800 mg, 1.23 mmol) reacted with TBAF to obtain the product. Yield: 469.6 g (90 %) TLC (CH_2Cl_2/ methanol 9:1): Rf = 0.30. ^{1}H NMR (400 MHz, DMSO-d_6) δ 7.94 (d, *J* = 5.8 Hz, 1H, N-*H*), 7.76 (d, *J* = 7.3 Hz, 1H, *H*5), 6.18 (t, J = 6.8 Hz, 1H, 1'-*H*), 5.83 (d, *J* = 7.4 Hz, 1H, *H*6), 5.27 (m, 1H, 3'-O*H*), 4.26 –4.04 (m, 12H, Fc-*H*, N-CH_2, 5'-O*H,* 3'-*H*), 3.76 (d, *J* = 4.1 Hz, 1*H,* 4'-*H*), 3.54 (q, J = 10.1, 8.1 Hz, 2H, 5'-*H*), 2.10 (dt, J = 13.1, 6.1, 3.2 Hz, 1H, 2'-*H*), 1.93 (dt, J = 13.3, 6.6 Hz, 1H, 2'-*H*) ppm. ^{13}C NMR (101 MHz, DMSO-d_6) δ 162.8, 155.2, 139.9, 94.7, 87.2, 85.2, 84.8, 70.4, 68.4, 68.4, 67.5, 67.0, 45.7, 40.3 ppm. MS(ESI) calcd for $C_{20}H_{23}FeN_3O_4$ 425.10, found $[M + H]^+$ 426.54.

***N*^{4}-(4-(aminomethyl)benzyl)-trifluoroacetamide)-2'-deoxycytosine (A4^{BA})**

Following the same procedure as synthesis of A4^{AD}, A3^{BA} (800 mg, 1.19 mmol) reacted with TBAF to obtain the product. Yield: 438.0 mg (83 %) TLC (CH_2Cl_2/ methanol 9:1): Rf = 0.20. ^{1}H NMR (400 MHz, DMSO-d_6) δ 10.00 (t, *J* = 6.0 Hz, 1H, N-*H*), 8.14 (t, *J* = 5.8 Hz, 1H, N-*H*), 7.78 (d, *J* = 7.5 Hz, 1H, *H*5), 7.31–7.20 (m, 4H, Ar-*H*), 6.15 (dd, *J* = 7.4, 6.0 Hz, 1H, 1'-*H*), 5.81 (d, 1H, *H*6), 5.20 (d, *J* = 4.2 Hz, 1H, 3'-O*H*), 4.97 (t, *J* = 5.3 Hz, 1H, 5'-O*H*), 4.46 (d, *J* = 5.8 Hz, 2H, N-CH_2), 4.36 (d, *J* = 6.0 Hz, 2H, N-CH_2), 4.19 (dq, *J* = 6.8, 3.4 Hz, 1H, 3'-*H*), 3.76 (q, *J* = 3.7 Hz, 1H, 4'-*H*), 3.54 (m, 2H, 5'-*H*), 2.10 (ddd, *J* = 13.1, 6.0, 3.2 Hz, 1H, 2'-*H*), 1.93 (ddd, *J* = 13.3, 7.6, 6.0 Hz, 1H, 2'-*H*) ppm. ^{13}C NMR (101 MHz, DMSO-d_6) δ 163.3, 156.5, 156.1, 155.0, 140.2, 138.2, 136.2, 127.6, 127.5, 117.5, 114.6, 94.5, 87.2, 84.9, 70.4, 61.4, 54.9, 42.9, 42.4, 40.3, 40.1, 39.9, 39.7, 39.5, 39.3, 39.1, 38.9 ppm. MS(ESI) calcd for $C_{19}H_{21}F_3N_4O_5$ 442.15, found $[M + H]^+$ 443.20.

***N*^{4}-(methyl(4-(tert-butyl)phenyl))-2'-deoxycytosine (A4^{TB})**

Following the same procedure as synthesis of A4^{AD}, A3^{TB} (800 mg, 1.33 mmol)) reacted with TBAF to obtain the product. Yield: 456.8 mg (92 %) TLC (CH_2Cl_2/ methanol 9:1): Rf = 0.28. ^{1}H NMR (400 MHz, DMSO-d_6) δ 8.12 (dt, *J* = 8.1, 4.3 Hz, 1H, N-*H*), 7.78 (dd, *J* = 7.4, 2.4 Hz, 1H, *H*5), 7.35 (d, *J* = 7.9 Hz, 2H, Ar-H), 7.22 (d, *J* = 7.9 Hz, 2H, Ar-H), 6.16 (t, *J* = 6.8 Hz, 1H, 1'-*H*), 5.82 (dd, *J* = 7.5, 2.5 Hz, , 1H, *H*6), 5.23 (m, 1H, 3'-O*H*), 5.01 (m, 1H, 5'-O*H*), 4.43 (d, *J* = 5.6 Hz, 2H, N-CH_2), 4.20 (d, *J* = 5.5 Hz, 1H, 3'-*H*), 3.76 (q, *J* = 3.3 Hz, 1H , 4'-*H*), 3.58 (s, , 2H, 5'-*H*), 2.10 (ddd, *J* = 12.4, 5.9, 3.0 Hz, 1H, 2'-*H*), 1.94 (dt, *J* = 13.4, 6.6 Hz, 1H, 2'-*H*), 1.26 (s, 9H, C(CH_3)$_3$) ppm. ^{13}C NMR (101 MHz, DMSO-d_6) δ 163.2, 155.0, 149.3, 140.0, 135.9, 127.3, 125.1, 94.5, 87.2, 84.8, 70.4, 61.4, 45.7, 42.9, 40.3, 40.1, 34.2, 31.1 ppm.

MS(ESI) calcd for $C_{20}H_{27}N_3O_4$ 373.20, found $[M + H]^+$ 374.23.

5'-*O*-(4,4'-Dimethoxytrityl)-*N*^{4}-(1-methyladamantane)-2'-deoxycytosine (A5^{AD})

Compound A4^{AD} (400 mg, 1.06 mmol) was dissolved in 15 mL dry pyridine. 4-dimethylaminopyridine(12.2 mg, 0.10 mmol, 0.1 eq.) and 4, 4'-dimethoxytritylchloride (542.1 mg, 1.60 mmol, 1.5 eq.) were added and the reaction mixture was stirred overnight at room temperature. The reaction solution was added to ten times the volume of petroleum ether, centrifuged at high speed, and the precipitate was dissolved in dichloromethane and purified by flash chromatography (dichloromethane/ methanol 100:0→99:1→99:3). A colourless foam was obtained. Yield: 433.3 mg (60%) TLC (CH_2Cl_2/ methanol 9:1): Rf = 0.60. ^{1}H NMR (400 MHz, DMSO-d_6) δ 7.56 (dd, J = 6.5 Hz, 1H, *H*5), δ 7.56 (m 1H, N-*H*) 7.43– 7.19 (m, 9H, Ar-*H*), 6.94–6.85 (m, 4H, Ar-*H*), 6.19 (t, J = 6.5 Hz, 1H, 1'-*H*), 5.75 (dd, J = 6.8, 2.0 Hz, 1H, *H*6), 5.32 (d, J = 7.2 Hz, 1H, 3'-O*H*), 4.25 (t, J = 5.0 Hz, 1H, 3'-*H*), 3.87 (q, J = 4.1 Hz, 1H, 4'-*H*), 3.74 (s, 6H, OCH_3), 3.26–3.14 (m, 2H, 5'-*H*), 3.09–2.95 (m, 2H, N-CH_2), 2.17 (ddd, J = 13.3, 6.4, 4.4 Hz, 1H, 2'-*H*), 2.09–1.96 (m, 1H, 2'-*H*), 1.96-1.89 (m, 3H , Ad-C*H*), 1.70–1.54 (m, 6H, Ad-CH_2), 1.48 (d, J = 2.8 Hz, 6H, Ad-CH_2) ppm. ^{13}C NMR (101 MHz, DMSO-d_6) δ 164.0, 158.1, 155.0, 144.8, 139.2, 135.5, 135.3, 129.7, 127.9, 127.7, 126.7, 113.2, 94.8, 85.7, 85.1, 84.5, 70.1, 63.5, 59.8, 55.0, 51.1, 45.7, 40.4, 36.5, 33.8, 27.7, 11.4 ppm. MS(ESI) calcd for $C_{41}H_{47}N_3O_6$ 677.35, found $[M + H]^+$ 678.42.

5'-*O*-(4,4'-Dimethoxytrityl)-*N*^{4}-(1-methylferrocene)-2'-deoxycytosine (A5^{FC})

Following the same procedure as synthesis of A5^{AD}, A4^{FC} (450.5 mg, 1.06 mmol) reacted with 4, 4'-dimethoxytritylchloride to obtain the product. Yield: 423.8 g (55 %) TLC (CH_2Cl_2/ methanol 9:1): Rf = 0.65. ^{1}H NMR (400 MHz, DMSO-d_6) δ 7.80 (d, J = 6.1 Hz, 1H, N-*H*), 7.61 (d, J = 7.5 Hz, 1H, *H*5), 7.38–7.06 (m, 9H, Ar-*H*), 6.92–6.77 (m, 4H, Ar-*H*), 6.20 (t, J = 6.5 Hz, 1H, 1'-*H*), 5.64 (d, J = 7.5 Hz, 1H, *H*6), 5.32–5.26 (m, 1H, 3'-O*H*), 4.29–4.11 (m, 10H, Fc-*H*, 3'-*H*), 3.87 (d, J = 4.3 Hz, 1H, 4'-*H*), 3.73 (s, 6H, OCH_3), 3.20 (d, J = 3.9 Hz, 2H, N-CH_2), 2.18 (dq, J = 13.8, 8.0, 6.7 Hz, 1H, 2'-*H*), 2.04 (dt, J = 13.2, 6.5 Hz, 1H, 2'-*H*) ppm. ^{13}C NMR (101 MHz, DMSO-d_6) δ 162.7, 158.1, 154.9, 144.7, 139.6, 135.4, 135.3, 129.7, 129.6, 127.9, 127.8, 127.7, 126.7, 113.2, 113.1, 94.5, 85.7, 85.1, 85.1, 84.6, 70.0, 68.5, 68.4, 67.5, 63.4, 55.0, 54.9, 45.7, 40.4 ppm. MS (ESI) calcd for $C_{41}H_{41}FeN_3O_6$ 727.23, found $[M + H]^+$ 728.36.

5'-*O*-(4,4'-Dimethoxytrityl)-*N*^{4}-(4-(aminomethyl)benzyl)-trifluoroacetamide)-2'-deoxycytosine (A5^{BA})

Following the same procedure as synthesis of A5^{AD}, A4^{BA} (400 mg, 0.90 mmol)) reacted with 4, 4'-dimethoxytritylchloride to obtain the product. Yield:363.6 mg (54 %) TLC (CH_2Cl_2/ methanol 9:1): Rf = 0.36. ^{1}H NMR (400 MHz, $CDCl_3$-d_3) δ 8.82 (t, J = 6.0 Hz, 1H, N-*H*), 7.62 (d, J = 7.5 Hz, 1H, *H*5), δ 7.37–7.19 (m, 10H, Ar-*H*, N-*H*), 6.78 (d, J = 8.5 Hz, 4H, Ar-

H), 6.22 (t, *J* = 6.0 Hz, 1H, 1'-*H*), 5.80 (d, 1H, *H*6), 5.27 (m, 1H, 3'-O*H*), 4.53–4.36 (m, 5H, 3'-*H*, N-C*H*$_2$), 4.03 (m, 1H, 4'-*H*), 3.73 (s, 6H, OC*H*$_3$), 3.34 (m, 2H, 5'-*H*), 2.43 (m, 1H, 2'-*H*), 2.05 (m, 1H, 2'-*H*) ppm. ^{13}C NMR (101 MHz, $CDCl_3$-*d*$_3$) δ 163.9, 158.5, 156.8, 144.6, 139.5, 137.9, 136.0, 135.7, 135.6, 130.1, 128.2, 128.0, 128.0, 127.6, 127.0, 113.3, 95.9, 86.6, 85.8, 85.6, 71.1, 63.3, 55.3, 53.5, 45.9, 43.1, 41.7 ppm. MS(ESI) calcd for $C_{40}H_{39}F_3N_4O_7$ 744.28, found $[M + H]^+$ 745.36.

5'-*O*-(4,4'-Dimethoxytrityl)-*N*^{4}-(methyl(4-(tert-butyl)phenyl))-2'-deoxycytosine (A5^{TB})

Following the same procedure as synthesis of A5^{AD}, A4^{TB} (395.3 mg, 1.06 mmol)) reacted with 4, 4'-dimethoxytritylchloride to obtain the product. Yield: 422.1 mg (59 %) TLC (CH_2Cl_2/ methanol 9:1): Rf = 0.59. ^{1}H NMR (400 MHz, $CDCl_3$-*d*$_3$) δ 7.85 (d, *J* = 7.3 Hz, 1H , *H*5), 7.43–7.19 (m, 14H, N-*H*, Ar-*H*), 6.82 (d, *J* = 8.3 Hz, 4H, Ar-*H*), 6.36 (s, 1H, 1'-*H*), 5.30 (d, *J* = 8.3 Hz, 1H, *H*6), 5.05 (s, 1H, 3'-O*H*), 4.62 (m, 2H, N-C*H*$_2$), 4.52 (m, 1H, 3'-*H*), 4.16–4.03 (m, 1H, 4'-*H*), 3.78 (s, 6H, OC*H*$_3$), 3.47 (dd, *J* = 10.9 Hz, 1H , 5'-*H*), 3.39 (dd, *J* = 10.8 Hz, 1H , 5'-*H*), 2.61 (dt, *J* = 13.0, 5.2 Hz 1H, 2'-*H*), 2.24 (dt, *J* = 13.5, 6.2 Hz, 1H, 2'-*H*), 1.31 (d, *J* = 2.3 Hz, 9H, C(C*H*$_3$)$_3$) ppm. ^{13}C NMR (101 MHz, $CDCl_3$-*d*$_3$) δ 158.7, 150.9, 144.6, 140.4, 135.6, 130.2, 128.3, 128.2, 128.0, 127.1, 125.8, 113.3, 94.7, 86.8, 86.2, 85.9, 71.4, 63.1, 60.6, 55.4, 46.0, 44.9, 42.1, 34.7, 31.4, 21.2, 14.3, 8.9 ppm. MS(ESI) calcd for $C_{41}H_{45}N_3O_6$ 675.33, found $[M + H]^+$ 676.43.

3'-*O*-[(2-Cyanoethoxy-N,N-diisopropylamin)phosphinyl]-5'-*O*-(4,4'-dimethoxytrityl)-*N*^{4}-(1-methyladamantane)-2'-deoxycytosine (A6^{AD})

Compound A5^{AD} (338.6 mg, 0.50 mmol) was dissolved in 30 mL dry dichloromethane and stirred for ten minutes in an ice bath under N_2 atmosphere. N, N- diisopropylethylamine (349.9 µL, 2.0 mmol, 4 eq.) were added and the mixture was stirred for 10 minutes followed by the addition of 2-cyanoethoxy-N, N-diisopropylaminochlorophosphine (223.0 µL, 1.0 mmol, 2 eq.). The reaction mixture was stirred at room temperature for 1 hour under N_2 atmosphere, then diluted with 100 mL dichloromethane and washed with sat. aq. $NaHCO_3$. The organic layer was dried over Na_2SO_4 and removed under reduced pressure. The residue was purified by flash chromatography (petroleum ether/ethyl acetate 7:3→ 1:9) in the presence of 0.5 % NEt_3. Yield: 236.1 mg (60%) TLC ((petroleum ether/ethyl acetate=1:1): 0.55. ^{1}H NMR (400 MHz, $CDCl_3$-*d*$_3$) δ 7.86 (d, *J* = 7.4 Hz, 1H, *H*5), 7.45–7.36 (m, 2H, Ar-*H*), 7.34–7.20 (m, 8H, Ar-*H*), 6.87–6.79 (m, 4H, Ar-*H*), 6.33 (dt, *J* = 19.0, 5.8 Hz, 1H, 1'-*H*), 5.35–5.25 (m, 1H, *H*6), 4.67–4.58 (m, 1H, 3'-*H*), 4.10 (h, *J* = 3.8, 3.0 Hz, 1H, 4'-*H*), 3.79 (s, 6H, OC*H*$_3$), 3.65–3.46 (m, 4H,OC*H*$_2$CH$_2$CN, C*H*(CH$_3$)$_2$), 3.40–3.14 (m, 2H, 5'-*H*), 2.66–2.52 (m, 2H, N-C*H*$_2$, 2'-*H*), 2.40 (t, 2H, OCH$_2$C*H*$_2$CN), 2.32–2.19 (m, 1H, 2'-*H*), 2.01–1.95 (m, 3H , Ad-C*H*), 1.62–1.25 (m, 12H, Ad-C*H*$_2$), 1.25-

1.11 (m, 12H, CH(C*H*$_3$)$_2$) ppm. ^{13}C NMR (101 MHz, CDCl$_3$-*d$_3$*) δ 158.7, 144.6, 139.9, 135.6, 130.4, 128.4, 128.0, 127.1, 117.5, 113.3, 94.8, 86.7, 85.9, 85.1, 77.5, 77.2, 76.8, 62.3, 58.5, 58.3, 55.4, 52.1, 43.5, 43.3, 40.3, 37.0, 36.9, 33.8, 28.3, 28.2, 24.8, 24.7, 24.6, 20.3, 20.2 ppm. ^{31}P NMR (162 MHz, CDCl$_3$-*d$_3$*) δ 149.03 ppm. MS(ESI) calcd for $C_{50}H_{64}N_5O_7P$ 877.45, found $[M + H]^+$ 878.34.

3'-*O*-[(2-Cyanoethoxy-N,N-diisopropylamin)phosphinyl]-5'-*O*-(4,4'-dimethoxytrityl)-*N*^{4}-(1-methylferrocene)-2'-deoxycytosine (A6^{FC})

Following the same procedure as synthesis of A6^{AD}, A5^{FC} (363.1 mg, 0.5mmol), A6^{FC} was obtained and then purified via flash chromatography with petroleum ether/ethyl acetate 9:1→ 3:7 to give a yellow foam. Yield: 259.56 mg (56 %) TLC (petroleum ether/ethyl acetate 1:1): Rf = 0.58. ^{1}H NMR (400 MHz, CDCl$_3$-*d$_3$*) δ 7.92 (d, *J* = 7.4 Hz, 1H, *H*5), 7.44–7.33 (m, 2H, Ar-*H*), 7.33–7.19 (m, 7H, Ar-*H*), 6.84 (d, *J* = 8.3 Hz, 4H, Ar-*H*), 6.35 (d, *J* = 5.9 Hz, 1H, 1'-*H*), 5.21 (d, *J* = 7.4 Hz, 1H, *H*6), 4.99 (s, 1H, N-*H*), 4.65 (dt, *J* = 11.4, 6.2 Hz, 1H, 3'-*H*), 4.35 (d, *J* = 4.5 Hz, 1H, 4'-*H*), 4.24–4.05 (m, 9H, Fc-*H*), 3.78 (s, 6H, OC*H*$_3$), 3.57–3.45 (m, 6H,OC*H*$_2$CH$_2$CN, C*H*(CH$_3$)$_2$, 5'-*H*), 3.35 (m, 2H, N-C*H*$_2$), 2.66–2.54 (m, 1H, 2'-*H*), 2.40 (t, 2H, OCH$_2$C*H*$_2$CN), 2.33–2.21 (m, 1H, 2'-*H*), 1.29–1.12(m, 12H, CH(C*H*$_3$)$_2$) ppm. ^{13}C NMR (101 MHz, CDCl$_3$-*d$_3$*) δ 162.8, 158.7, 156.2, 144.5, 140.2, 135.6, 135.5, 130.3, 130.3, 128.4, 128.3, 128.0, 127.1, 117.5, 117.0, 113.3, 94.5, 86.8, 85.9, 85.1, 84.2, 77.5, 77.2, 76.8, 71.8, 71.6, 68.8, 68.7, 68.4, 67.5, 62.2, 58.5, 58.3, 58.2, 55.4, 55.4, 45.4, 45.4, 43.4, 43.3, 43.3, 43.2, 40.8, 40.5, 31.6, 30.2, 24.8, 24.7, 24.6, 24.6, 23.1, 23.0, 23.0, 20.3, 20.2, 20.2 ppm. ^{31}P NMR (162 MHz, CDCl$_3$-*d$_3$*) δ 149.08 ppm. MS(ESI) calcd for $C_{50}H_{58}FeN_5O_7P$ 927.34, found $[M + H]^+$ 928.40.

3'-*O*-[(2-Cyanoethoxy-N,N-diisopropylamin)phosphinyl]-5'-*O*-(4,4'-dimethoxytrityl)-*N*^{4}-(4-(aminomethyl)benzyl)-trifluoroacetamide)-2'-deoxycytosine (A6^{BA})

Following the same procedure as synthesis of A6^{AD}, A5^{BA} (297.6 mg, 0.40mmol), A6^{An} was obtained and then purified via flash chromatography with petroleum ether/ethyl acetate 7:3→ 1:9 to give a white foam. Yield: 207.7mg (55 %) TLC (petroleum ether/ethyl acetate 1:1): Rf = 0.28. ^{1}H NMR (400 MHz, CDCl$_3$-*d$_3$*) δ 7.80 (d, *J* = 7.4 Hz, 1H, *H*5), 7.53 (m, 1H, N-*H*), 7.39 (d, *J* = 7.5 Hz, 2H, Ar-*H*), 7.24 (d, *J* = 22.8 Hz, 11H, Ar-*H*), 6.81 (d, *J* = 8.4 Hz, 4H, Ar-*H*), 6.33 (d, *J* = 6.0 Hz, 1H, 1'-*H*), 5.51 (m, 1H, N-*H*), 5.36 (d, *J* = 7.4 Hz, 1H, *H*6), 4.61 (dd, *J* = 10.8, 4.9 Hz, 1H, 3'-*H*), 4.56–4.44 (m, 4H, N-C*H*$_2$), 4.11 (dd, *J* = 5.5, 3.0 Hz, 1H, 4'-*H*), 3.78 (s, 6H, OC*H*$_3$), 3.61–3.25 (m, 5H, OC*H*$_2$CH$_2$CN, C*H*(CH$_3$)$_2$, 5'-*H*), 2.69–2.54 (m, 3H, 2'-*H*, OCH$_2$C*H*$_2$CN), 2.33–2.19 (m, 1H, 2'-*H*), 1.38–1.01(m, 12H, CH(C*H*$_3$)$_2$) ppm. ^{13}C NMR (101 MHz, CDCl$_3$-*d$_3$*) δ 158.7, 157.6, 144.5, 135.7, 135.6, 135.5, 130.3, 128.5, 128.3, 128.0, 127.1, 117.8, 113.3, 86.8, 77.2, 58.4, 58.2,

55.4, 43.5, 43.4, 43.2, 24.7, 24.7, 24.6, 24.6, 20.5, 20.4 ppm. ^{31}P NMR (162 MHz, $CDCl_3$-d_3) δ 148.62, 148.56, 148.50 ppm. MS(ESI) calcd for $C_{49}H_{56}F_3N_6O_8P$ 944.38, found $[M + H]^+$ 945.39.

3'-*O*-[(2-Cyanoethoxy-N,N-diisopropylamin)phosphinyl]-5'-*O*-(4,4'-dimethoxytrityl)-*N*⁴-(4-(methyl(4-(tert-butyl)phenyl))-2'-deoxycytosine ($A6^{TB}$)

Following the same procedure as synthesis of $A6^{AD}$, $A5^{TB}$ (337.7mg, 0.50mmol)), $A6^{TB}$ was obtained and then purified via flash chromatography with petroleum ether/ethyl acetate 9:1→ 3:7 to give a white foam. Yield: 262.5mg (60 %) TLC (petroleum ether/ethyl acetate 1:1): Rf = 0.53. ^1H NMR (400 MHz, $CDCl_3$-d_3) δ 7.83 (d, *J* = 7.3 Hz, 1H, *H*5), 7.37 (dd, *J* = 8.4, 2.2 Hz, 2H, Ar-*H*), 7.31–7.19 (m, 11H, Ar-*H*), 6.81 (d, *J* = 8.3 Hz, 4H, Ar-*H*), 6.40 (d, *J* = 7.4 Hz, 1H, 1'-*H*), 5.28 (d, *J* = 14.2 Hz, 1H, *H*6), 5.05 (s, 1H, N-*H*), 4.61 (dd, *J* = 15.7, 7.4 Hz, 1H, 3'-*H*), 4.11 (q, *J* = 3.5 Hz, 1H, 4'-*H*), 3.78 (s, 6H, OCH_3), 3.61–3.27 (m, 5H, OCH_2CH_2CN, $CH(CH_3)_2$, 5'-*H*), 2.73–2.58 (m, 3H, 2'-*H*, OCH_2CH_2CN), 2.30–2.19 (m, 1H, 2'-*H*), 1.31 (s, 9H, $C(CH_3)_3$), 1.21–1.07(m, 12H, $CH(CH_3)_2$) ppm. ^{13}C NMR (101 MHz, $CDCl_3$-d_3) δ 163.4, 158.7, 156.2, 150.9, 144.5, 140.3, 135.6, 134.9, 130.3, 128.3, 128.2, 128.0, 127.1, 125.8, 117.7, 113.3, 94.7, 86.8, 86.0, 85.1, 72.9, 72.8, 62.7, 58.5, 58.3, 55.4, 44.9, 43.4, 43.2, 41.1, 34.7, 34.6, 31.4, 29.8, 24.7, 24.7, 24.6, 24.6, 23.5, 20.5, 20.4 ppm. ^{31}P NMR (162 MHz, $CDCl_3$-d_3) δ 148.56 ppm. MS(ESI) calcd for $C_{50}H_{62}N_5O_7P$ 875.44, found $[M + H]^+$ 876.42.

Path B:

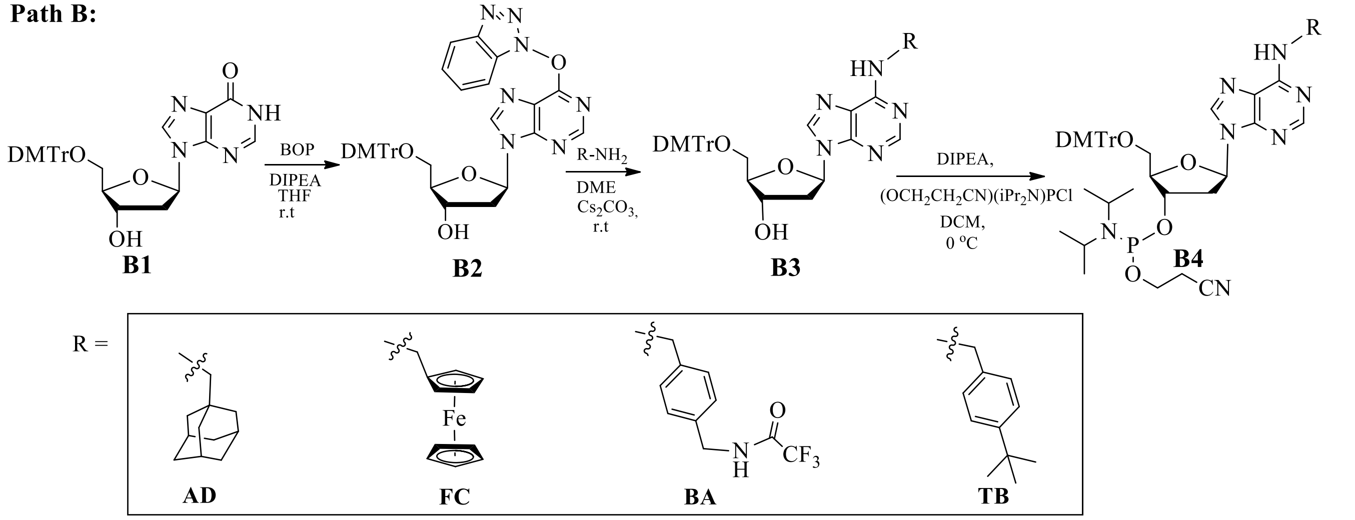

In Path B, synthesis of the modified adenine was performed through a more facile approach in which the deoxyribose was not necessary to be protected during conjugation of these amine compounds as reported previously.[1]

5'-*O*-(4, 4'-dimethoxytrityl)-2′-deoxyinosine(B1)

Compound B1 was synthesized according to the previously reported method.[2] ^1H NMR (400 MHz, DMSO-d_6) δ 12.37 (s,

1H, N-*H*), 8.19 (s, 1H, *H*8), 7.99 (s, 1H, *H*2), 7.33–7.32 (m, 2H, Ar-*H*), 7.25–7.18 (m, 7H, Ar-*H*), 6.80 (t,4H, Ar-*H*), 6.34 (s, 1H, 1'-*H*), 5.38 (s, 1H, 3'-O*H*), 4.43 (br m, 1H, 3'-H), 3.98 (q, 1H, 4'-*H*), 3.72 (s, 6H, OCH_3), 3.19–3.11 (m, 2H, 5'-*H*), 2.75 (m, 1H, 2'-*H*), 2.33 (m, 1H, 2'-*H*) ppm. ^{13}C NMR (101 MHz, DMSO-d_6) δ 158.0, 158.0, 156.6, 147.9, 145.6, 144.9, 138.7, 135.6, 135. 5, 129.7, 129.6, 127.7, 127.7,126.6, 124.7, 113.1, 85.9, 85.4, 83. 5, 70.5, 64.1, 55.0 ppm. MS(ESI) calcd for $C_{31}H_{30}N_4O_6$ 554.22, found $[M + H]^+$ 555.32.

5'-*O*-(4,4'-dimethoxytrityl)-*O*^{6}-(Benzotriazol-1-yl)-2'-deoxyinosine (B2).

Compound B1 (5.0 g, 9.03 mmol) was dissolved in 300 mL dry THF. Under N_2 atmosphere, BOP (8.85 g, 20.0 mmol), *N*, *N*- diisopropylethylamine (3.48 mL, 20 mmol) was added into the mixture and the solution was stirred for 40 hours at room temperature. The reaction mixture was diluted with ethyl acetate (1 L), then washed with sat. aq. NaCl, and dried with Na_2SO_4. The product was purified via flash chromatography with petroleum ether/ethyl acetate 7:3→ 3:7 in the presence of 0.5 % NEt_3 to give a white foam. Yield: 4.06g (67 %) The column was packed with the initial solvent containing 0.5 % NEt_3. (ethyl acetate): Rf = 0.40. ^{1}H NMR (400 MHz, $CDCl_3$-d_3) δ 8.34–8.22 (m, 2H, *H*8, *H*2), 8.13 (d, J = 8.3 Hz, 1H, Ar-*H*), 7.58–7.51 (m, 1H, Ar-*H*), 7.51–7.42 (m, 2H, Ar-*H*), 7.39 (d, J = 8.2 Hz, 2H, Ar-*H*), 7.32–7.16 (m, 8H, Ar-*H*), 6.80 (d, J = 8.4 Hz, 4H, Ar-*H*), 6.53 (t, *J* = 6.4 Hz, 1H, 1'- *H*), 4.76–4.70 (m, 1H, 3'- *H*), 4.20 (q, J = 4.4 Hz, 1H, 4'- *H*), 3.77 (s, 6H, OCH_3), 3.49–3.36 (m, 2H , 5'- *H*), 2.86 (dt, J = 13.0, 6.4 Hz, 1H, 2'- *H*), 2.62 (ddd, J = 13.4, 6.2, 4.2 Hz, 1H, 2'- *H*), 2.52 (d, J = 18.8 Hz, 1H, 3′-O*H*) ppm. ^{13}C NMR (101 MHz, $CDCl_3$-d_3) δ 159.1, 158.7, 153.6, 151.5, 144.5, 143.7, 143.6, 135.6, 135.6, 130.1, 129.0, 128.9, 128.2, 128.1, 127.2, 125.1, 120.6, 120.1, 113.3, 108.8, 86.8, 86.5, 85.2, 72.6, 63.7, 55.4, 40.5 ppm. MS(ESI) calcd for $C_{37}H_{34}N_7O_6$ 671.70, found $[M + H]^+$ 672.25.

5'-*O*-(4,4'-dimethoxytrityl)-*N*^{6}- (1-methyladamantane)-2'-deoxyadenosine (B3^{AD}).

Compound B2 (1.0 g, 1.48 mmol) and 1-aminomethyladamantane (394.3 μL, 2.22 mmol, 1.5 eq.), were dissolved in 50 mL dry DME. Cs_2CO_3 (1.43 g, 4.4 mmol, 3.0 eq.) was added into the mixture and the solution was stirred for 12 hours at room temperature. After filtration and evaporation, the residue was dissolved in ethyl acetate, then washed with sat. aq. NaCl, and dried with Na_2SO_4. purified by flash chromatography (dichloromethane/ methanol 100:0→98:2→94:6) to give a white solid. Yield: 0.70 g (67 %) TLC (CH_2Cl_2/ methanol 14:1): Rf = 0.48. ^{1}H NMR (400 MHz, $CDCl_3$-d_3) δ 8.29 (s, 1H, *H*8), 7.90 (s, 1H, *H*2), 7.38 (d, *J* = 7.4 Hz, 2H, Ar-*H*), 7.32–7.14 (m, 7H, Ar-*H*), 6.83–6.74 (m, 4H, Ar-*H*), 6.43 (t, *J* = 6.4 Hz, 1H, 1'-*H*), 5.92 (s, 1H, N-*H*), 4.63 (dt, *J* = 6.6, 4.0 Hz, 1H, 3'-*H*), 4.17–4.08 (m, 1H, 4'-*H*), 3.76 (s, 6H, OCH_3), 3.36 (dd, *J* = 11.1, 4.9 Hz, 2H, 5'-*H*), 2.77 (dt, *J* = 13.1, 6.4 Hz, 1H, 2'-*H*), 2.50 (ddd, *J* = 13.4, 6.2, 4.1 Hz, 1H, 2'-*H*), 2.29 (s,

3H, 3′-O*H*, N-CH_2), 2.06–1.94 (m, 3H, Ad-C*H*), 1.71 (dd, *J* = 12.2, 3.5 Hz, 12H, Ad-CH_2) ppm. ^{13}C NMR (101 MHz, $CDCl_3$-*d₃*) δ 158.6, 155.6, 153.2, 144.6, 137.9, 135.8, 135.8, 130.1, 130.1, 128.2, 128.0, 127.0, 113.3, 86.6, 86.2, 84.3, 63.9, 55.3, 55.1, 52.0, 40.5, 40.3, 40.1, 37.3, 37.1, 34.2, 34.0, 28.5, 28.4 ppm. MS(ESI) calcd for $C_{42}H_{47}N_5O_5$ 701.36, found [M + H]$^+$ 702.50.

5’-*O*-(4,4’-dimethoxytrityl)-*N*^{6}-(1-methylferrocene)-2’-deoxyadenosine (B3^{FC})

Following the same procedure as synthesis of B3^{AD}, ferrocenyl methylamine (457.3 mg, 2.22mmol, 1.5 eq.) reacted with Compound B2 (1.0 g, 1.48 mmol) to obtain the product. Yield: 0.67 g (61 %) TLC (CH_2Cl_2/ methanol 14:1): Rf = 0.55. ^{1}H NMR (400 MHz, $CDCl_3$-d_3) δ 8.34 (s, 1H, *H*8), 7.88 (s, 1H, *H*2), 7.42–7.34 (m, 2H, Ar-*H*), 7.32–7.13 (m, 7H, Ar-*H*), 6.82–6.74 (m, 4H, Ar-*H*), 6.42 (t, *J* = 6.4 Hz, 1H, 1’-*H*), 6.10(m, 1H, N-*H*), 4.69–4.60 (m, 1H, 3’-*H*), 4.49 (m, 2H, N-CH_2), 4.28–4.08 (m, 10H, Fc-*H*, 4’-*H*), 3.75 (s, 6H, OCH_3), 3.38 (q, *J* = 3.0, 2.1 Hz, 2H, 5’-*H*), 2.76 (dq, *J* = 11.6, 5.9, 5.4 Hz, 1H, 2’-*H*), 2.55–2.44 (m, 1H, 2’-*H*), 1.81(d, 1H, 3′-O*H*) ppm. ^{13}C NMR (101 MHz, $CDCl_3$-d_3) δ 158.7, 154.3, 153.2, 144.6, 138.2, 135.8, 135.8, 130.1, 128.2, 128.2, 128.0, 127.1, 120.2, 113.3, 86.7, 86.0, 85.2, 84.2, 72.6, 68.7, 68.5, 68.3, 63.9, 60.6, 55.3, 40.5 ppm. MS(ESI) calcd for $C_{42}H_{41}FeN_5O_5$ 751.25, found [M + H]$^+$ 752.36.

5’-*O*-(4,4’-dimethoxytrityl)-*N*^{6}-(4-(aminomethyl)benzyl) -trifluoroacetamide)-2’-deoxyadenosine (B3^{BA})

Following the same procedure as synthesis of B3^{AD}, N-(4-(aminomethyl)benzyl) -trifluoroacetamide (515.0mg, 2.22 mmol, 1.5 eq.) was reacted with Compound B2 (1.0 g, 1.48 mmol) to obtain the product. Yield: 0.68 g (60 %) TLC (CH_2Cl_2/ methanol 14:1): Rf = 0.32. ^{1}H NMR (400 MHz, DMSO-d_6) δ 9.99–9.91 (m, 1H, N-*H*), 8.26 (s, 1H, *H*8), 8.13 (s, 1H, *H*2), 7.32 (t, *J* = 7.2 Hz, 4H, Ar-*H*), 7.25–7.11 (m, 9H, Ar-*H*), 6.85–6.74 (m, 4H, Ar-*H*), 6.40 (dt, *J* = 21.0, 6.3 Hz, 1H, 1’-*H*), 5.75 (s, 1H, N-*H*), 4.71–4.65 (m, 1H, 3’-*H*), 4.48 (m, 2H, N-CH_2), 4.34 (m, 2H, N-CH_2), 3.98 (q, *J* = 4.7 Hz, 1H, 4’-*H*), 3.71 (s, 6H, OCH_3), 3.23–3.11 (m, 2H, 5’-*H*), 2.93–2.82 (m, 1H, 2’-*H*), 2.32 (ddd, *J* = 13.2, 6.7, 4.5 Hz, 1H, 2’-*H*) ppm. ^{13}C NMR (101 MHz, DMSO-d_6) δ 158.0, 156.5, 156.1, 152.4, 144.9, 135.8, 135.7, 135.5, 129.7, 127.7, 127.7, 127.4, 127.4, 127.2, 127.2, 126.6, 117.5, 113.1, 85.8, 85.4, 83.4, 70.7, 64.1, 55.0, 54.9, 45.7, 42.4 ppm. MS(ESI) calcd for $C_{41}H_{39}F_3N_6O_6$ 768.29, found [M + H]$^+$ 769.34.

5’-*O*-(4,4’-dimethoxytrityl)-*N*^{6}-(methyl(4-(tert-butyl)phenyl))-2’-deoxyadenosine(B3^{TB})

Following the same procedure as synthesis of B3^{AD}, (4-(tert-butyl)phenyl)methanamine (389.1 μL, 2.22 mmol, 1.5 eq.) was reacted with Compound B2 (1.0 g, 1.48 mmol) to obtain the product. Yield: 0.64 g (62 %) TLC (CH_2Cl_2/ methanol

14:1): Rf = 0.48. ^{1}H NMR (400 MHz, $CDCl_3$-*d_3*) δ 8.34 (s, 1H, *H*8), 7.87 (s, 1H, *H*2), 7.41–7.12 (m, 13H, Ar-*H*), 6.82–6.73 (m, 4H, Ar-*H*), 6.42 (t, J = 6.5 Hz, 1H, 1'-*H*), 4.80 (s, 1H, N-*H*), 4.64 (dt, J = 6.2, 3.9 Hz, 1H, 3'-*H*), 4.16–4.06 (m, 1H, 4'-*H*), 3.75 (s, 6H, OC*H_3*), 3.43–3.31 (m, 2H, 5'-*H*), 2.76 (dt, *J* = 13.1, 6.4 Hz, 1H, 2'-*H*), 2.58–2.43 (m, 1H, 2'-*H*), 1.29 (s, 9H, C(C*H_3*)$_3$ ppm. ^{13}C (101 MHz, $CDCl_3$-*d_3*) δ 158.7, 155.8, 1533, 150.7, 144.7, 138.3, 135.9, 135.8, 135.5, 130.2, 128.3, 128.1, 127.1, 125.8127.9, 127.0, 124.9, 120.5, 120.0, 113.4, 108.7, 86.8, 86.2, 84.4, 72.6, 64.0, 55.4, 46.3, 40.6. 34.7, 31.5 ppm. MS(ESI) calcd for $C_{42}H_{45}N_5O_5$ 699.34, found $[M + H]^+$ 700.36.

3'-*O*-[(2-Cyanoethoxy-N,N-diisopropylamin)phosphinyl]-5'-*O*-(4,4'-dimethoxytrityl)-*N*^{6}-(1-methyladamantane) - 2'-deoxyadenosine (B4^{AD}).

Compound B3^{AD} (500 mg, 0.71 mmol) was dissolved in 30 mL dry dichloromethane and stirred for ten minutes in an ice bath under N_2 atmosphere. 110 µL N, N-diisopropylethylamine (496.9 µL, 2.84 mmol, 4 eq.) were added and the mixture was stirred for 10 minutes followed by the addition of 2-cyanoethoxy-N, N-diisopropylaminochlorophosphine (316.7 µL, 1.42 mmol, 2 eq.). The reaction mixture was stirred at room temperature for 1 hour under N_2 atmosphere, then diluted with 100 mL dichloromethane and washed with sat. aq. $NaHCO_3$. The organic layer was dried over Na_2SO_4 and removed under reduced pressure. The residue was purified by flash chromatography (petroleum ether/ethyl acetate 7:3→ 1:9) in the presence of 0.5 % NEt_3. Yield: 371.0 mg (58%) TLC ((petroleum ether/ethyl acetate=1:1): Rf = 0.50. ^{1}H NMR (400 MHz, $CDCl_3$-*d_3*) δ 8.30 (s, 1H, *H*8), 7.94 (s, 1H, *H*2),7.39 (d, *J* = 7.6 Hz, 2H, Ar-*H*), 7.32–7.14 (m,7H, Ar-*H*), 6.79 (d, *J* = 8.4 Hz, 4H, Ar-*H*), 6.41 (dd, J = 12.7, 6.1 Hz, 1H, 1'-*H*), 4.76 (br m, 1H, 3'-*H*), 4.27 (q, J = 4.1 Hz, 1H, 4'-*H*), 3.77 (s, 6H, OC*H_3*), 3.74–3.49 (m, 6H, OC*H_2*CH_2CN, *i*-Pr-C*H*, C-N*H_2*), 3.47–3.27 (m, 2H, 5'-*H*), 2.86 (dt, J = 13.3, 7.0 Hz, 1H, 2'-*H*), 2.67 (td, J = 11.6, 10.0, 6.8 Hz, 1H, N-*H*), 2.63–2.40 (m, 5H, OCH_2C*H_2*CN), 2.36 (s, 1H, 2'-*H*), 2.20–1.47 (m, 18H, Ad-H, C*H_3*), 1.39–1.06 (m, 12H, *i*-Pr-C*H_3*) ppm. ^{13}C NMR (101 MHz, $CDCl_3$-*d_3*) δ 158.6, 155.6, 153.2, 144.7, 138.0, 135.9, 135.8, 130.2, 130.2, 128.3, 127.9, 127.0, 117.6, 113.2, 86.5, 85.9, 85.9, 84.5, 73.7, 73.5, 63.4, 58.6, 58.4, 55.3, 53.2, 44.2, 44.1, 40.4, 37.2, 37.1, 37.1, 28.4, 28.4, 24.7, 23.7 ppm. ^{31}P NMR (162 MHz, $CDCl_3$-*d_3*) δ 148.76 ppm. MS(ESI) calcd for $C_{51}H_{61}N_7O_6P$ 901.47, found $[M + H]^+$ 902.42.

3'-*O*-[(2-Cyanoethoxy-N,N-diisopropylamin)phosphinyl]-5'-*O*-(4,4'-dimethoxytrityl)-*N*^{6}-(1-methylferrocene)-2'-deoxyadenosine (B4^{FC}).

Following the same procedure as synthesis of B4^{AD}. B3^{FC} (533.2 mg, 0.71 mmol), Yield: 371.4 mg (55 %) TLC ((petroleum ether/ethyl acetate=1:1): Rf = 0.55. ^{1}H NMR (400 MHz, $CDCl_3$-*d_3*) δ 8.36 (s, 1H, *H*8), 7.95 (s, 1H, *H*2), 7.44–7.35 (m, 2H,

2H, Ar-*H*), 7.31–7.20 (m,7H, Ar-*H*), 6.84–6.74 (m, 4H, Ar-*H*), 6.43 (t, *J* = 6.6 Hz, 1H, 1'-*H*), 5.98 (m, 1H, N-*H*), 4.76 (ddt, *J* = 9.9, 6.4, 3.4 Hz, 1H, 3'-*H*), 4.54 - 4.48 (m, 1H, 4'-*H*), 4.33–4.07 (m, 9H, Fc-*H*) 3.77 (s, 6H, OCH_3,), 3.75– 3.49 (m, 7H, OCH_2CH_2CN, *i*-Pr-C*H*, N-CH_2), 3.43 (dd, *J* = 10.4, 4.1 Hz, 1H, 5'-*H*), 3.33 (dd, *J* = 10.4, 4.5 Hz, 1H, 5'-*H*), 2.98–2.81 (m, 1H, 2'-*H*), 2.58 (ddt, *J* = 12.0, 6.1, 2.8 Hz, 1H, 2'-*H*), 2.42 (m, 2H, OCH_2CH_2CN), 1.18 (m, 12H, i-Pr-CH_3) ppm. ^{13}C NMR (101 MHz, $CDCl_3$-d_3) δ 158.6, 154.4, 153.2, 144.7, 138.4, 135.8, 135.8, 130.2, 130.2, 128.3, 127.9, 127.0, 120.3, 117.6, 113.2, 86.5, 85.9, 85.9, 85.3, 84.5, 73.6, 73.5, 68.7, 68.5, 68.3, 63.4, 60.5, 58.6, 58.4, 58.0, 55.4, 43.5, 43.3, 39.6, 39.6, 24.8, 24.7, 24.7, 20.4, 20.3 ppm. ^{31}P NMR (162 MHz, $CDCl_3$-d_3) δ 148.78 ppm. MS(ESI) calcd for $C_{51}H_{58}FeN_7O_6P$ 951.35, found $[M + H]^+$ 952.16.

3'-*O*-[(2-Cyanoethoxy-N,N-diisopropylamin)phosphinyl]-5'-*O*-(4,4'-dimethoxytrityl)-*N*⁶-(4-(aminomethyl)benzyl) -trifluoroacetamide) -2'-deoxyadenosine (B4 ^{BA}).

Following the same procedure as synthesis of B4^{AD}. B3 ^{BA} (545.3 mg, 0.71 mmol), B4 ^{BA} Yield: 364.4 mg (53 %) TLC ((petroleum ether/ethyl acetate=1:1): Rf = 0.33. ^{1}H NMR (400 MHz, $CDCl_3$-d_3) δ 8.32(s, 1H, *H*8), 7.93 (s, 1H, *H*2), 7.33–7.30 (m, 4H, Ar-*H*), 7.22–7.12 (m, 9H, Ar-*H*), 6.96 (m, 1H, N-*H*), 6.75 (d, 4H, Ar-*H*,), 6.39 (t, 1H, 1'-*H*), 4.83 (m, 1H, 3' -*H*), 4.76 (m, 1H, N-*H*), 4.48 (m, 2H, N-CH_2), 4.14(m, 2H, N-CH_2), 3.83(m, 1H, 4' -*H*), 3.76 (s, 6H, OCH_3), 3.75–3.31 (m, 6H, OCH_2CH_2CN, i-Pr-C*H*, 5'-*H*), 2.98–2.81 (m, 1H, 2'-*H*), 2.58–2.43 (m, 3H, 2'-*H*, OCH_2CH_2CN),), 1.27-1.01 (d, 12H, i-Pr-CH_3) ppm. ^{13}C NMR (101 MHz, $CDCl_3$-d_3) δ 158.6, 154.7, 153.2, 144.6, 138.9, 138.5, 135.8, 135.2, 130.2, 130.2, 128.4, 128.3, 128.3, 127.9, 127.0, 127.0, 120.2, 117.6, 117.0, 113.2, 86.5, 85.9, 84.5, 84.4, 74.3, 74.1, 73.5, 73.4, 63.5, 63.3, 58.5, 58.4, 58.3, 58.2, 57.9, 55.3, 55.3, 45.5, 45.4, 43.7, 43.4, 43.4, 43.3, 43.3, 39.7, 24.8, 24.8, 24.7, 24.7, 24.7, 24.6, 23.1, 23.0, 23.0, 21.6, 20.6, 20.5, 20.4, 20.3, 20.3, 20.2 ppm. ^{31}P NMR (162 MHz, $CDCl_3$-d_3) δ 148.77, 148.69 ppm. MS(ESI) calcd for $C_{50}H_{56}F_3N_8O_7P$ 968.40, found $[M + H]^+$ 969.32.

3'-*O*-[(2-Cyanoethoxy-N,N-diisopropylamin)phosphinyl]-5'-*O*-(4,4'-dimethoxytrityl)-*N*⁶-(methyl(4-(tert-butyl)phenyl))-2'-deoxyadenosine(B4^{TB})

Following the same procedure as synthesis of B4^{AD}. B3 ^{TB} (496.3 mg, 0.71 mmol), B4 ^{TB} Yield: 383.0mg (60 %) TLC ((petroleum ether/ethyl acetate=1:1): Rf = 0.45. ^{1}H NMR (400 MHz, $CDCl_3$-d_3) δ 8.36(s, 1H, *H*8), 7.94 (s, 1H, *H*2), 7.41–7.17 (m, 13H, Ar-*H*), 6.78 (d, 4H, Ar-*H*), 6.41 (t, 1H, 1'-*H*), 4.82 (m, *2*H, 3'-*H*, N-*H*), 4.29 (s, 1H, 4'-*H*), 3.77 (s, 6H, OCH_3), 3.67–3.58 (m, 6H, OCH_2CH_2CN, *i*-Pr-C*H*, N-CH_2), 3.42–3.36.(dd 2H, 5'-*H*), 2.91–2.84 (m, 1H, 2'-*H*), 2.64–2.56 (m, 1H, 2'-*H*), 2.43 (m, 2H, OCH_2CH_2CN), 1.31 (s, 9H, $C(CH_3)_3$), 1.16 (d, 12H, *i*-Pr-CH_3) ppm. ^{13}C NMR (101 MHz,

$CDCl_3$-d_3) δ 158.6, 153.3, 150.6, 144.7, 138.4, 135.8, 135.8, 135.5, 130.2, 130.2, 128.3, 128.0, 127.7, 127.0, 125.7, 120.3, 117.6, 113.2, 86.5, 85.9, 85.9, 84.5, 73.7, 73.5, 63.4, 58.6, 58.4, 55.4, 43.4, 43.3, 39.6, 39.6, 34.6, 31.5, 24.8, 24.7, 24.7, 20.4, 20.3 ppm. ^{31}P NMR (162 MHz, $CDCl_3$-d_3) δ 148.77 ppm. MS(ESI) calcd for $C_{51}H_{62}N_7O_6P$ 899.45, found $[M + H]^+$ 900.84.

Supplementary Tables

Supplementary Table 1. Sequences of ODNs used in this study.

Name	Sequence	Experiment
15- **C***	5'- ACACTGT**C***ACACTGC -3'	ITC
15- **A***	5'- ACACTGTC**A***CACTGC -3'	
15- $\mathbf{C_F}$*	5' (**FAM**)- ACACTGT**C***ACACTGC -3'	T_m
15- $\mathbf{A_F}$*	5' (**FAM**)- ACACTGTC**A***CACTGC -3'	
15-cODN	5'- GCAGTGTGACAGTGT (**BHQ**$_1$) -3'	
19-$\mathbf{C_F}$*	5' (**FAM**)- CGATGA**C***TGAGCA**C***TTCGT-3'	
19-cODN	5'- ACGAAGTGCTCAGTCATCG(**BHQ**$_1$) -3'	
Hp-1	5'(**TMR**)- ACGTCGCACACAGGAAACGT(**BHQ**$_2$) -3'	Duplex-hairpin conversion
H1-cODN-$\mathbf{C^{AD}}$	5'- CGTTTCC$\mathbf{C^{AD}}$TGTGTGC$\mathbf{C^{AD}}$GACGT -3'	
Hp-2	5'(**FAM**)- ACGAAGTGCTCAGTCATCGT(**BHQ**$_1$) -3'	
H2-cODN -$\mathbf{C^{FC}}$	5'- CGATGA$\mathbf{C^{FC}}$TGAGCA$\mathbf{C^{FC}}$TTCGT -3'	
15-R-$\mathbf{C_F^{AD}}$	5'(**FAM**)- ACA$\mathbf{C^{AD}}$TGT$\mathbf{C^{AD}}$ACA$\mathbf{C^{AD}}$TGC -3'	Reversible manipulation of duplex dissociation and recovery
15-R-$\mathbf{C_F^{FC}}$	5'(**FAM**)- ACA$\mathbf{C^{FC}}$TGT$\mathbf{C^{FC}}$ACA$\mathbf{C^{FC}}$TGC -3'	
15-cODN	5'- GCAGTGTGACAGTGT (**BHQ**$_1$) -3'	
DNAzyme	5′- CATACCAGTTGCCAGCGGCTCGAAAGTCTCCTTG-3'	Controllable DNAzyme
DNAzyme-C^{AD}	5'- CATACC$\mathbf{C^{AD}}$AGTTGCCAGCGGCTCGAAAGT$\mathbf{C^{AD}}$TCCTTG -3'	
DNAzyme-C^{FC}	5'- CATACC$\mathbf{C^{FC}}$AGTTGCCAGCGGCTCGAAAGT$\mathbf{C^{FC}}$TCCTTG -3'	
Sub-rA	5'(**FAM**)- CAAGGAGACT**rA**GACTGGGTATG- 3'	
Unmodified**	5'- GAGCTGCACGCTGCCGTC -3'	ASO experiments
AD-modified**	5'- GAG$\mathbf{C^{AD}}$TGCA$\mathbf{C^{AD}}$GCTG$\mathbf{C^{AD}}$CGTC -3'	

[*] Including all four types of guest modifications. [**] Phosphorothioate bonds were introduced in these ASOs.

Supplementary Table 2. Melting temperatures (T_m) of DNA duplexes with guest-modified adenine by treatment of CB[7].

ODN[a]	5'- ACACTGTC **A*** CACTGC -3'							
CB[7] / μM	AD		FC		BA		TB	
	T_m[b]	ΔT_m	T_m	ΔT_m	T_m	ΔT_m	T_m	ΔT_m
0	51.5	--	52.2	--	54.0	--	51.6	--
1	45.4	− 6.1	52.2	− 0	54.0	0	51.6	0
3	44.5	− 7.0	51.4	− 0.8	54.0	0	51.6	0
10	44.0	− 7.5	50.4	− 1.5	53.9	− 0.1	51.3	− 0.3
30	43.7	− 7.8	48.8	− 3.4	53.9	− 0.1	51.3	− 0.3
100	43.7	− 7.8	46.0	− 6.1	53.8	− 0.2	51.0	− 0.6
300	43.1	− 8.4	43.4	− 8.8	52.9	− 1.1	50.9	− 0.7

[a] DNA duplex was formed with the unmodified complementary strand in the presence of 20 mM sodium cacodylate (pH = 7.2), 100 mM NaCl and 0.1 mM EDTA. [b] The unit is °C. All the T_m values were obtained from two independent replicates.

Supplementary Figures

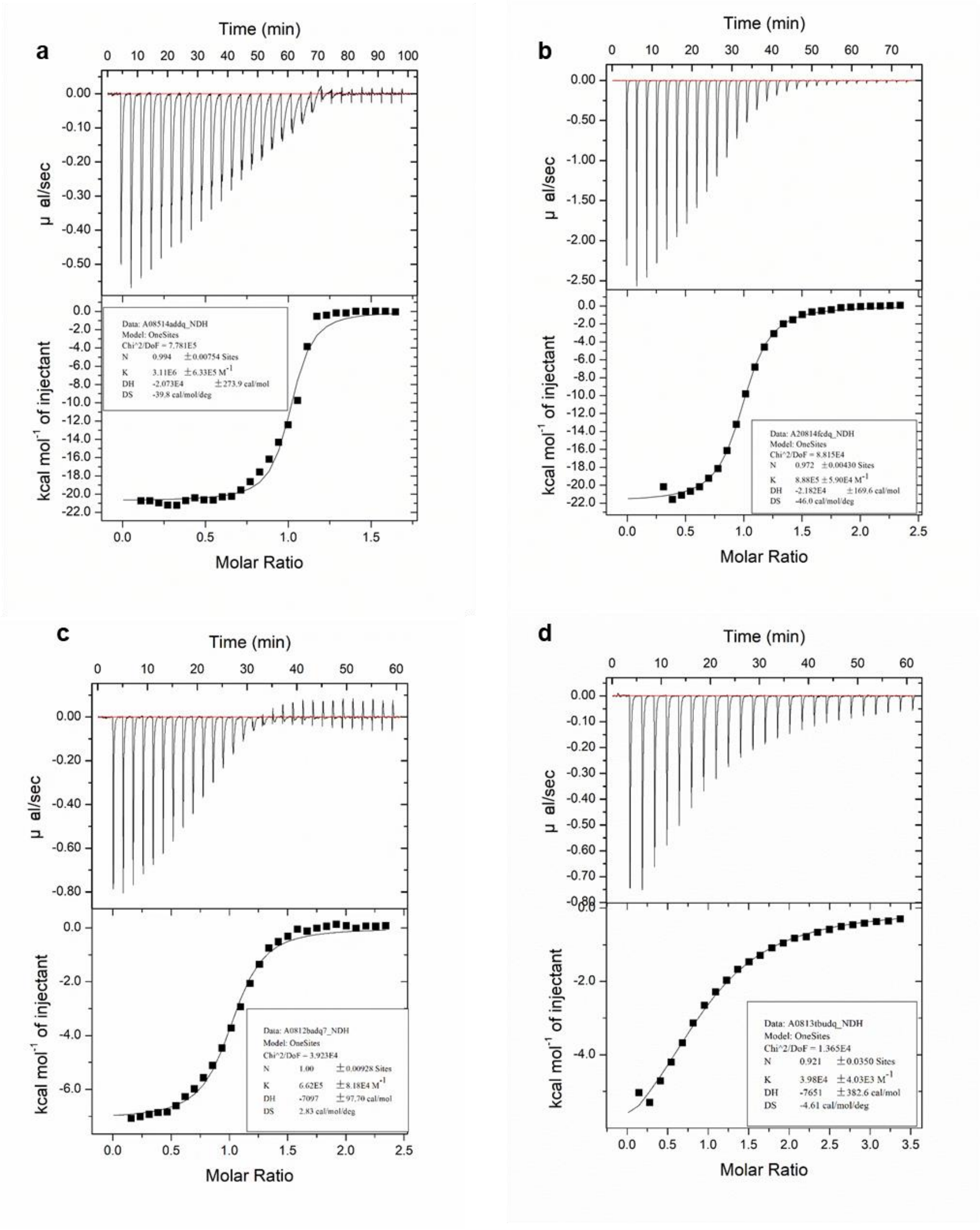

Supplementary Fig. 1. ITC determination of CB7 binding affinities to the guest molecules in the buffer solution. (**a**) Binding with the guest AD; calculated $K_a = K_{ADexp} \times K_{BA} \times [BA] = (3.11 \times 10^6) \times (6.62 \times 10^5) \times (8 \times 10^{-4})\ M^{-1} = 1.64 \times 10^9\ M^{-1}$. (**b**) Binding with the guest FC; calculated $K_a = (8.88 \times 10^5) \times (6.62 \times 10^5) \times (8 \times 10^{-4})\ M^{-1} = 4.70 \times 10^8\ M^{-1}$. The competitive method (taking BA as the competing guest) was utilized to measure the binding constants of CB[7] on AD and FC. (**c**) Binding with the guest BA; calculated $K_a = 8.88 \times 10^5\ M^{-1}$. (**d**) Binding with the guest TB; calculated

K_a = 3.98 × 10^4 M^{-1}. Source data are provided as a Source Data file.

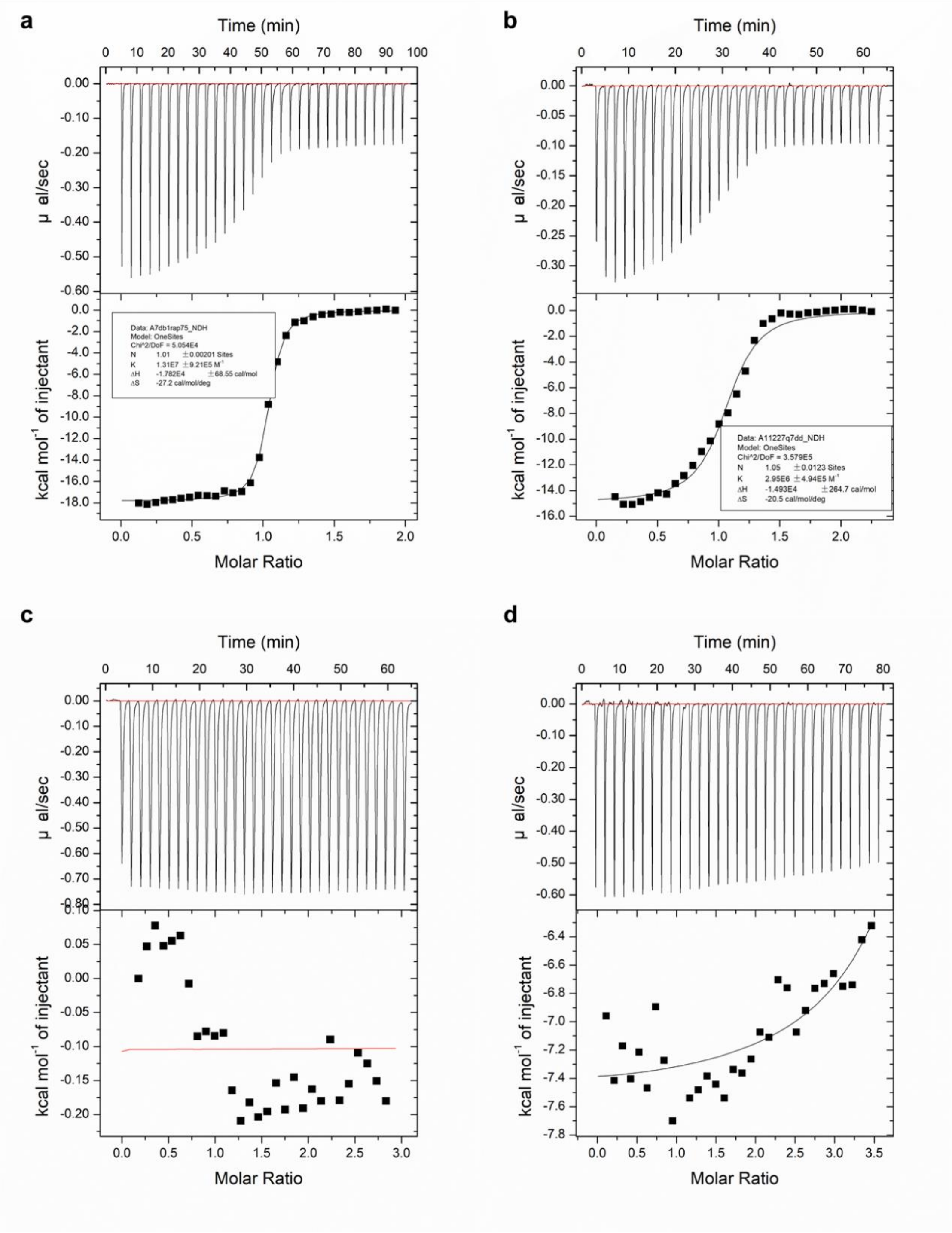

Supplementary Fig. 2. ITC analysis of CB[7] binding affinities on the cytosine-modified ODNs. (**a**) Binding with the AD-containing ODN; calculated K_a = 1.31 × 10^7 M^{-1}. (**b**) Binding with the FC-containing ODN; calculated K_a = 2.95 × 10^6 M^{-1}. (**c**) Binding with the BA-containing ODN. (**d**) Binding with the TB-containing ODN. The ITC signals of CB[7] binding on the BA and TB modified ODNs were too weak to determine effective K_a values, suggesting greatly lower binding affinities than the other two (presumably below 10^4 M^{-1} based on our experimental conditions). The 15-nt ODN

with a guest-integrated cytidine as described in Supplementary Table 1 was selected in this experiment. The binding affinities with these guest-containing ODNs were much weaker than the free guest molecules in cation forms, potentially attributed to the unprotonated amine group and steric hindrance by the nucleobase. Source data are provided as a Source Data file.

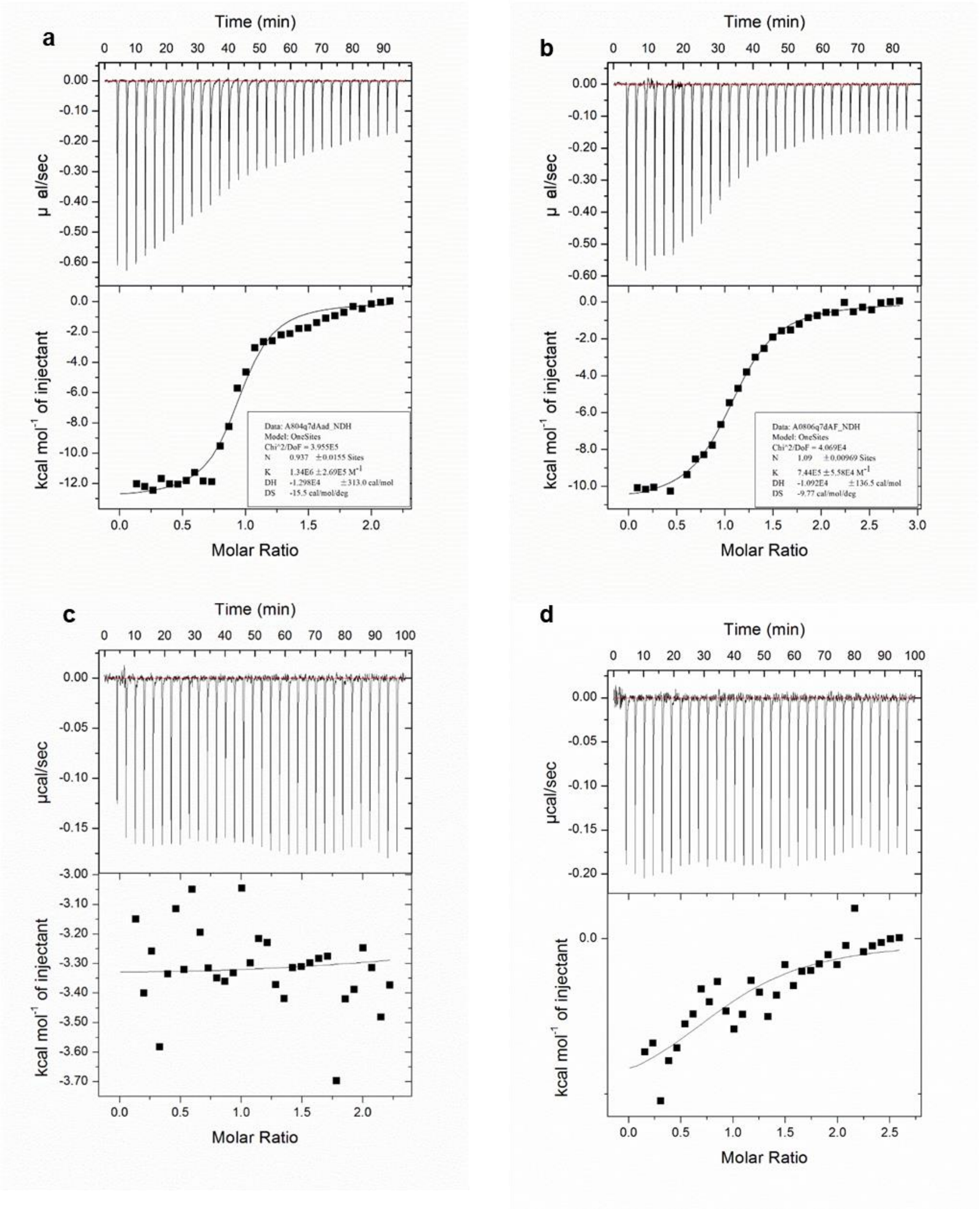

Supplementary Fig. 3. ITC analysis of CB[7] binding affinities on the adenine-modified ODNs. (**a**) Binding with the AD-containing ODN; calculated $K_a = 1.34 \times 10^6$ M^{-1}. (**b**) Binding with the FC-containing ODN; calculated $K_a = 7.44 \times 10^5$

M^{-1}. (**c**) Binding with the BA-containing ODN. (**d**) Binding with the TB-containing ODN. The 15-nt ODN with a guest-integrated cytidine as described in Supplementary Table 1 was selected in this experiment. Notably, the binding affinities of CB[7] on the modified adenine are relatively weaker than the modified cytosine, which may be attributed to different steric hindrance by the nucleobase. Source data are provided as a Source Data file.

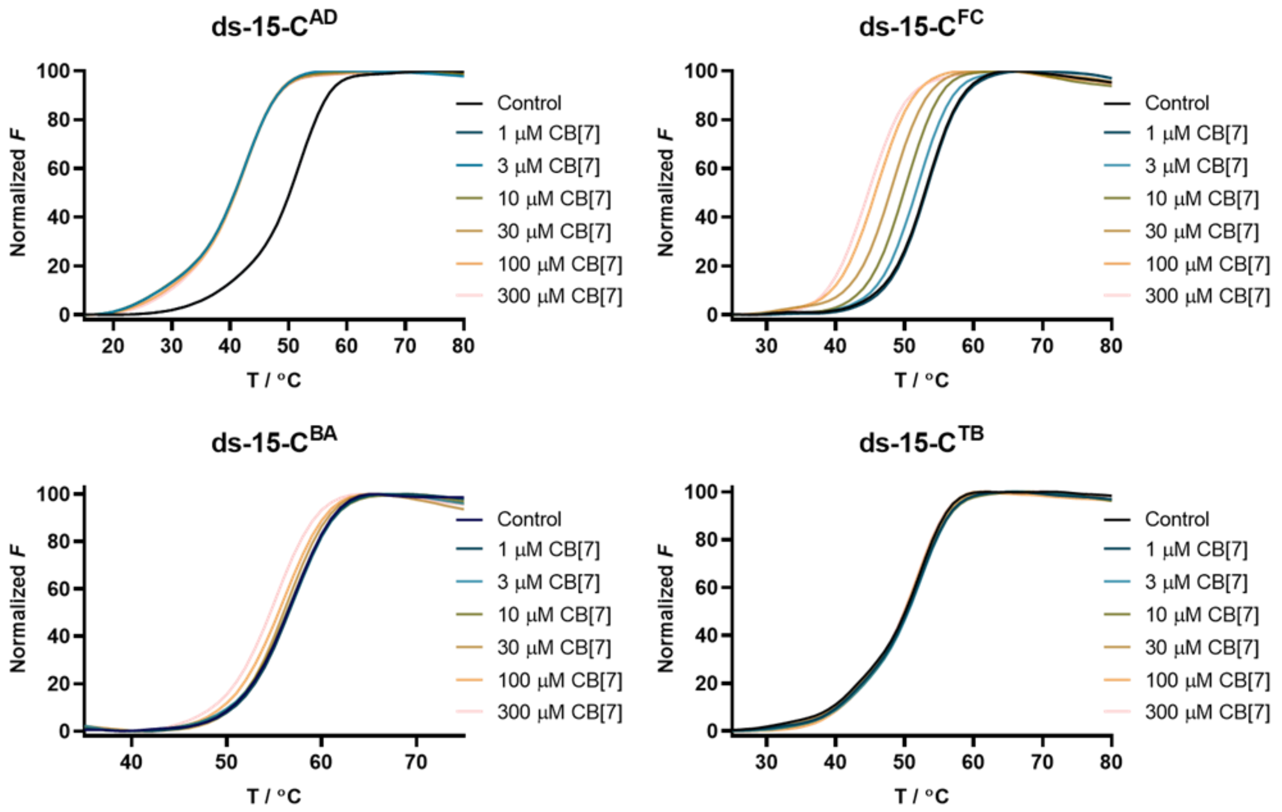

Supplementary Fig. 4. Representative melting curves of the 15mer dsDNA with a single site of guest-integrated cytidine in the presence of different concentrations of CB[7]. The sequence information was listed in Supplementary Table 1. The guest-containing duplex without treatment of CB[7] was selected as control. Source data are provided as a Source Data file.

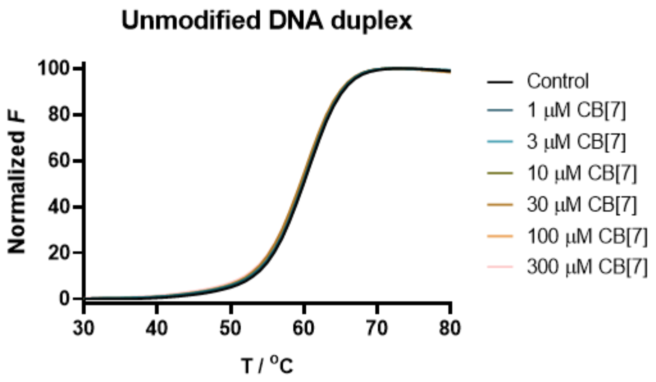

Supplementary Fig. 5. Representative melting curves of the unmodified 15mer dsDNA with treatment of different concentrations of CB[7]. The sequence information was listed in Supplementary Table 1. The T_m value for unmodified dsDNA was 59.7 °C and treatment of CB[7] did not influence the thermostabilities of unmodified dsDNA. Notably, compared with the unmodified duplex DNA, the guest-containing ODNs exhibited relatively lowered thermostabilities. This was fully expectable due to modifications at the amino group of nucleobase,[3,4] but not an issue for dynamic manipulation of DNA duplex structures, as modified ODNs can still tightly bind with the complementary strand. Source data are provided as a Source Data file.

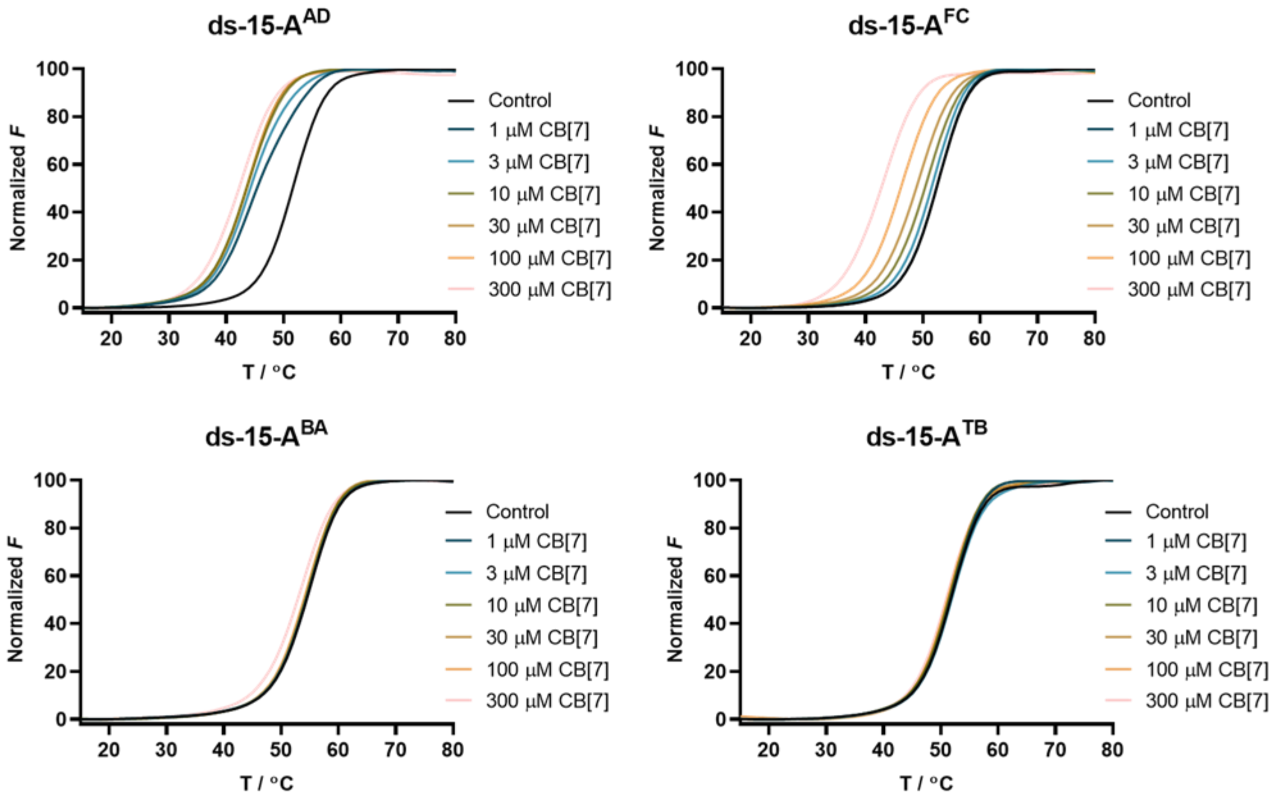

Supplementary Fig. 6. Representative melting curves of the 15mer dsDNA with a single site of guest-integrated adenine

in the presence of different concentrations of CB[7]. The guest-containing duplex without treatment of CB[7] was selected as control. The sequence information was listed in Supplementary Table 1. Source data are provided as a Source Data file.

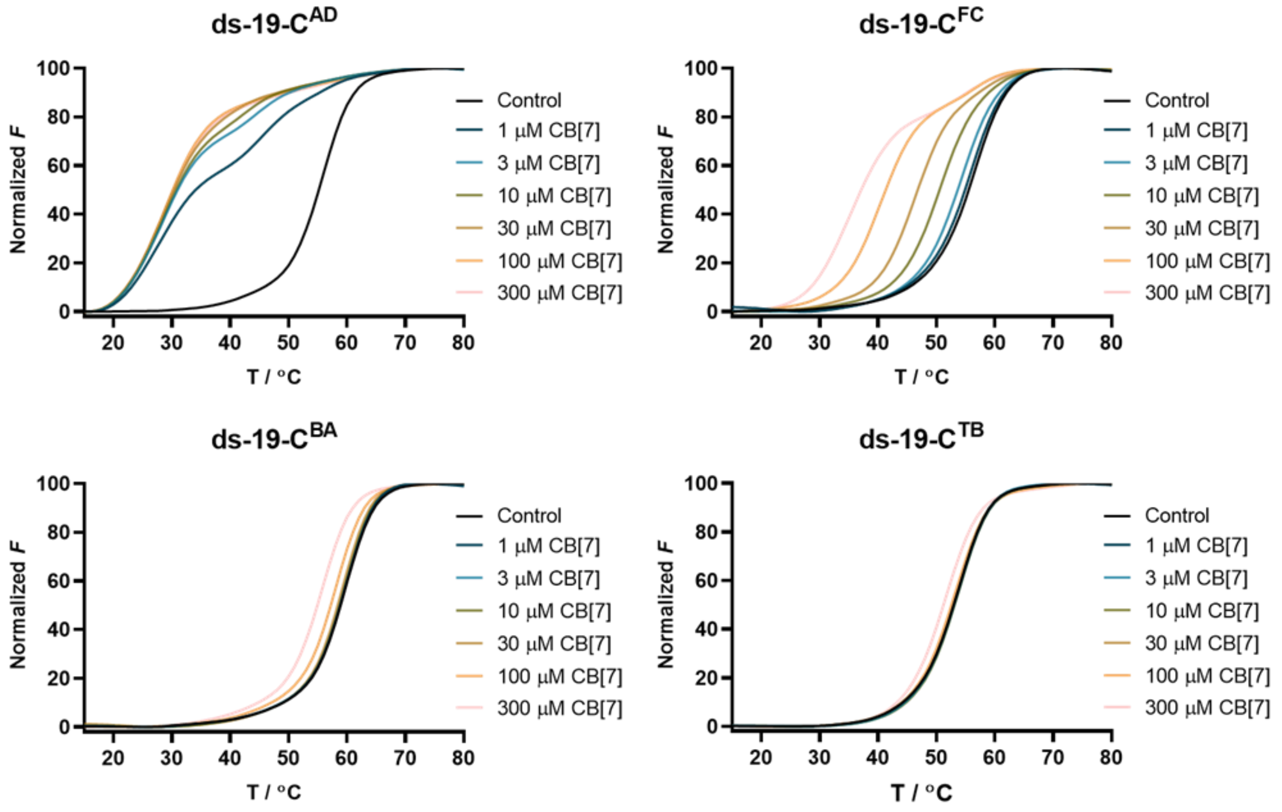

Supplementary Fig. 7. Representative melting curves of the 19mer dsDNA with two sites of guest-integrated cytidines in the presence of different concentrations of CB[7]. The variable slopes of melting curves observed in the AD- and FC-containing duplex might reveal the incomplete recognition of two modified sites during the treatment of CB[7]. The guest-containing duplex without treatment of CB[7] was selected as control. The sequence information was listed in Supplementary Table 1. Source data are provided as a Source Data file.

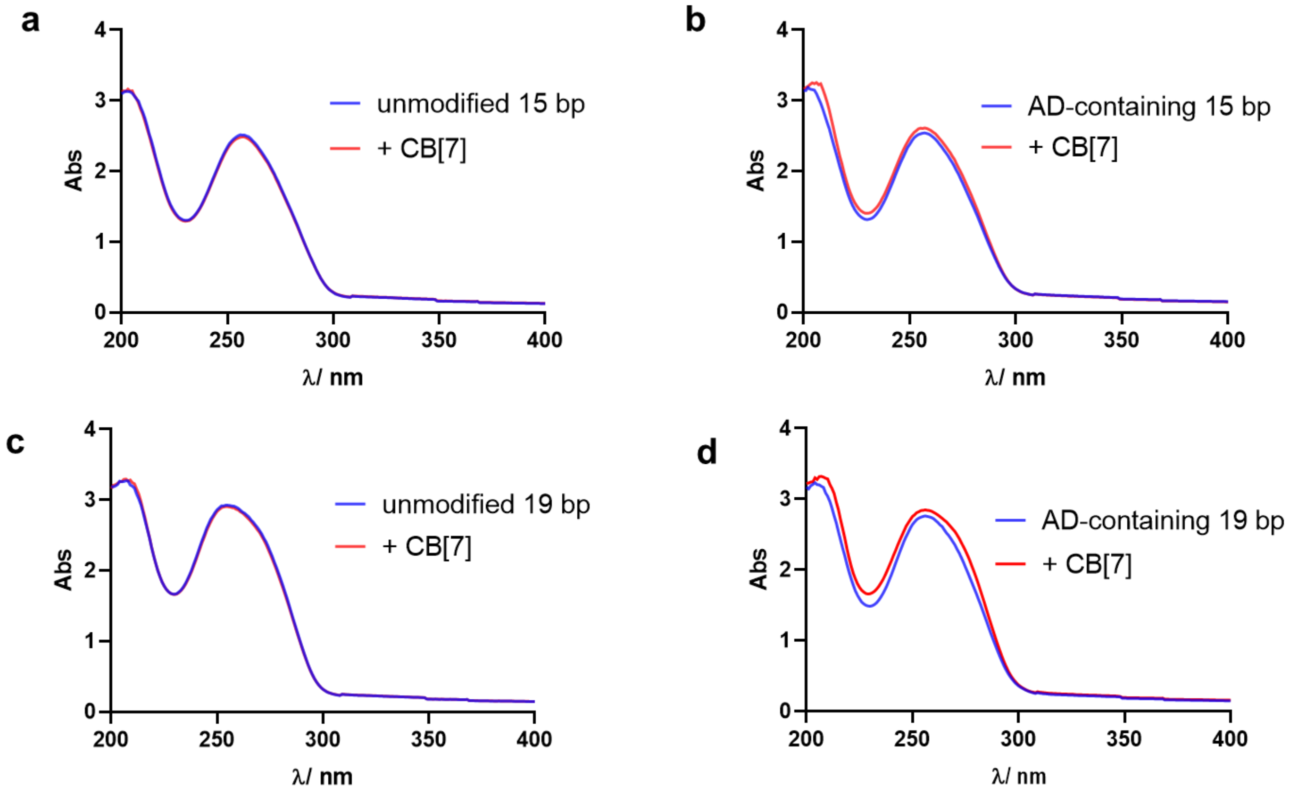

Supplementary Fig. 8. UV spectra of the 15mer and 19mer dsDNA treated with 500 μM CB[7]. Treatment of CB[7] does not influence UV absorbance of unmodified dsDNA (**a** and **c**). The UV absorption of AD-containing dsDNA was increased upon the CB[7] treatment, and the absorption change of 19-bp DNA (**d**) with two AD-modified cytosine sites was greater than the 15-bp DNA (**b**) with one AD-modified cytosine site. The sequence information was listed in Supplementary Table 1. Source data are provided as a Source Data file.

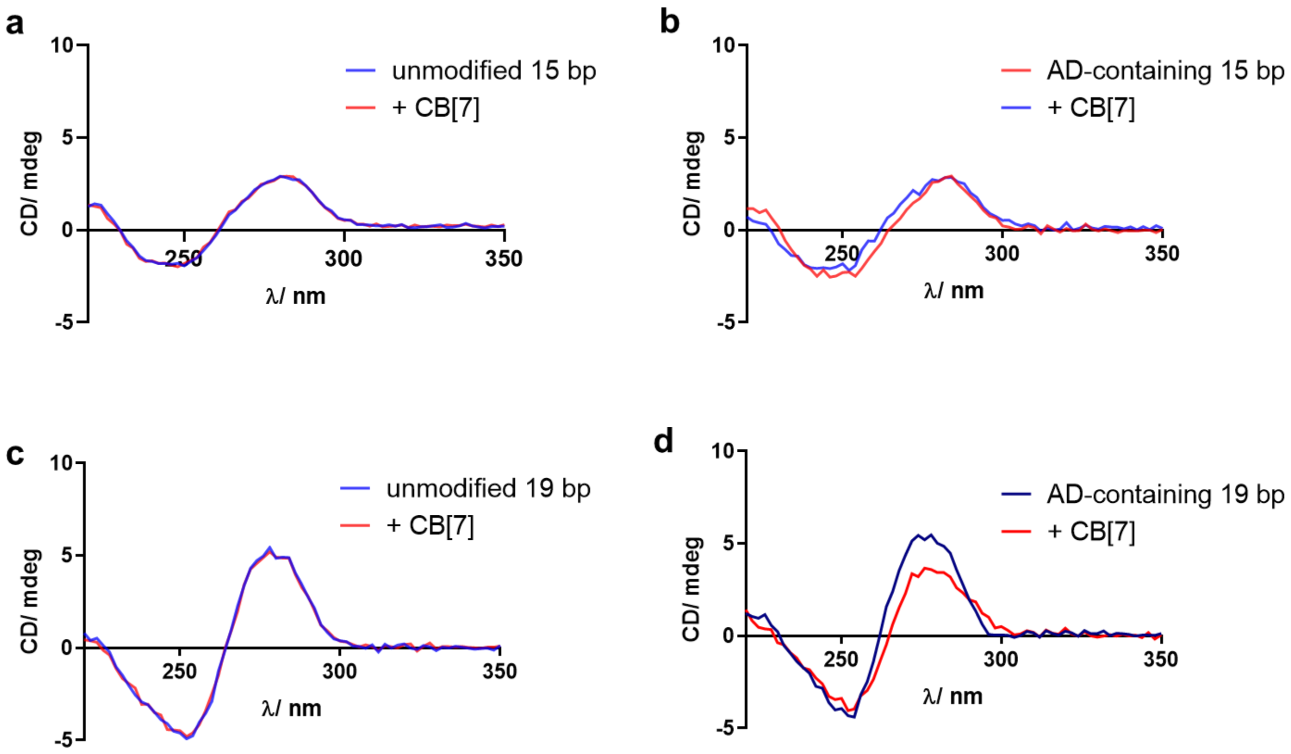

Supplementary Fig. 9. CD spectra of the 15mer and 19mer dsDNA treated with 500 μM CB[7]. Treatment of CB[7] does not influence CD spectra of unmodified dsDNA (**a** and **c**). The CD spectra of the AD-containing dsDNA was increased upon the CB[7] treatment, and the change of 19-bp DNA (**d**) with two AD-modified cytosine sites was greater than the 15-bp DNA (**b**) with one AD-modified cytosine site. The sequence information was listed in Supplementary Table 1. Source data are provided as a Source Data file.

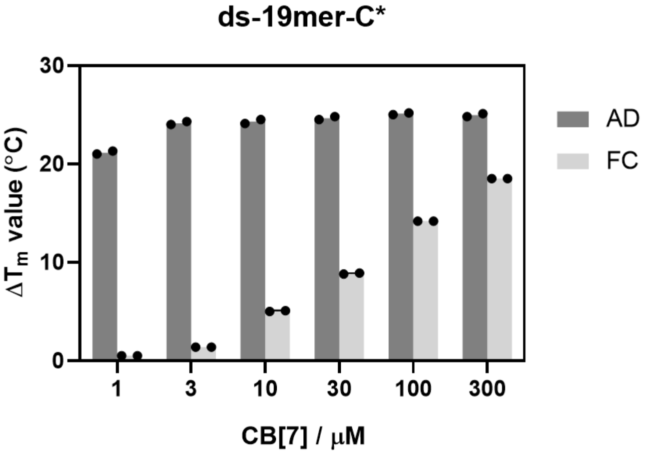

Supplementary Fig. 10. Comparison of concentration-dependent ΔT_m values. The 19-mer dsDNA with two modified cytidine sites as described in Supplementary Table 1 were analyzed in this figure. "AD" and "FC" indicated the AD or FC-integrated cytidine as the recognition handle for CB[7], respectively. Clearly, a low concentration of CB[7] was enough to induce a maximum disruption for the AD-containing strand, whereases a high concentration was needed to generate intense disruption towards the FC-modified duplex structure. Bar graphs showed the mean data derived from two independent replicates. Source data are provided as a Source Data file.

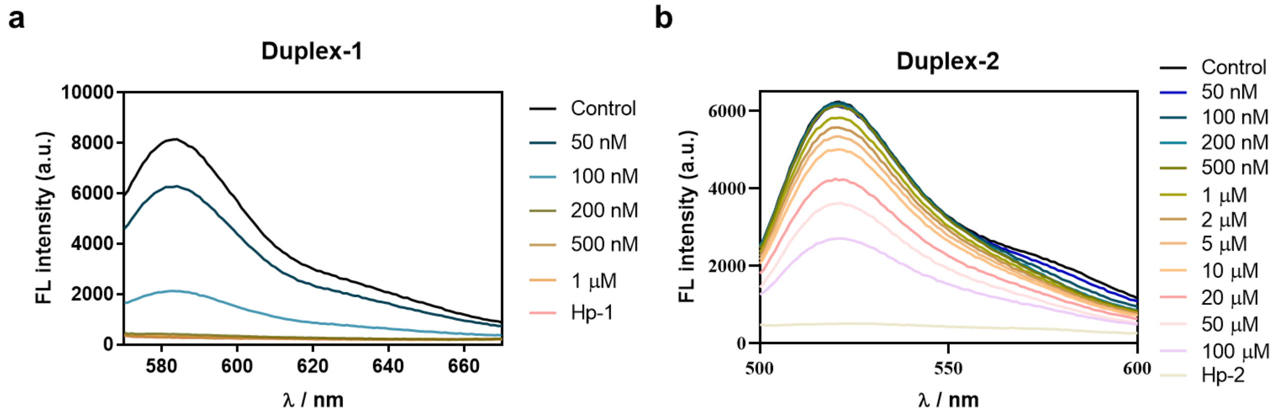

Supplementary Fig. 11. Representative fluorescence spectra of Duplex-1 (**a**) and Duplex-2 (**b**) with treatments of different concentrations of CB[7]. Control: the duplex state without treatment of CB[7]; Hp-1: the hairpin structure released from Duplex-1; Hp-2: the hairpin structure released from Duplex-2. Duplex-1 and Duplex-2 co-existed in the same system. The fluorescence signals of FAM were recorded as indications for conversion of Duplex-2; The fluorescence signals of TMR were recorded as indications for conversion of Duplex-1. Duplex-1 was firstly converted into the hairpin form during the titration of CB[7]; even when Duplex-1 was totally dissociated, Duplex-2 was still stably maintained in the same system; with further increase of the CB[7] concentration, Duplex-2 was then destructed to generate the hairpin product. The

sequence information was listed in Supplementary Table 1. Source data are provided as a Source Data file.

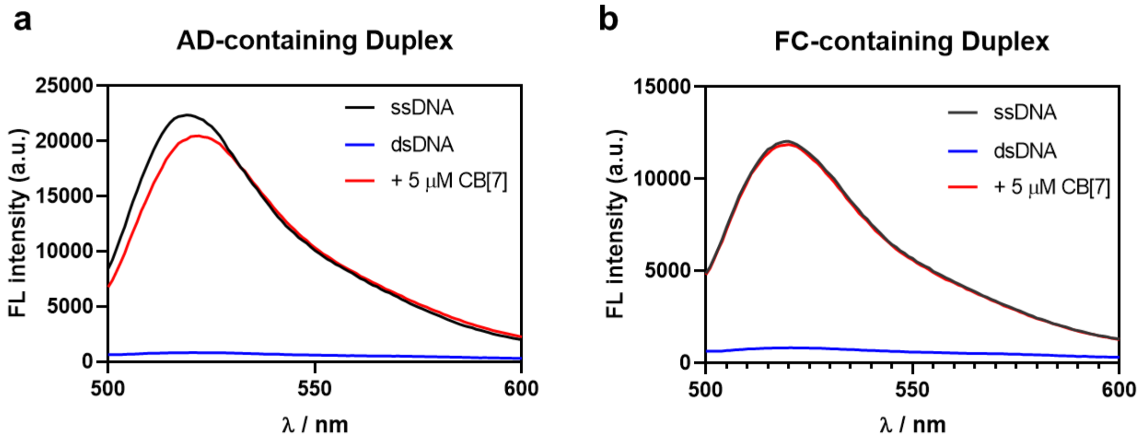

Supplementary Fig. 12. Representative fluorescence spectra of CB[7] induced dissociation of DNA duplex with AD-modified sites (**a**) or with FC-modified sites (**b**) at the room temperature. A 15-mer modified dsDNA formed by a fluorophore-labeled ODN with either three AD or FC-modified cytosine sites and the quencher-labeled complementary strand was investigated in this experiment. ssDNA: fluorophore-labelled single-stranded DNA as an indication of totally dissociated DNA duplex. Fluorescence was quenched in dsDNA; with treatment of 5 μM CB[7], fluorescence signals for both AD- and FC-containing duplex were all fully recovered, demonstrating the formation of totally separated DNA strands. In fact, with accumulated destabilization effects through introduction of multiple recognition handles, the DNA duplex could be directly dissociated by CB[7] at a desired temperature with a rationally designed length and sequence. The sequence information was listed in Supplementary Table 1. Source data are provided as a Source Data file.

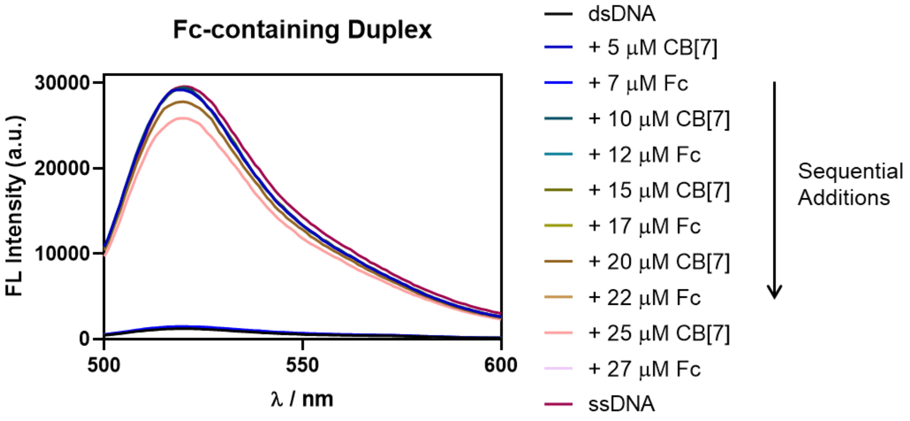

Supplementary Fig. 13. Representative fluorescence spectra of the FC-containing duplex with sequential additions of CB[7] and the competing guest molecule FC. The conversion rates were determined by normalization using the fluorescence of dsDNA as 0 and the fluorescence of ssDNA as 1. Source data are provided as a Source Data file.

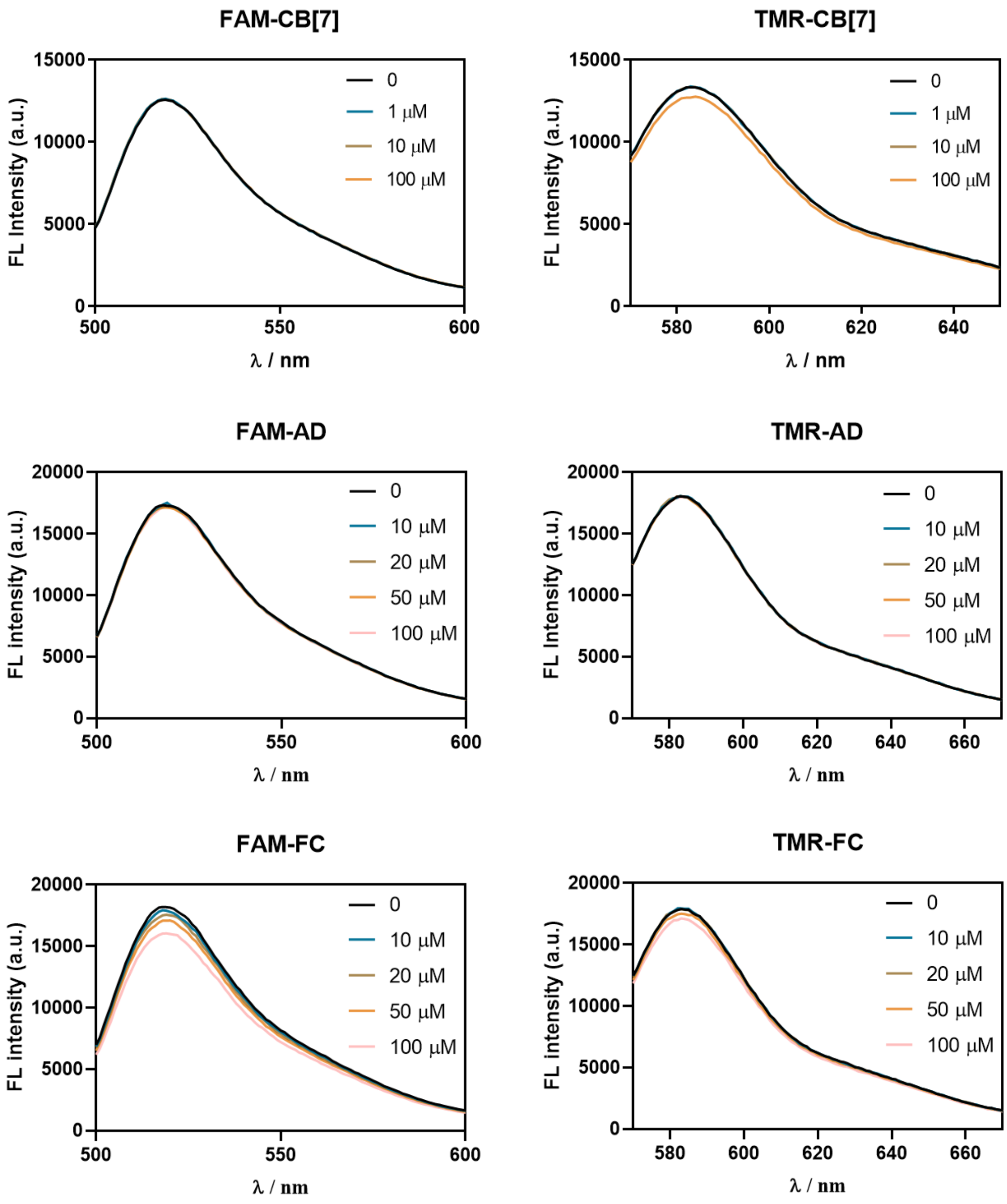

Supplementary Fig. 14. Influence of these compounds (CB[7], AD and FC) on fluorescence of the FAM and TMR fluorophores. In our conditions, all of the compounds exhibited limited impacts on the fluorescence signals of FAM and TMR when their concentrations were below 100 μM. Source data are provided as a Source Data file.

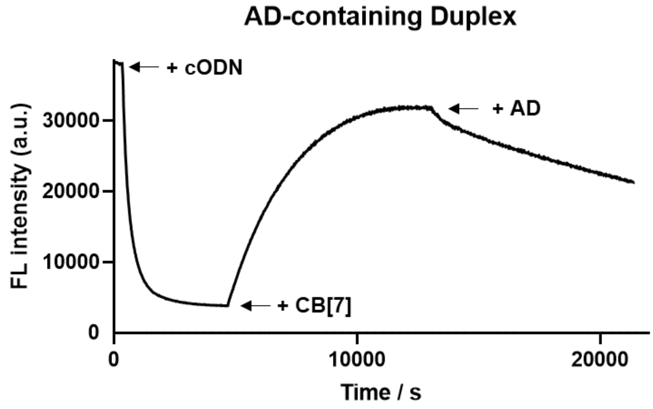

Supplementary Fig. 15. Kinetically monitoring of DNA duplex dissociation and formation with the integrated AD group as recognition handles. A 15mer dsDNA with three guest-modified sites as described in Supplementary Table 1 was investigated in this experiment. The AD-containing strand was labelled by FAM and the complementary strand (cODN) was labelled by BHQ-1. Compared with the FC-containing duplex, utilization of AD as the recognition handle exhibited a much lower kinetic process for both invasion and dissociation. In the same duplex with the same modified sites, k_{app} for CB[7] invasion through the AD group was 42 $M^{-1}\cdot s^{-1}$, which was about 50-fold lower than the FC group (2.0×10^3 $M^{-1}\cdot s^{-1}$). Source data are provided as a Source Data file

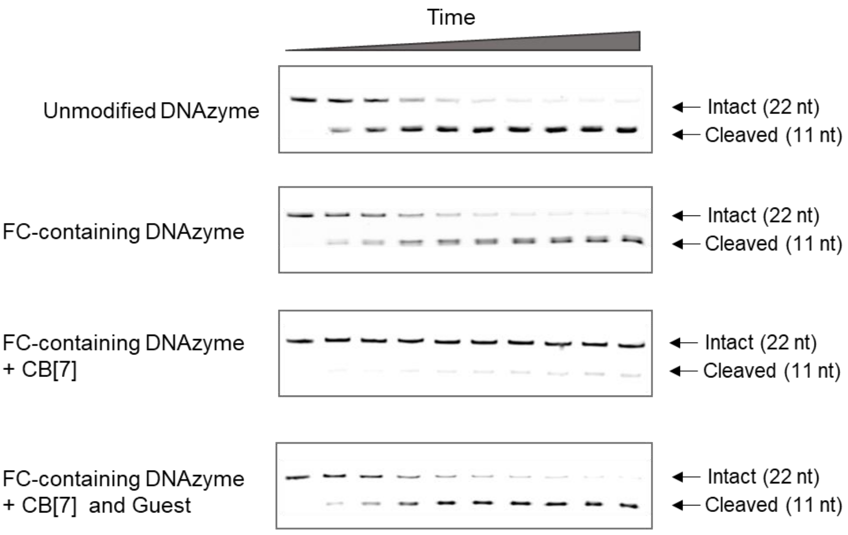

Supplementary Fig. 16. Representative gel analysis of the FC-modified DNAzyme cleavage activities under different conditions. Time points were 0, 4, 8, 16, 32, 40, 48, 56 and 64 min, respectively. The concentration of CB[7] was 300 μM and the competing guest FC was 500 μM. This experiment was repeated three times independently with similar results.

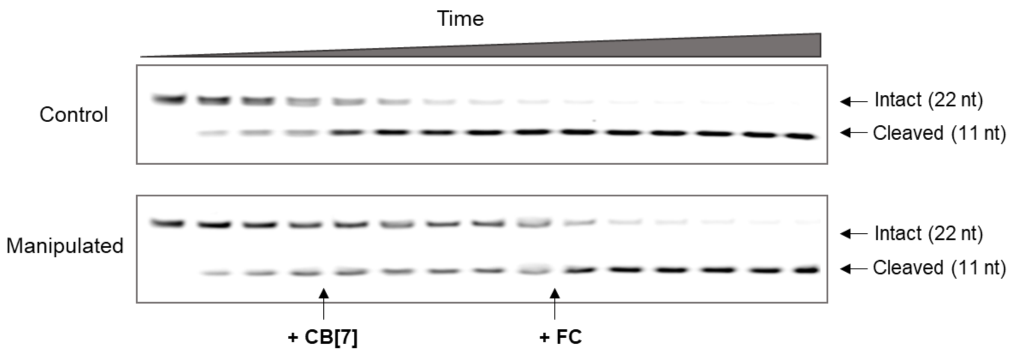

Supplementary Fig. 17. Representative gel analysis of the FC-modified DNAzyme cleavage activities under different conditions. Time points were 0, 3, 6, 9, 15, 21, 27, 33, 39, 51, 63, 75, 87, 99 and 111 min, respectively. The manipulated DNAzyme system was firstly treated with CB[7] (300 μM) at the 9-min point, and then mixed with the FC guest (500 μM) at the 39-min point. This experiment was repeated three times independently with similar results.

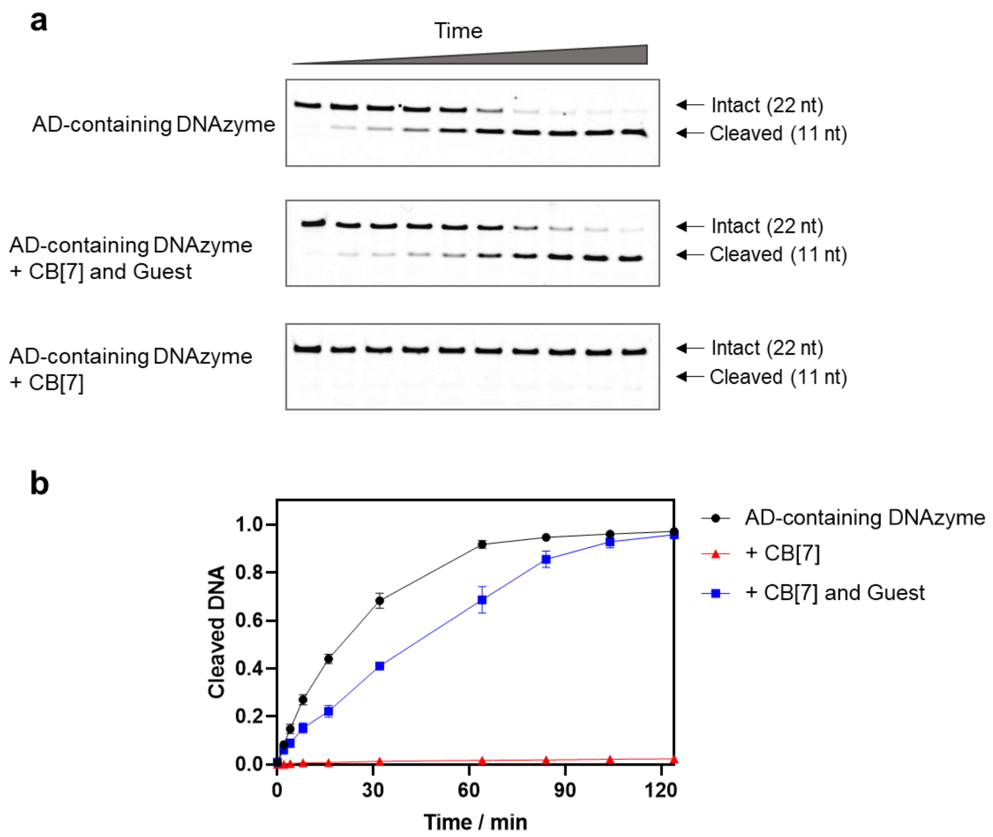

Supplementary Fig. 18. Deactivation and Reactivation of the AD-modified DNAzyme through the invasion of CB[7]. (**a**) Representative gel analysis of DNAzyme cleavage activities under different conditions. Time points were 0, 2, 4, 8, 16, 32, 64, 84, 104 and 124 min, respectively. This experiment was repeated three times independently with similar results. (**b**) Quantitative analysis of cleavage products at different time points. The AD-containing DNAzyme presented a strong

cleavage activities, whereas the treatment of CB[7] (10 μM) abolished its function. Incubation with the competing guest AD (500 μM) recovered its cleavage activity. Data are presented as mean values with standard deviations derived from three independent replicates. Source data are provided as a Source Data file.

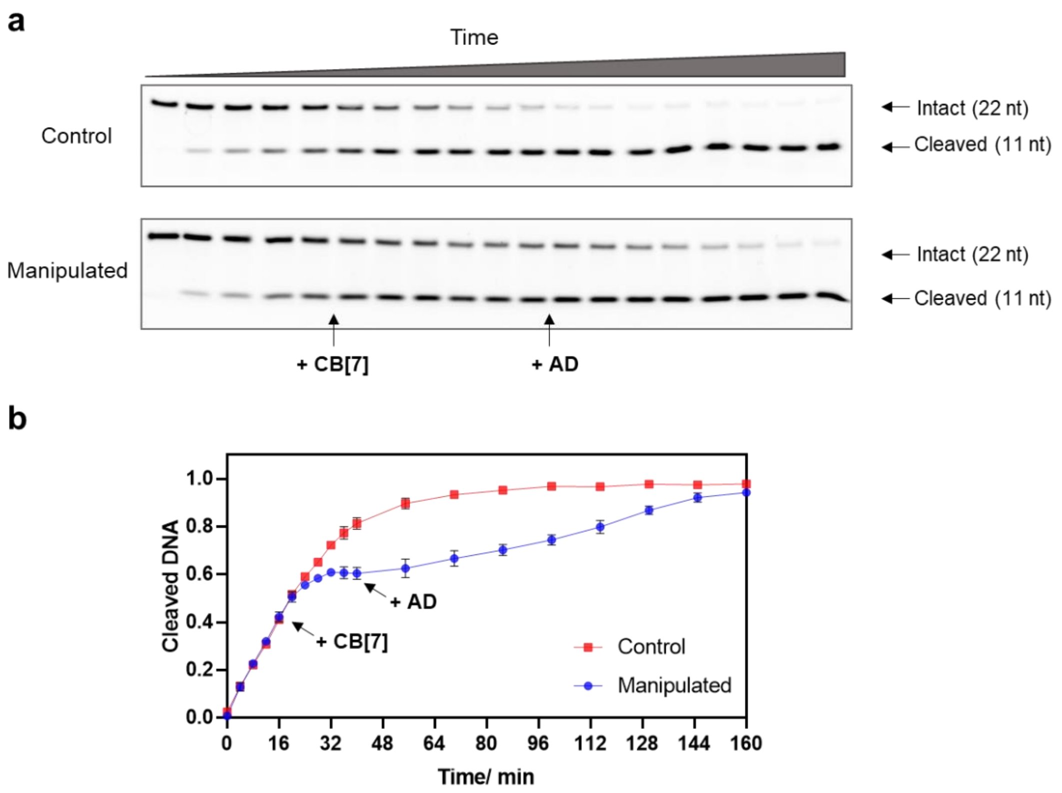

Supplementary Fig. 19. Deactivation and reactivation of DNAzyme activities through the host-guest interaction. (**a**) Representative gel analysis of DNAzyme cleavage activities under different conditions. Time points were 0, 4, 8, 12, 16, 20, 24, 28, 32, 36, 40, 55, 70, 85, 100, 115, 130, 145 and 160 min, respectively. This experiment was repeated three times independently with similar results. (**b**) Quantitative analysis of cleavage products at different time points. The AD-containing DNAzyme system without treatment of CB[7] or AD was selected as control. The manipulated DNAzyme system was firstly treated with CB[7] (10 μM) at the 16-min point, and then mixed with the AD guest (500 μM) at the 40-min point. Data are presented as mean values with standard deviations derived from three independent replicates. Source data are provided as a Source Data file.

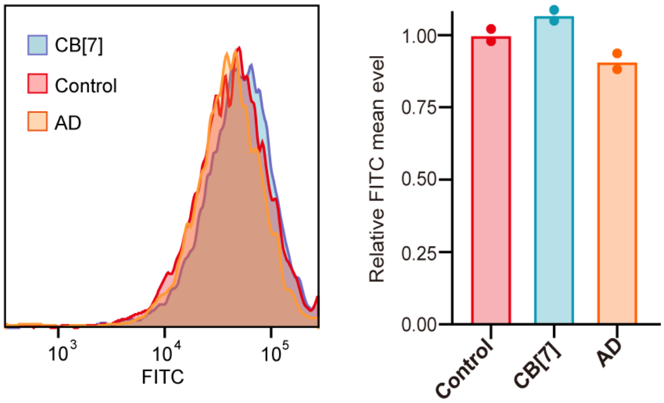

Supplementary Fig. 20. Effects of CB[7] and the free AD guest on expressions of dsGFP in HEK293T cells. The untreated cells were selected as control; 200 µM CB[7] or 100 µM AD were added during cell culture. Cells were cultured for two days before measurement of fluorescence. The left panel showed the flow cytometry profiles, and the right panel showed the quantitative analysis of average fluorescence normalized to the control samples. Bar graphs showed the mean data derived from two independent replicates. Source data are provided as a Source Data file.

Supplementary Figures (NMR spectra of guest-containing phosphoramidites)

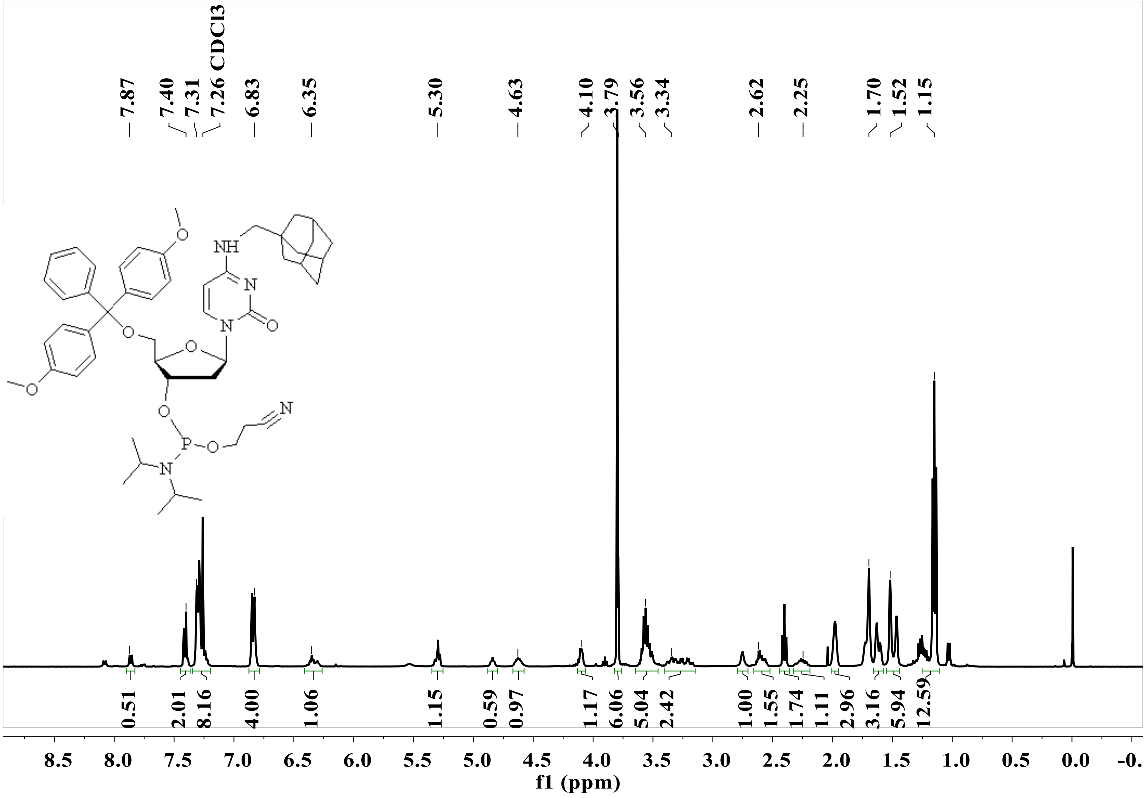

Supplementary Fig. 21. ^{1}H NMR (400 MHz, $CDCl_3$) of compound A6^{AD}.

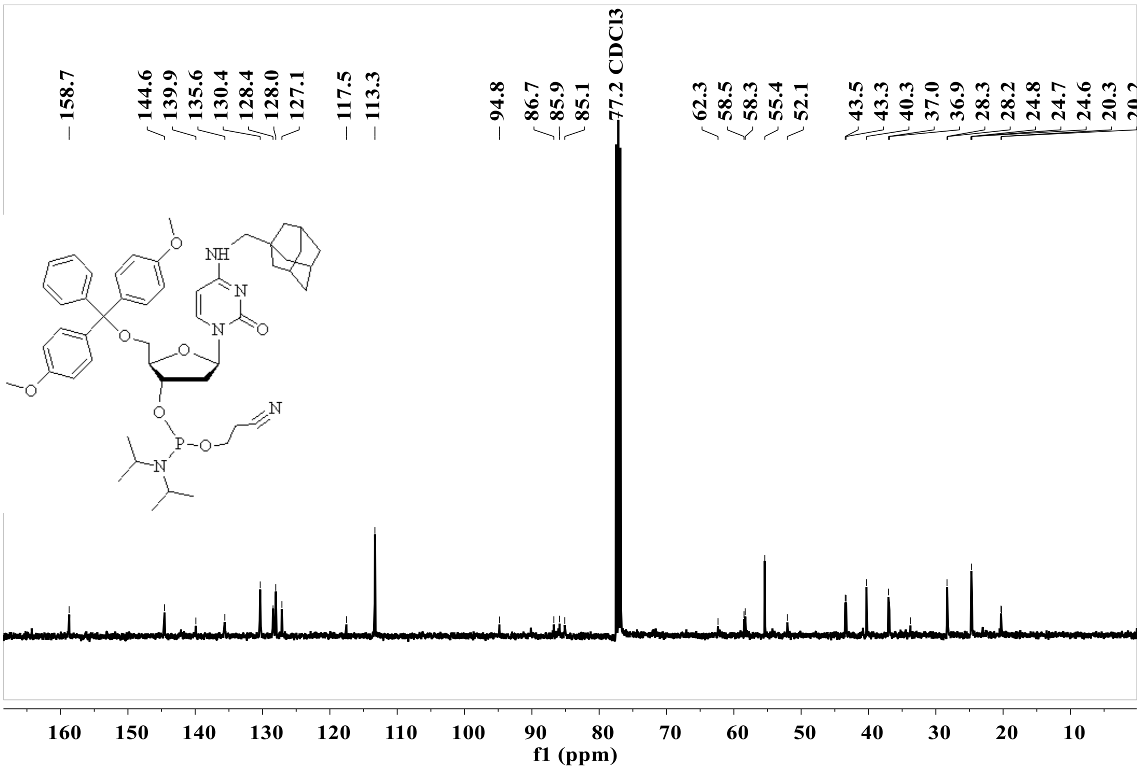

Supplementary Fig. 22. ^{13}C NMR (400 MHz, $CDCl_3$) of compound A6^{AD}.

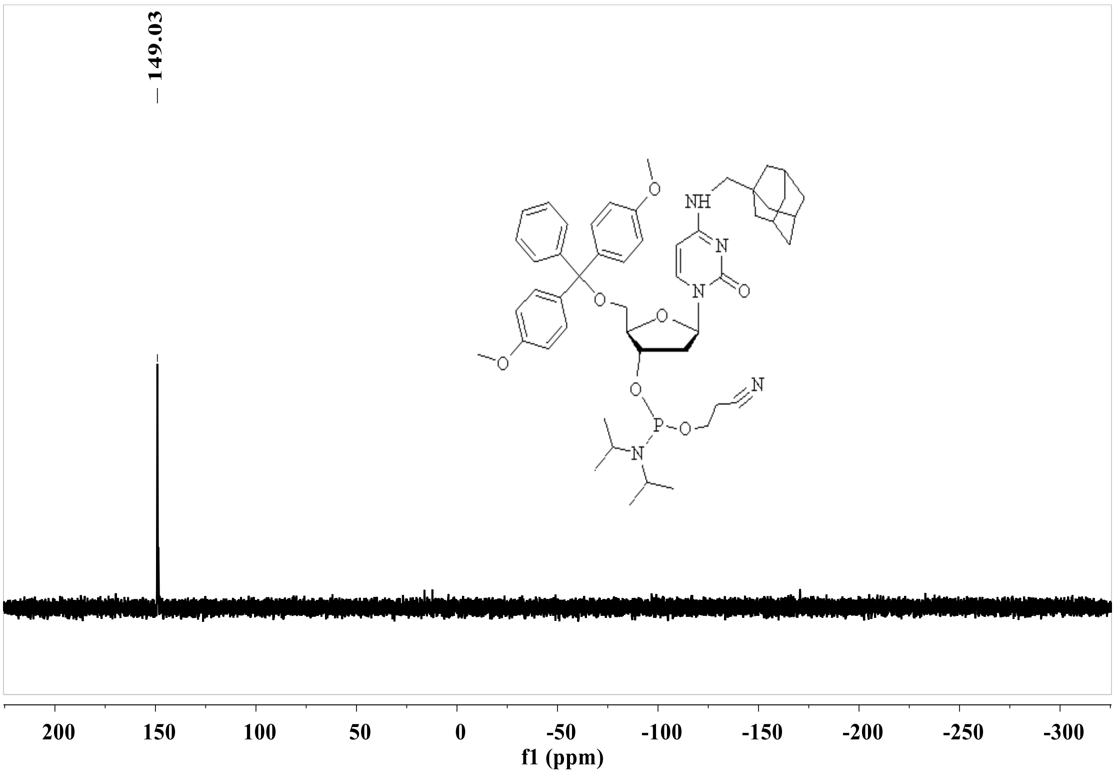

Supplementary Fig. 23. ^{31}P NMR (400 MHz, $CDCl_3$) of compound A6^{AD}.

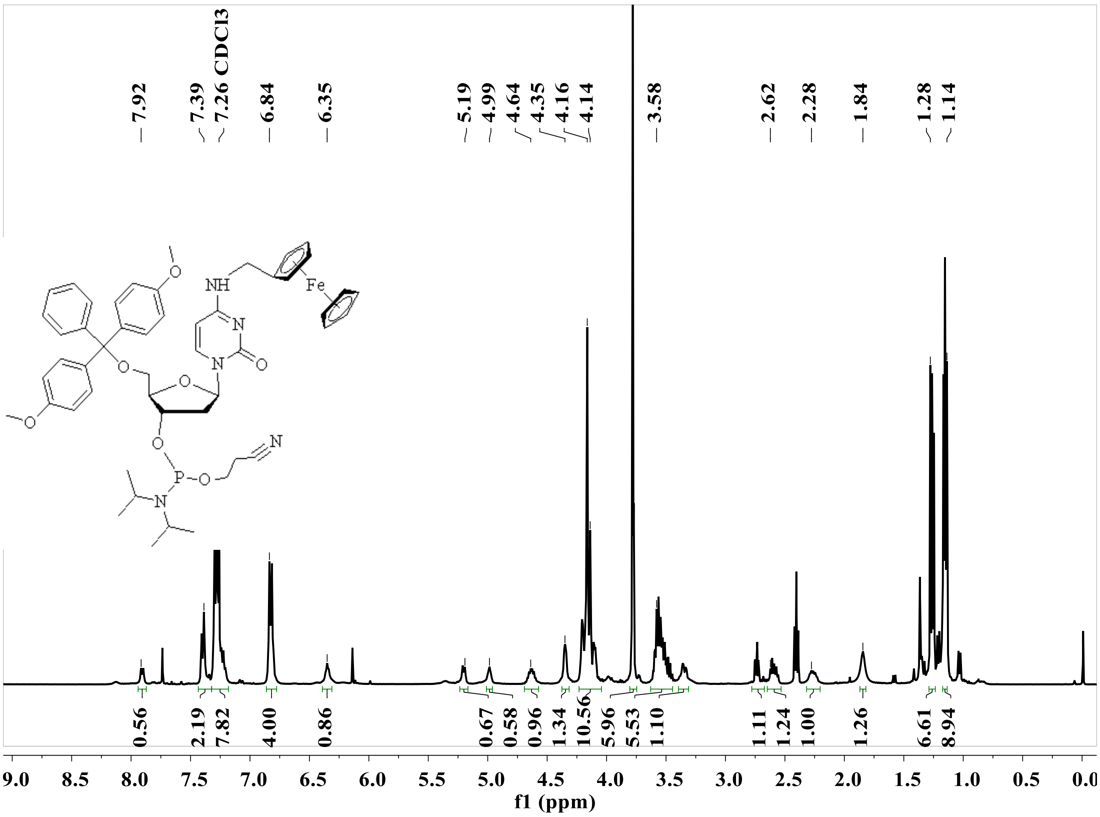

Supplementary Fig. 24. ^{1}H NMR (400 MHz, $CDCl_3$) of compound A6^{FC}.

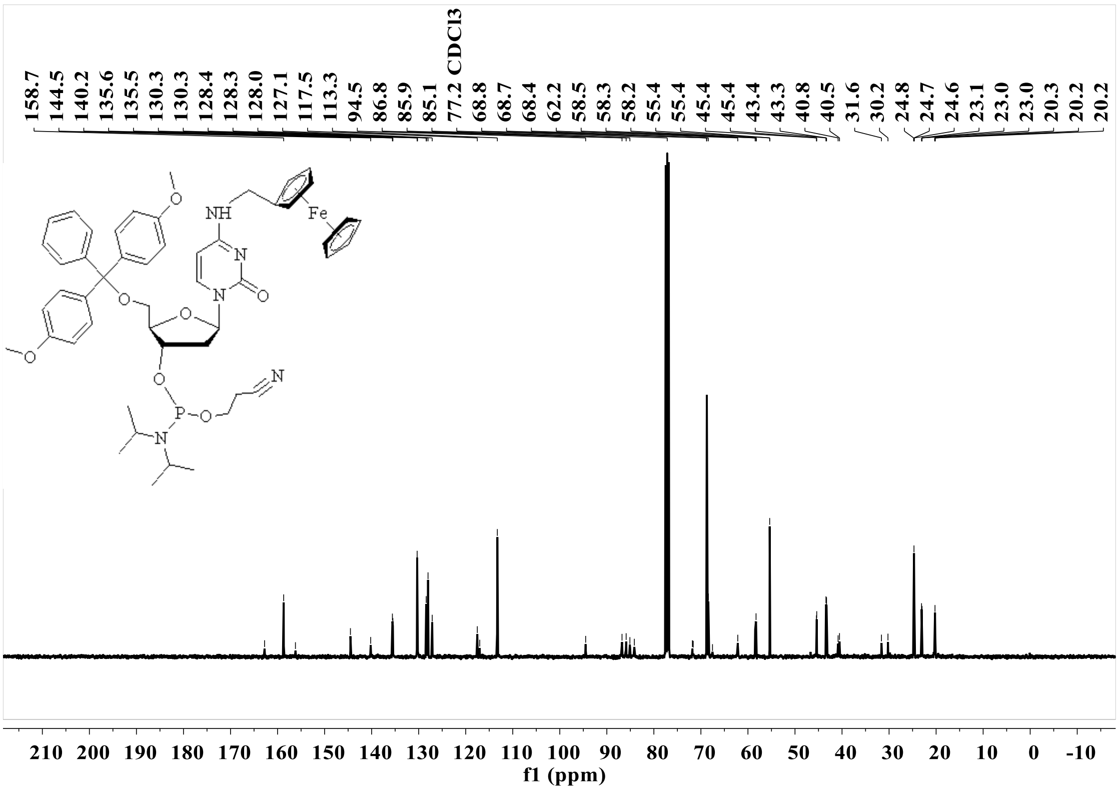

Supplementary Fig. 25. ^{13}C NMR (400 MHz, $CDCl_3$) of compound A6[FC].

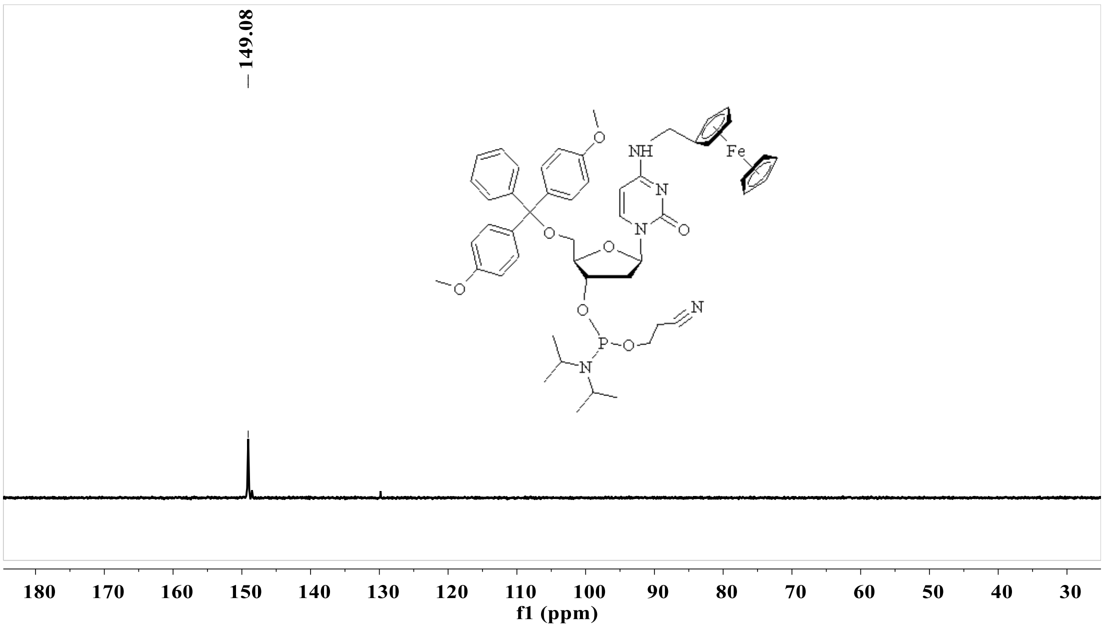

Supplementary Fig. 26. ^{31}P NMR (400 MHz, $CDCl_3$) of compound A6[FC].

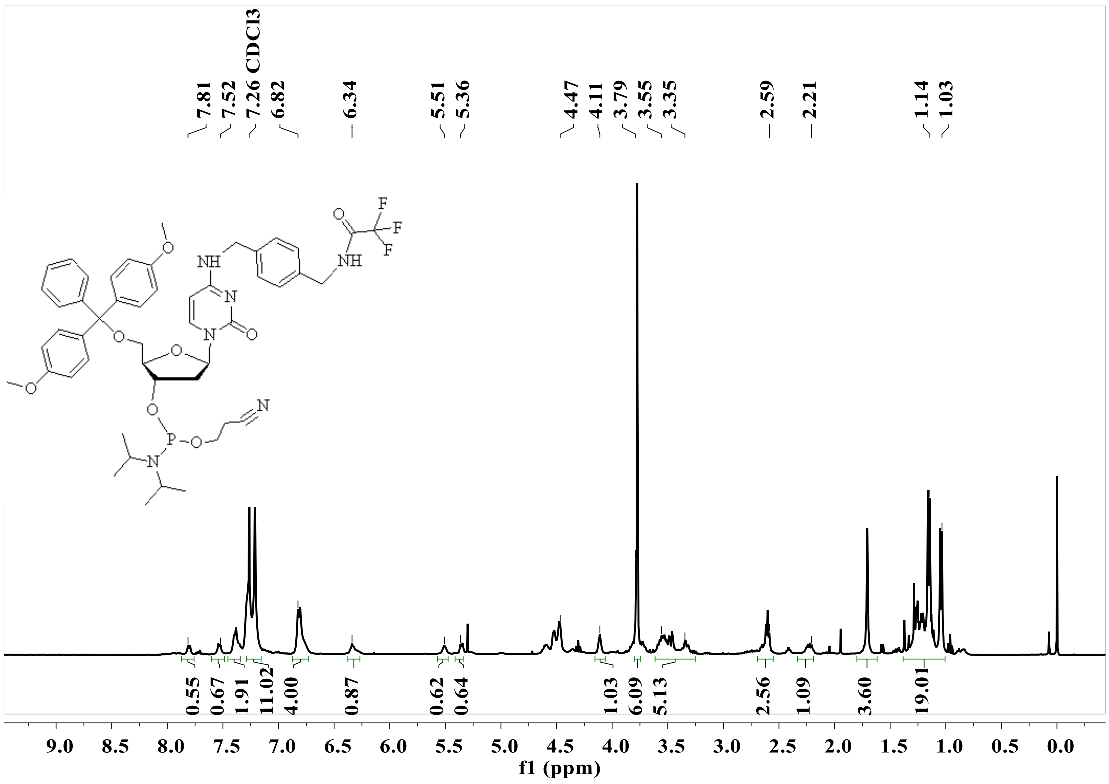

Supplementary Fig. 27. ^{1}H NMR (400 MHz, $CDCl_3$) of compound A6[BA].

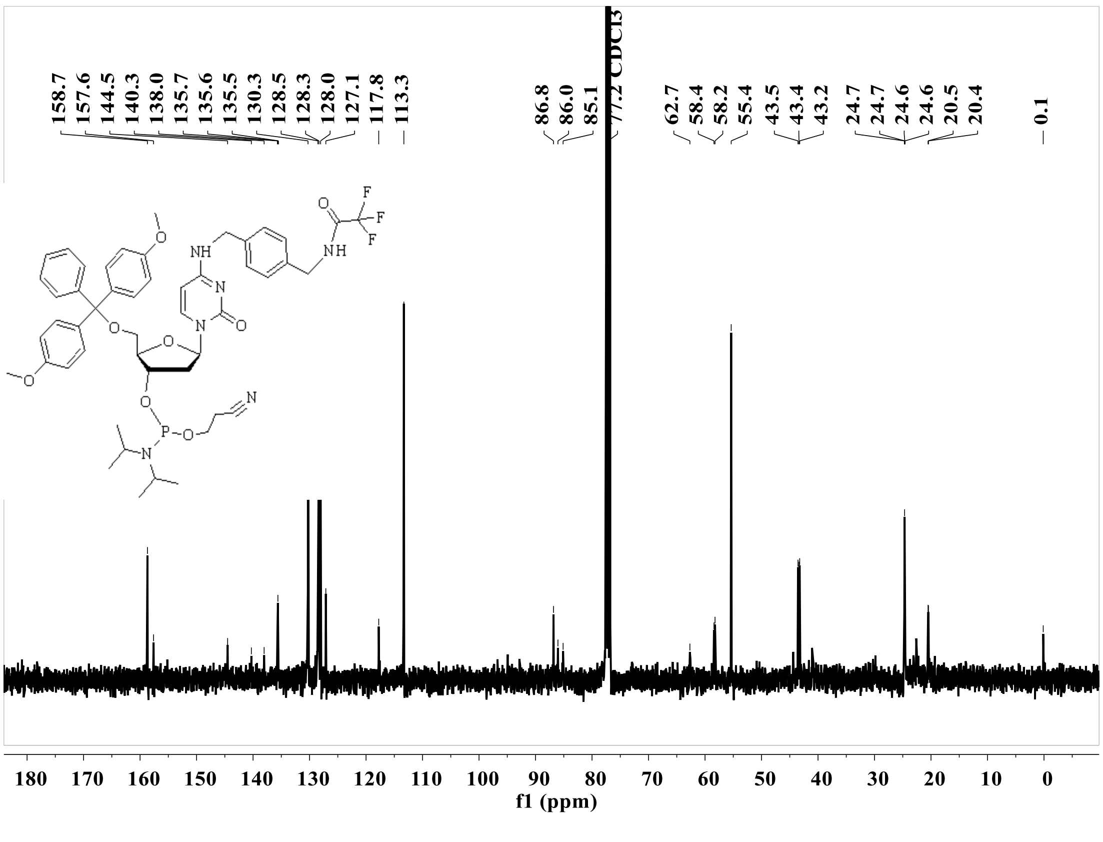

Supplementary Fig. 28. ^{13}C NMR (400 MHz, $CDCl_3$) of compound A6[BA].

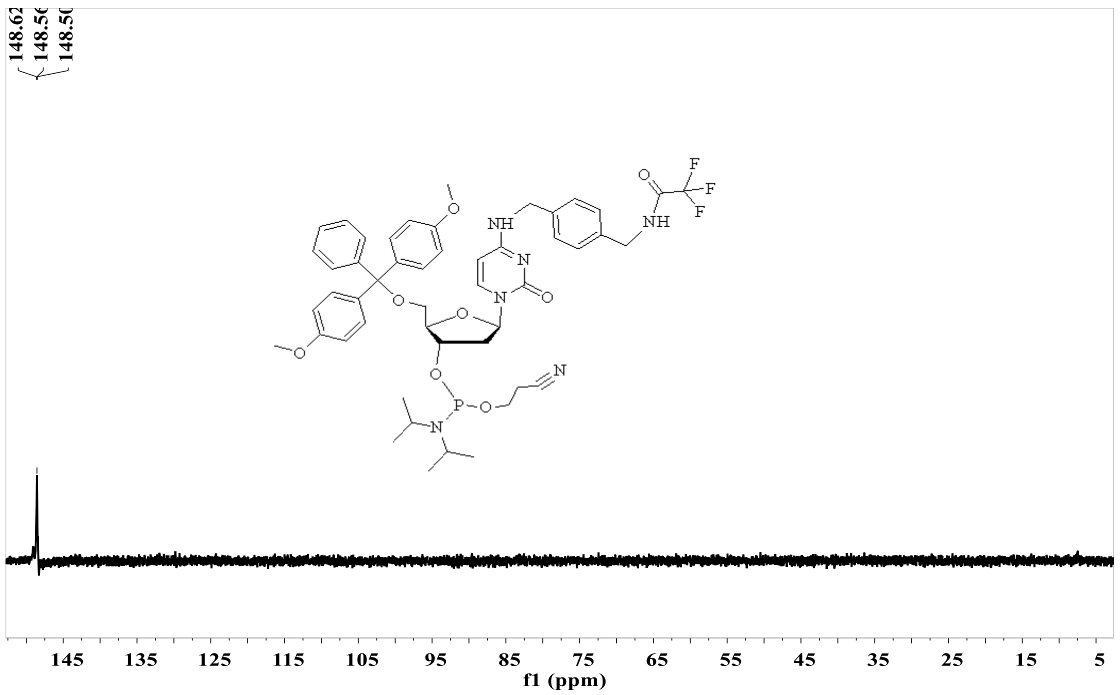

Supplementary Fig. 29. ^{31}P NMR (400 MHz, $CDCl_3$) of compound A6[BA].

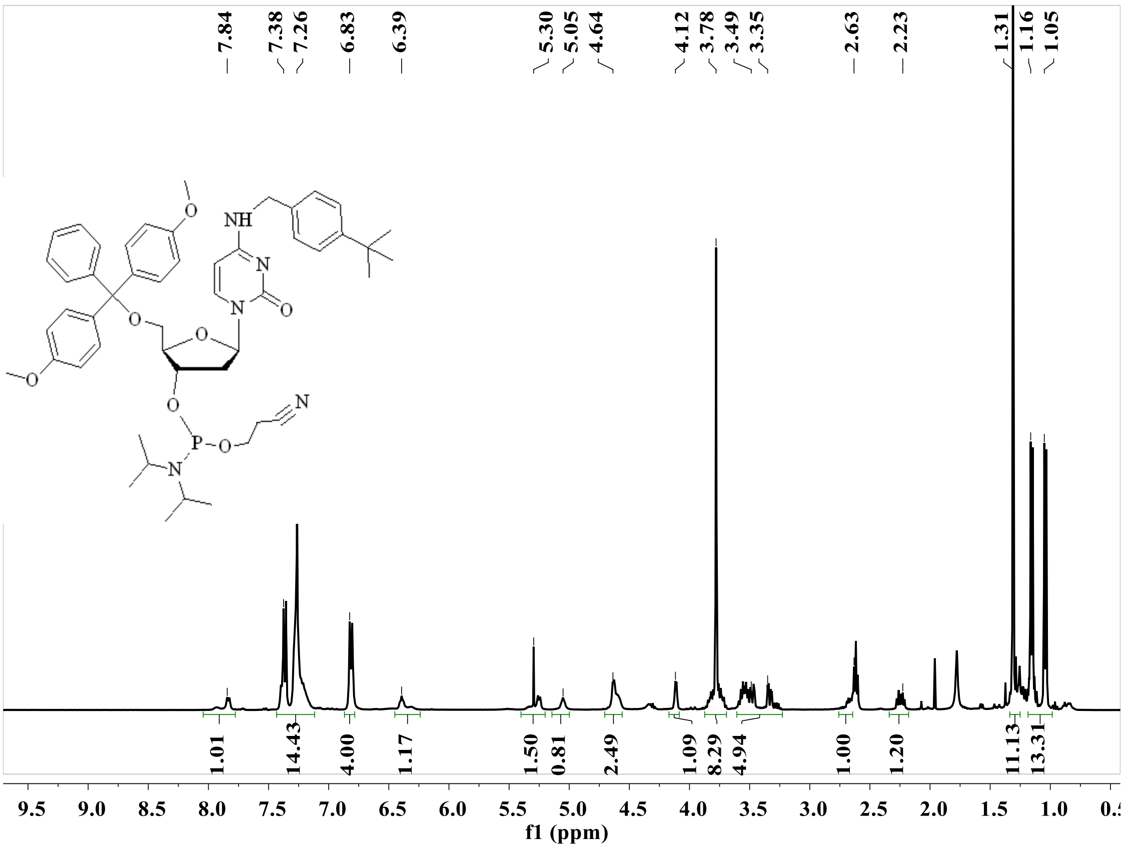

Supplementary Fig. 30. ^{1}H NMR (400 MHz, $CDCl_3$) of compound A6[TB].

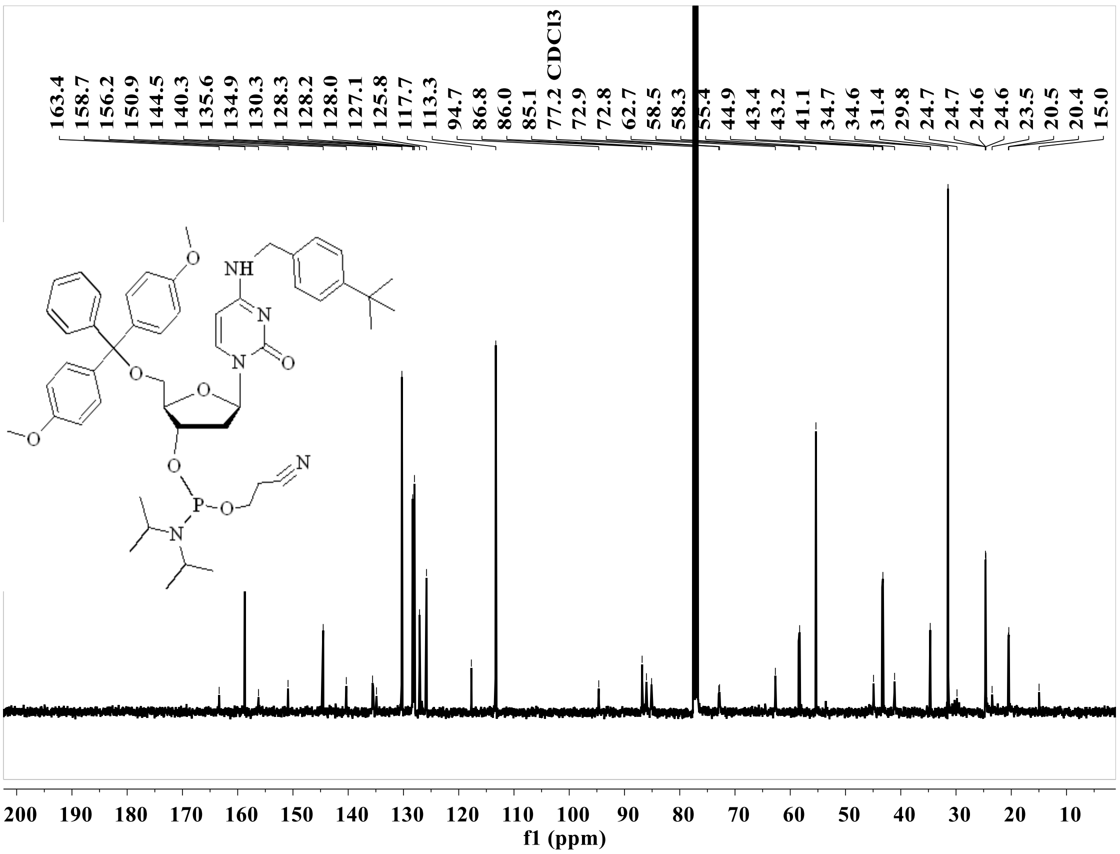

Supplementary Fig. 31. ^{13}C NMR (400 MHz, $CDCl_3$) of compound A6[TB].

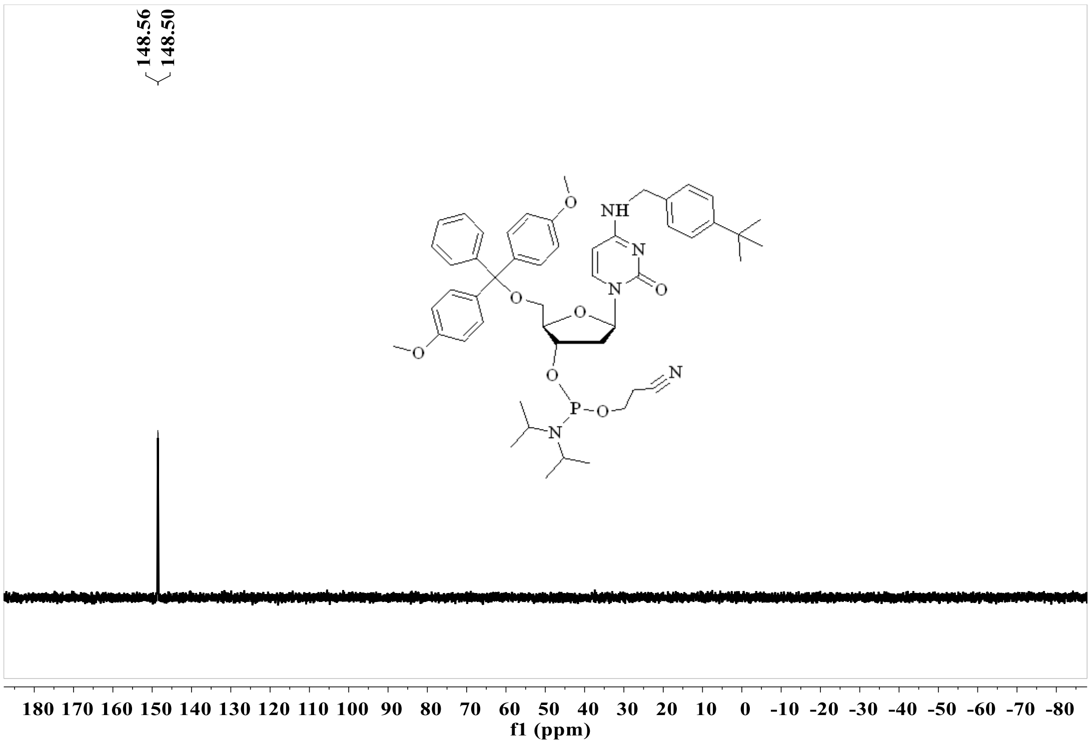

Supplementary Fig. 32. ^{31}P NMR (400 MHz, $CDCl_3$) of compound A6[TB].

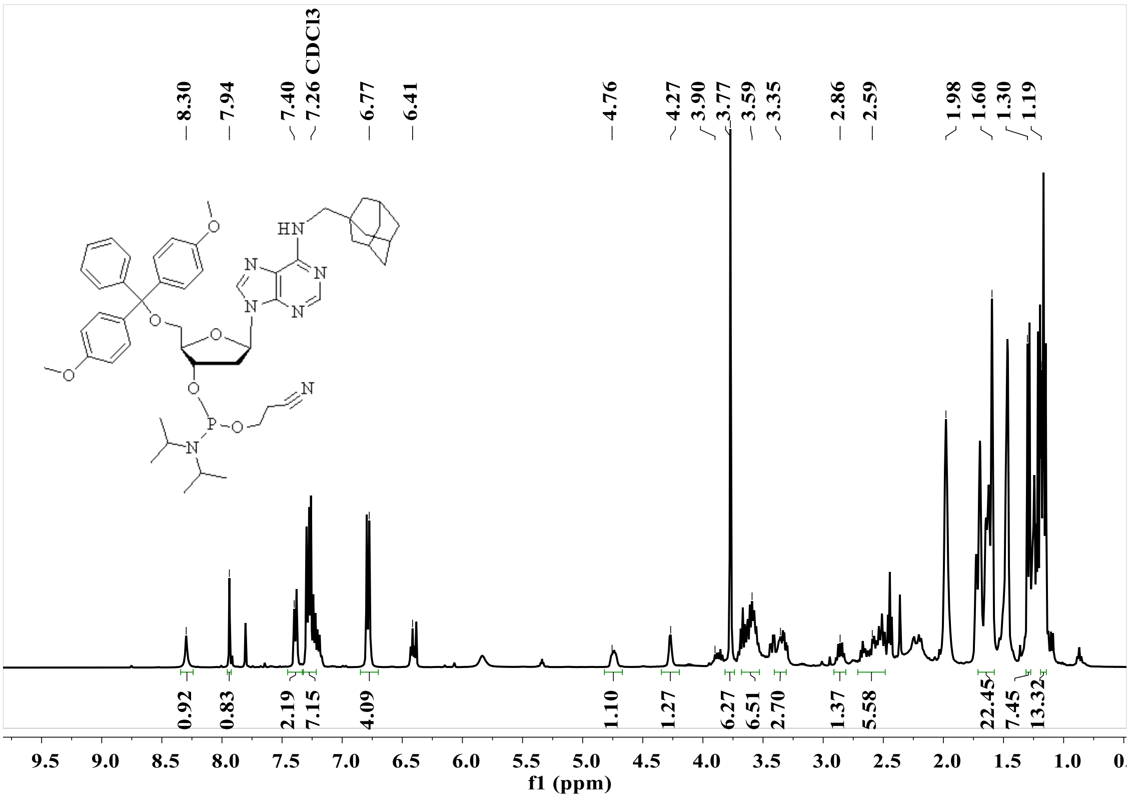

Supplementary Fig. 33. ^{1}H NMR (400 MHz, $CDCl_3$) of compound B4^{AD}.

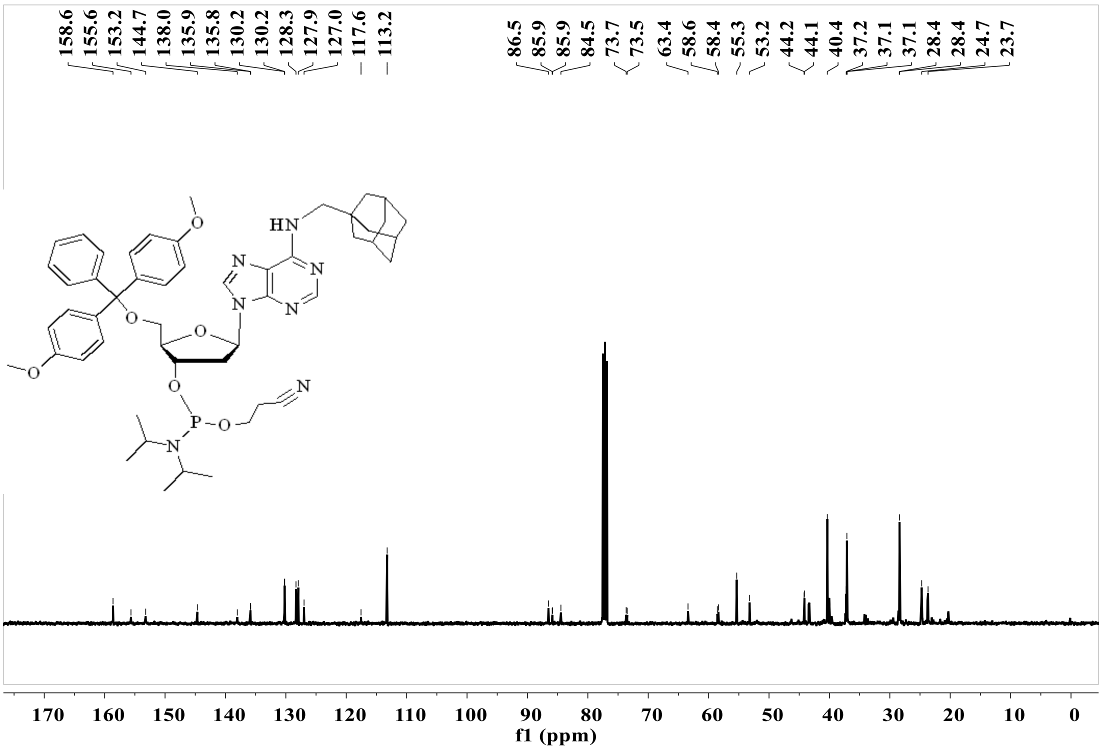

Supplementary Fig. 34. ^{13}C NMR (400 MHz, $CDCl_3$) of compound B4^{AD}.

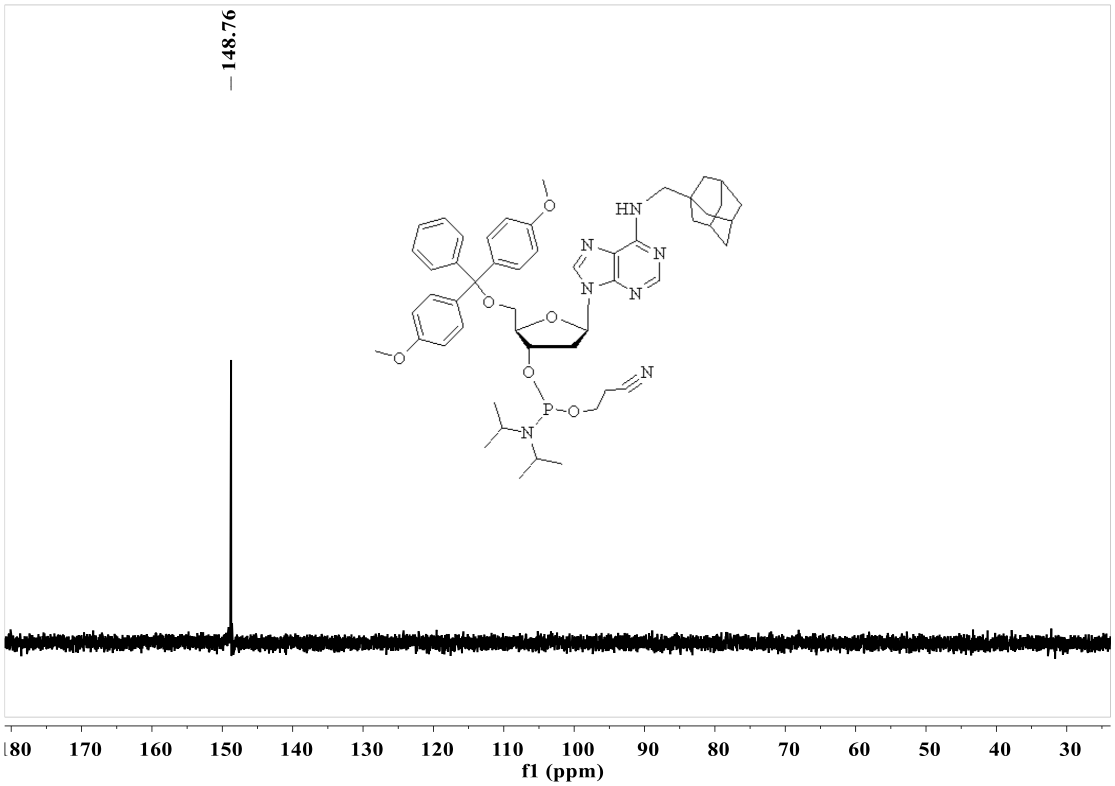

Supplementary Fig. 35. ^{31}P NMR (400 MHz, $CDCl_3$) of compound B4^{AD}.

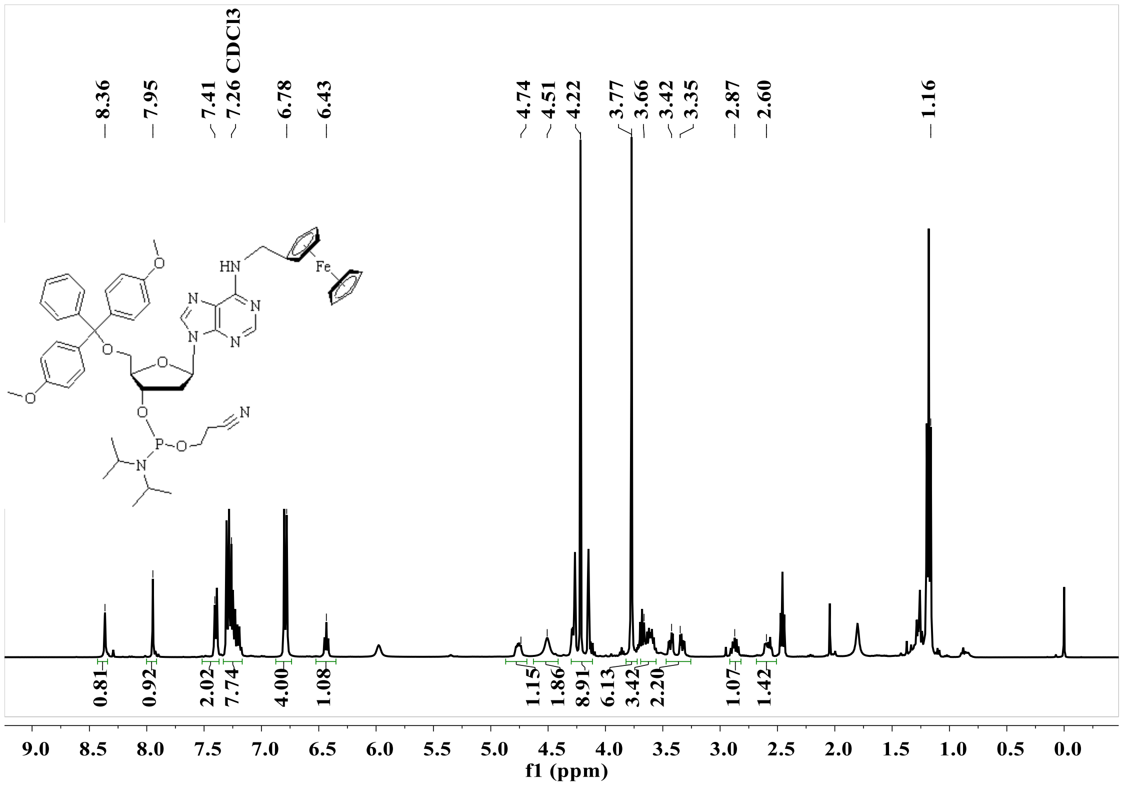

Supplementary Fig. 36. ^{1}H NMR (400 MHz, $CDCl_3$) of compound B4^{FC}.

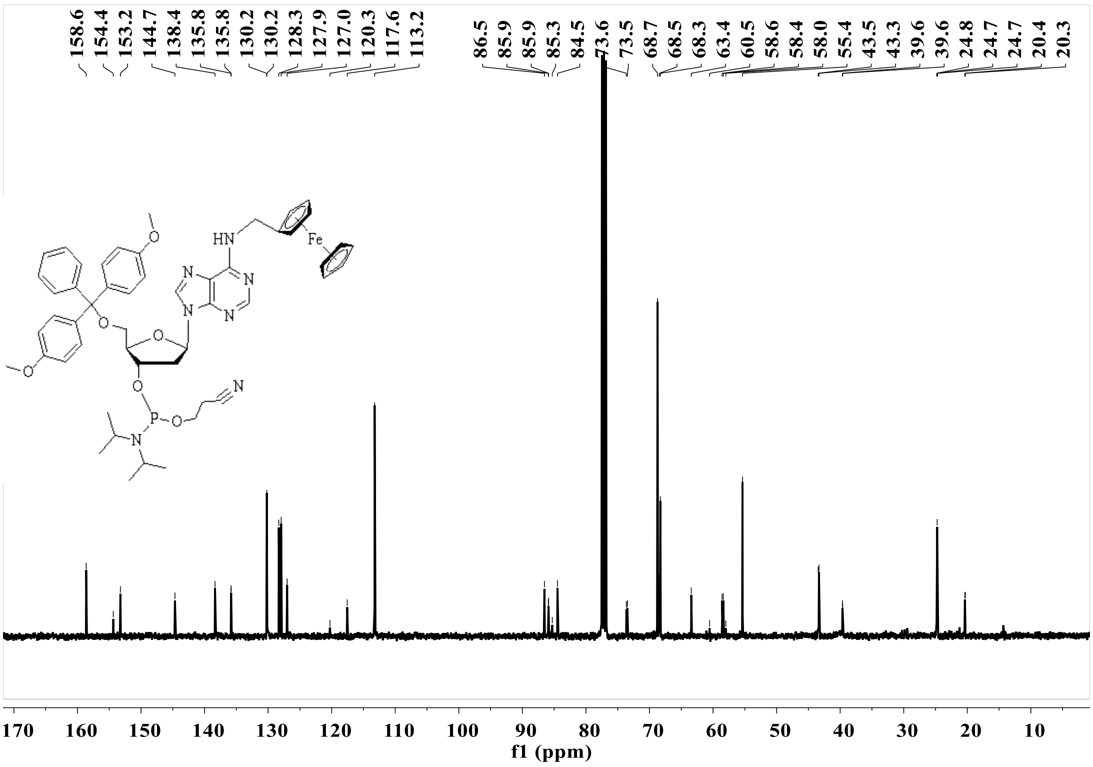

Supplementary Fig. 37. ^{13}C NMR (400 MHz, $CDCl_3$) of compound $B4^{FC}$.

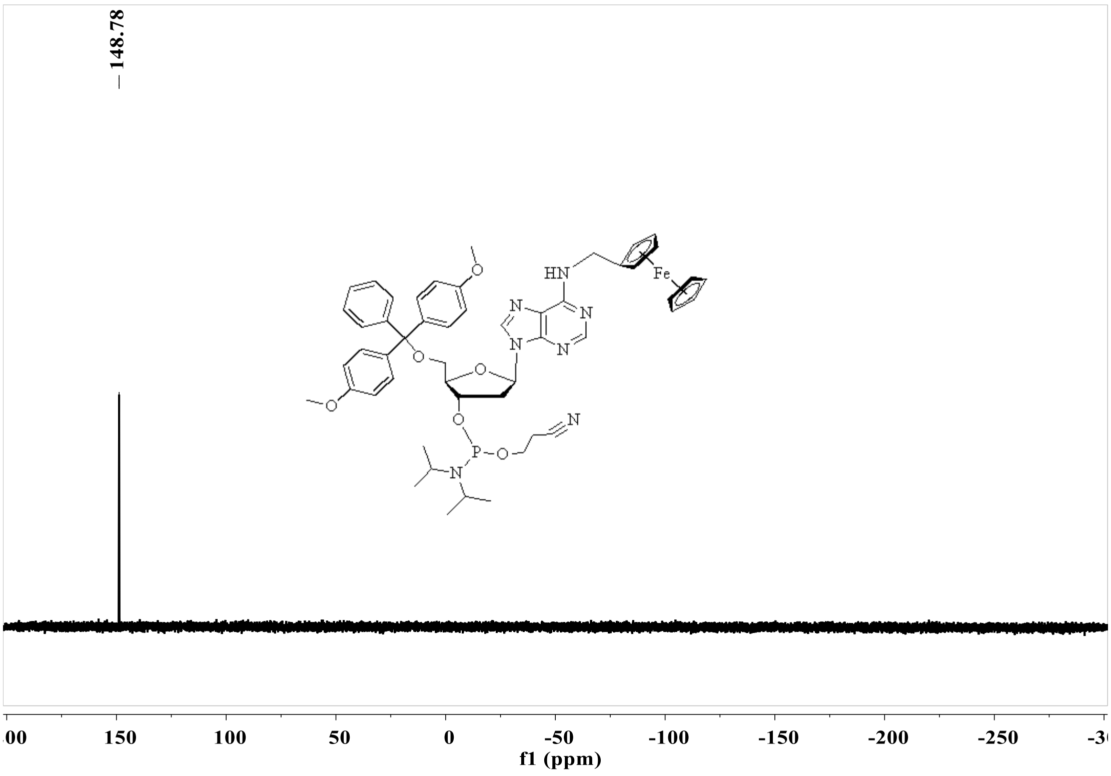

Supplementary Fig. 38. ^{31}P NMR (400 MHz, $CDCl_3$) of compound $B4^{FC}$.

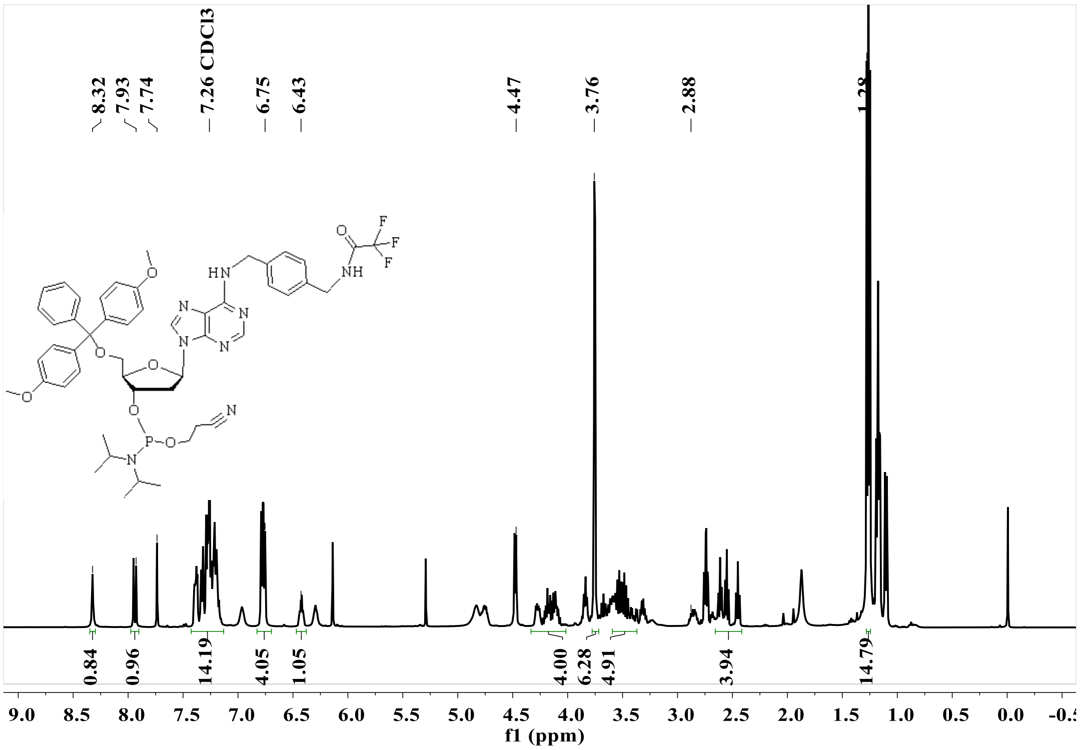

Supplementary Fig. 39. ^{1}H NMR (400 MHz, $CDCl_3$) of compound $B4^{BA}$.

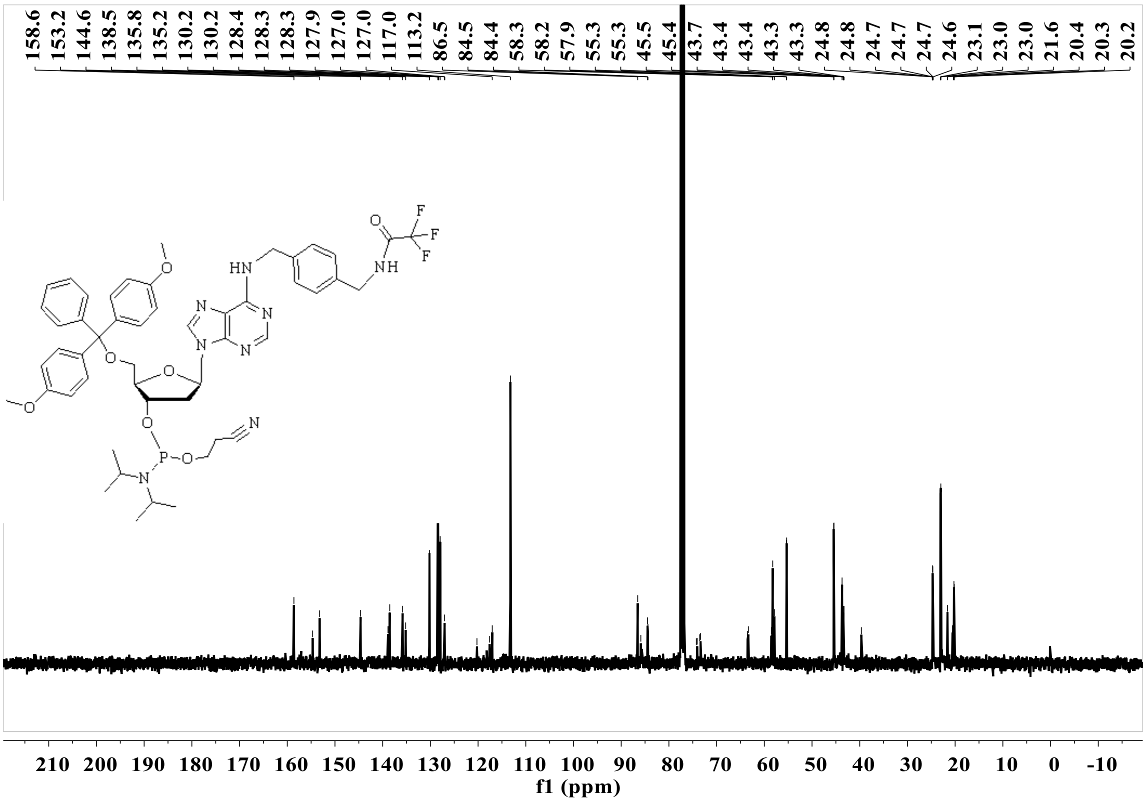

Supplementary Fig. 40. ^{13}C NMR (400 MHz, $CDCl_3$) of compound $B4^{BA}$.

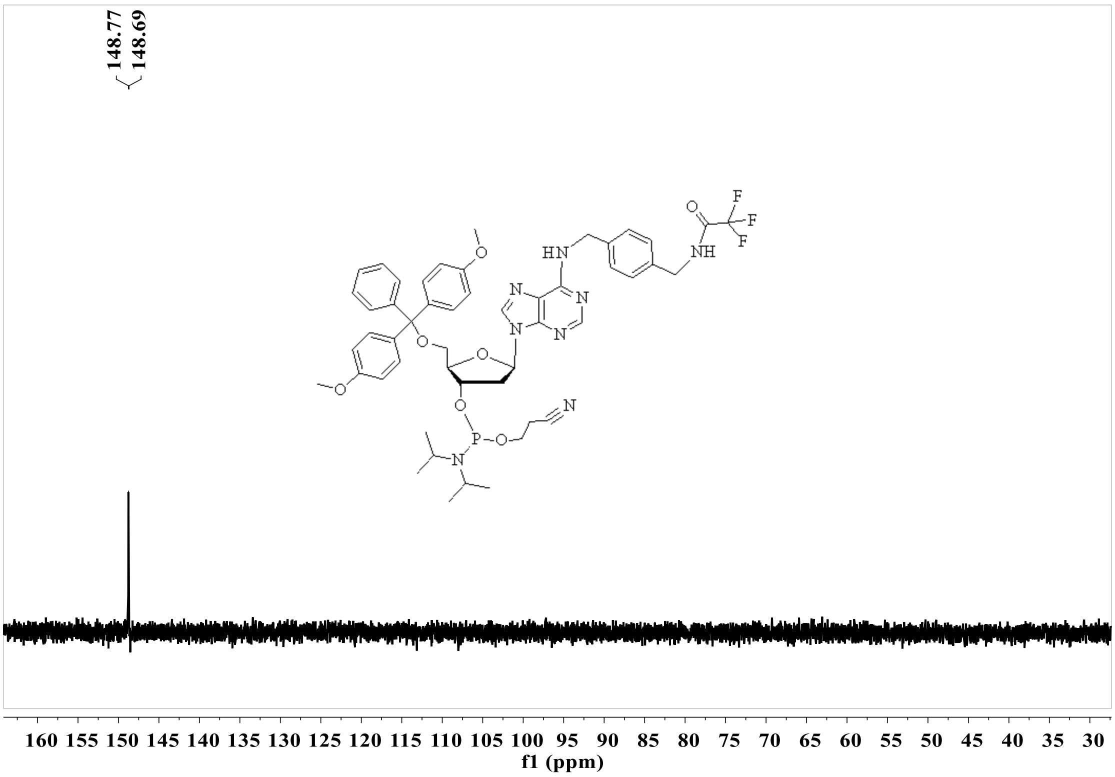

Supplementary Fig. 41.^{31}P NMR (400 MHz, $CDCl_3$) of compound B4^{BA}.

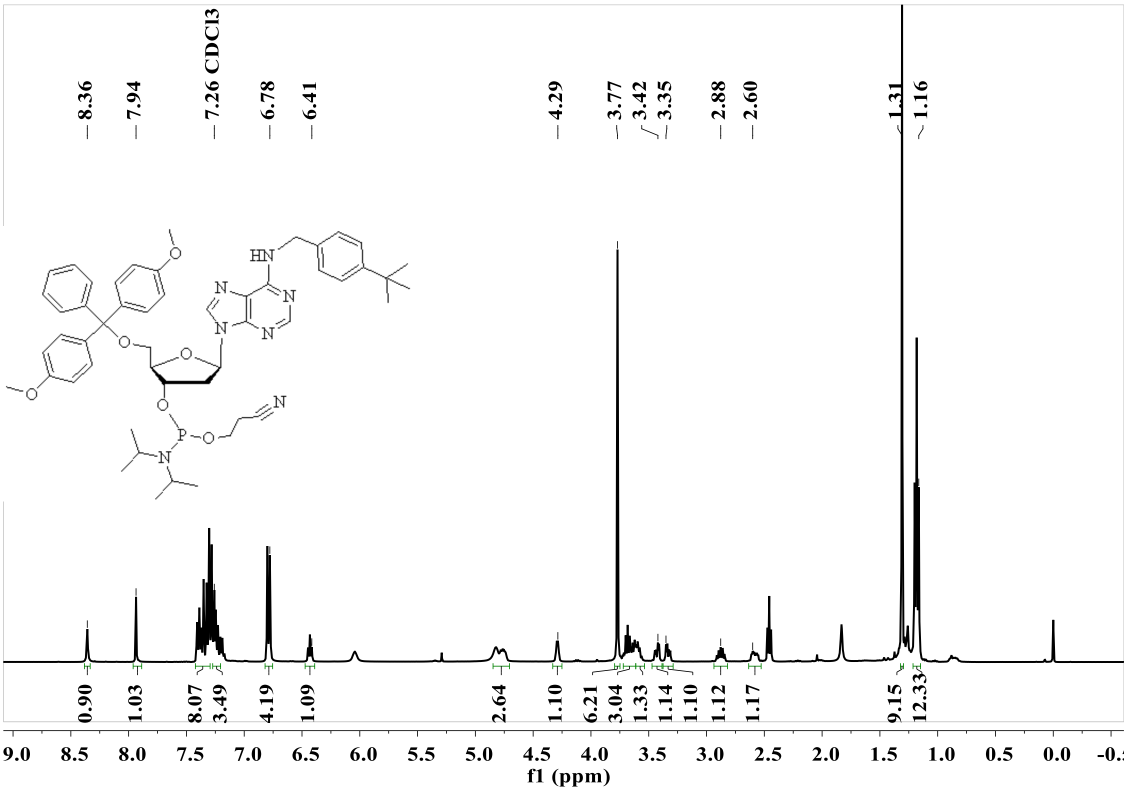

Supplementary Fig. 42. ^{1}H NMR (400 MHz, $CDCl_3$) of compound B4^{TB}.

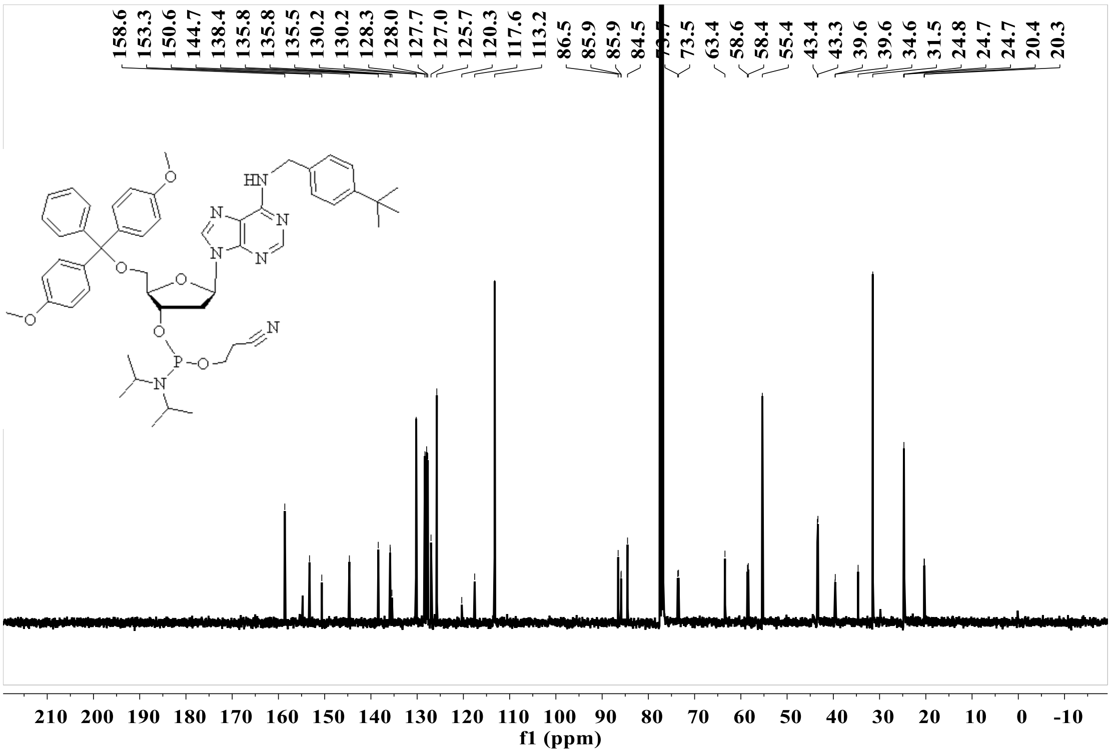

Supplementary Fig. 43. ^{13}C NMR (400 MHz, $CDCl_3$) of compound B4^{TB}.

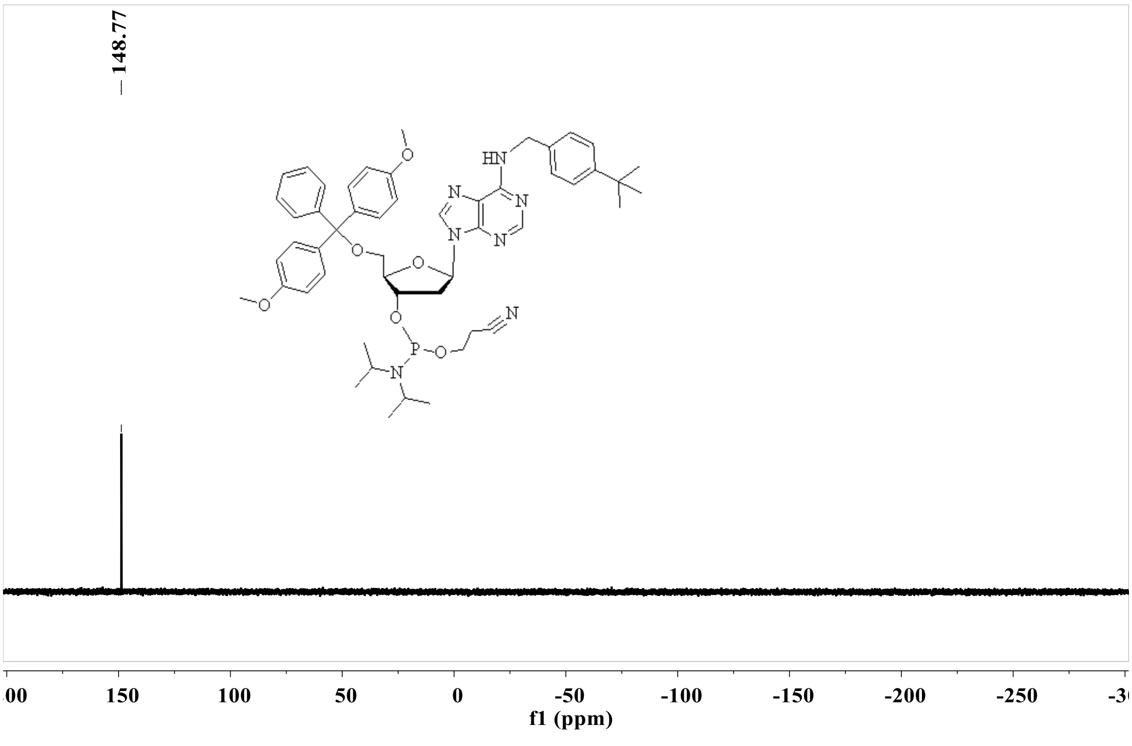

Supplementary Fig. 44. ^{31}P NMR (400 MHz, $CDCl_3$) of compound B4^{TB}.

Supplementary Figures (ESI spectra of guest-containing ODNs)

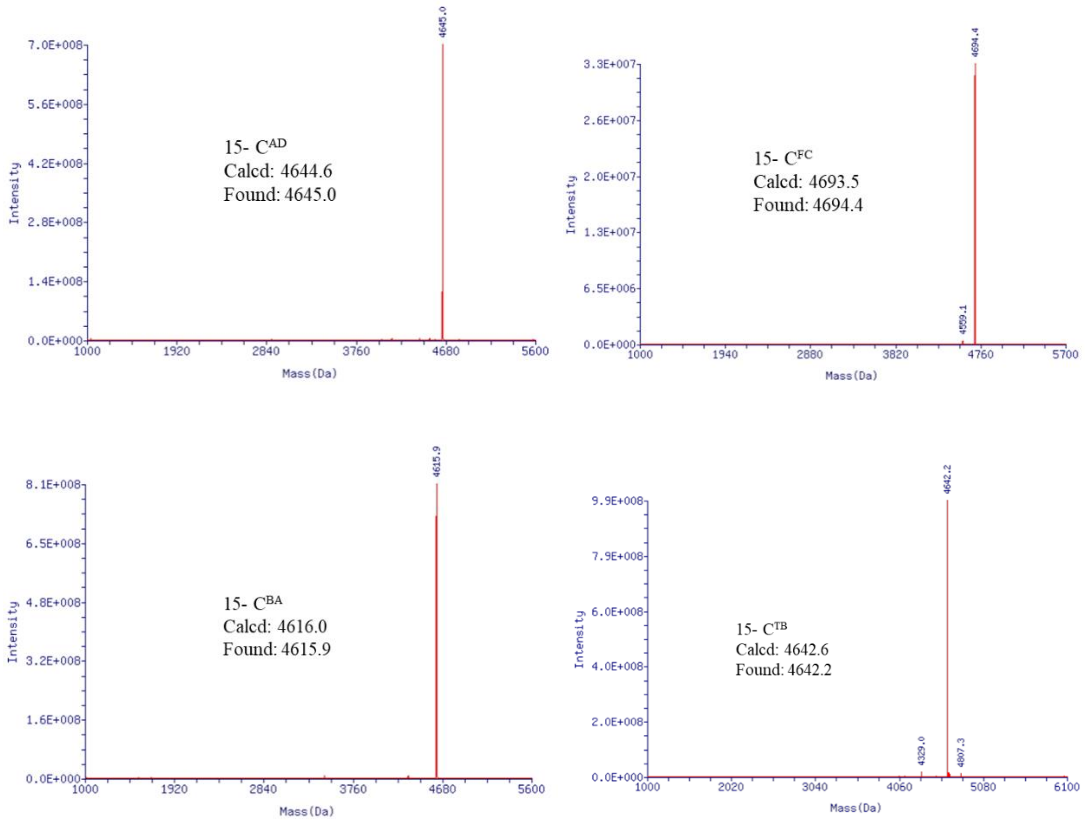

Supplementary Fig. 45. Mass spectra of the cytosine-modified 15 nt ODNs for ITC analysis.

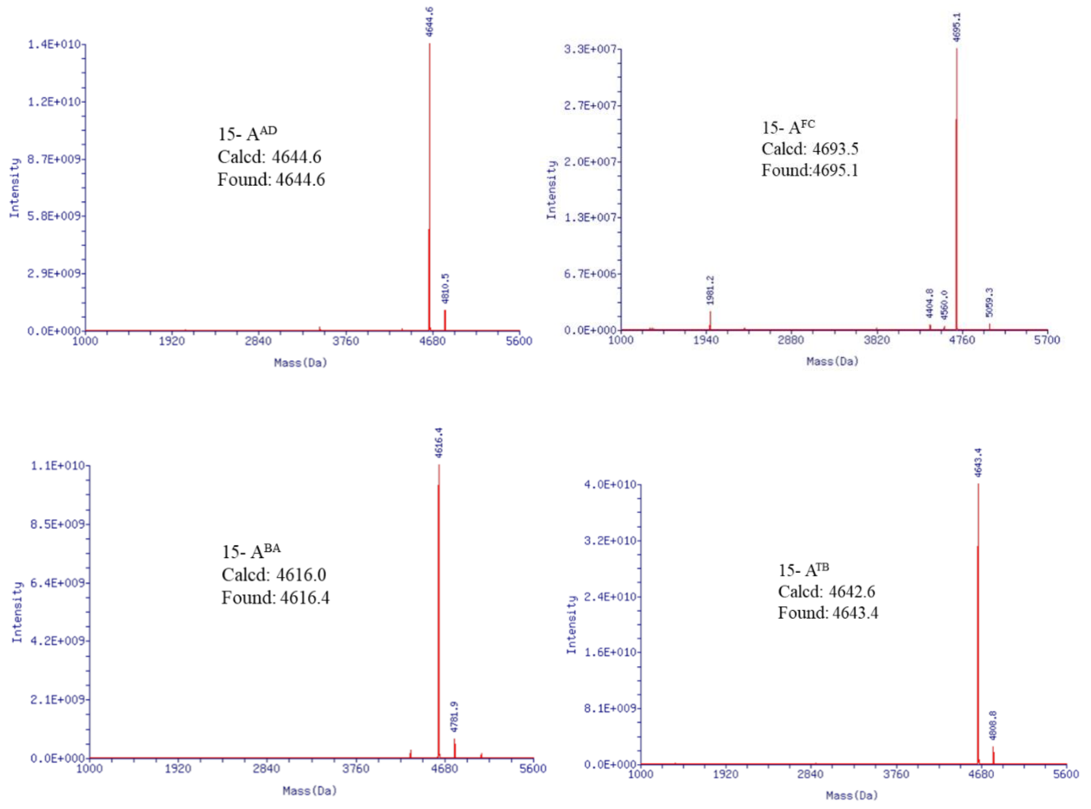

Supplementary Fig. 46. Mass spectra of the adenine-modified 15 nt ODNs for ITC analysis.

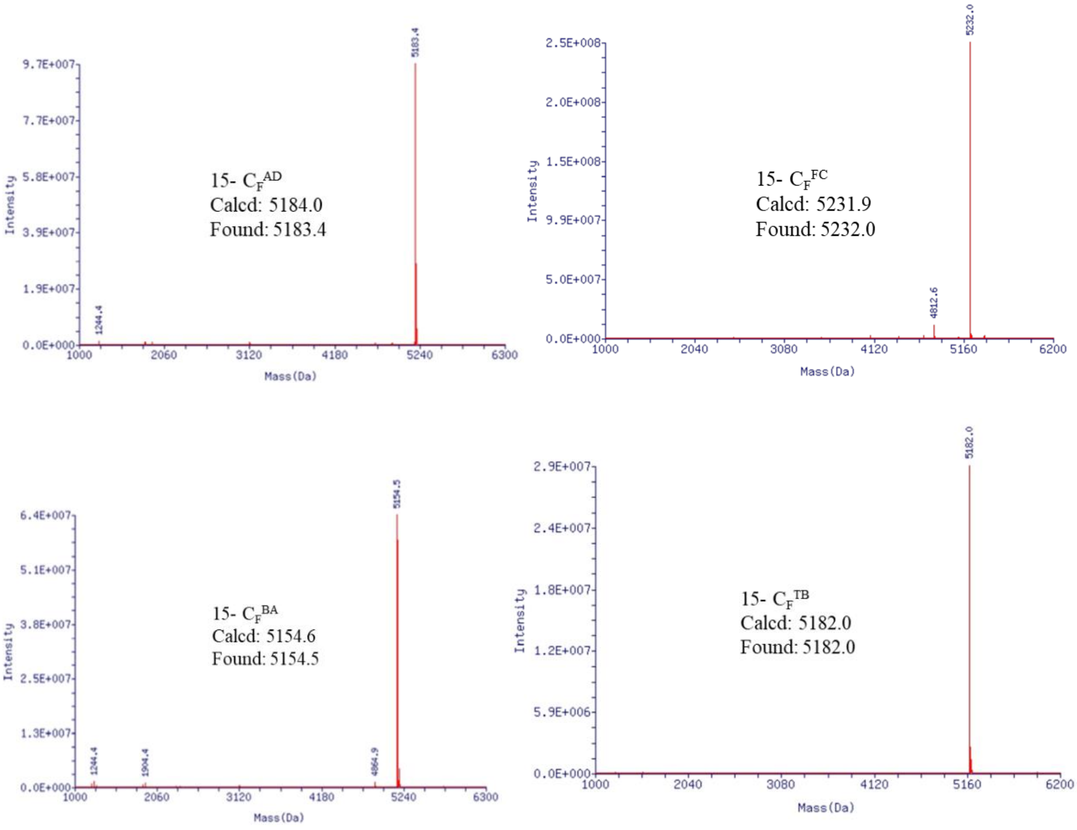

Supplementary Fig. 47. Mass spectra of the cytosine-modified 15 nt ODNs for melting curves analysis.

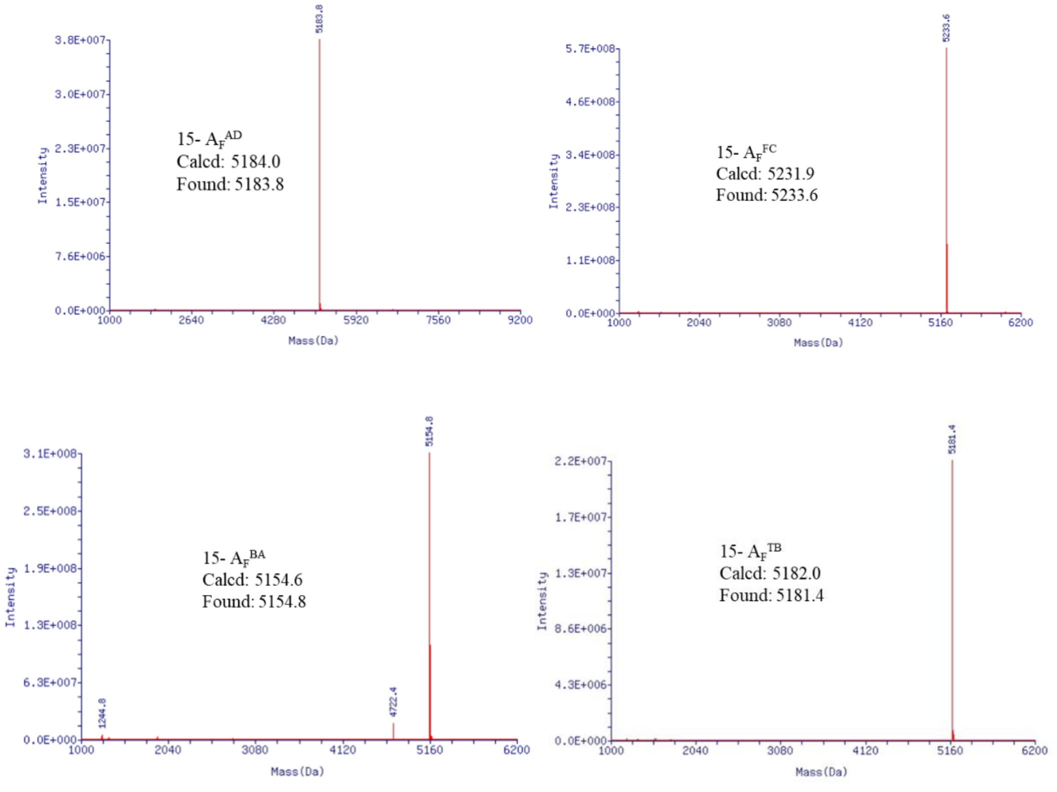

Supplementary Fig. 48. Mass spectra of the adenine-modified 15 nt ODNs for melting curves analysis.

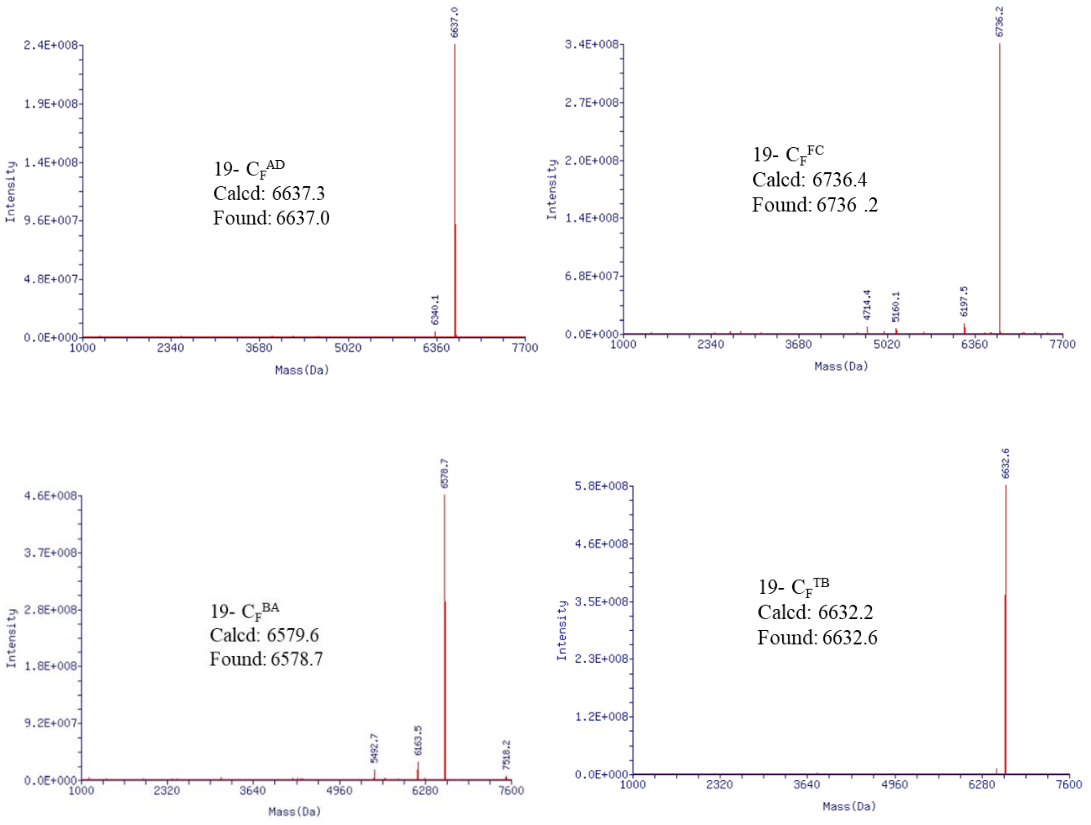

Supplementary Fig. 49. Mass spectra of the cytosine-modified 19 nt ODNs for melting curves analysis.

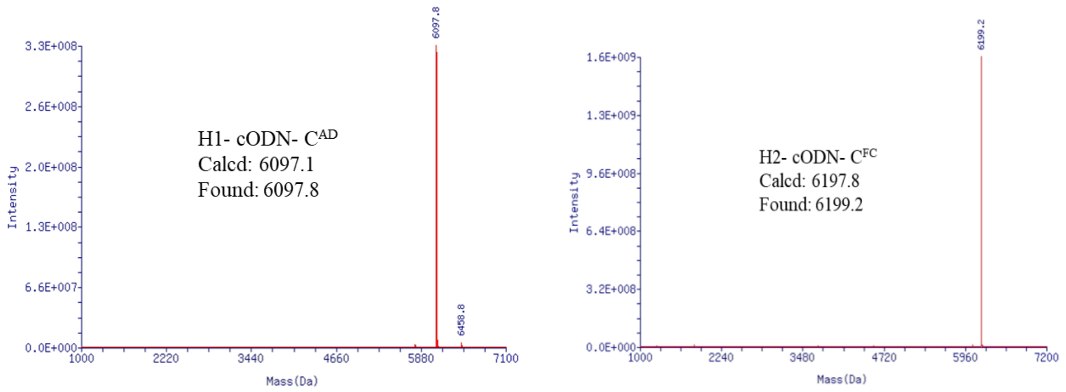

Supplementary Fig. 50. Mass spectra of the cytosine-modified 19 nt ODNs for the hairpin analysis.

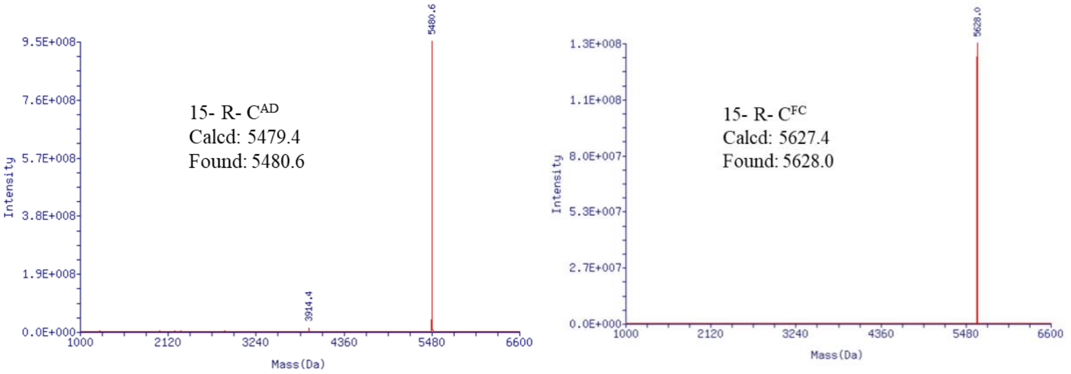

Supplementary Fig. 51. Mass spectra of the adenine-modified 19 nt ODNs for reversible manipulation of DNA duplex.

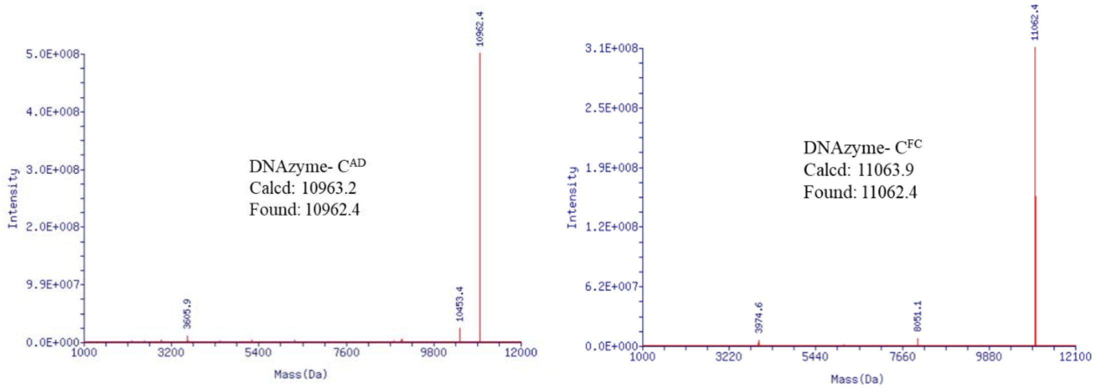

Supplementary Fig. 52. Mass spectra of the adenine-modified 19 nt ODNs for DNAzyme analysis.

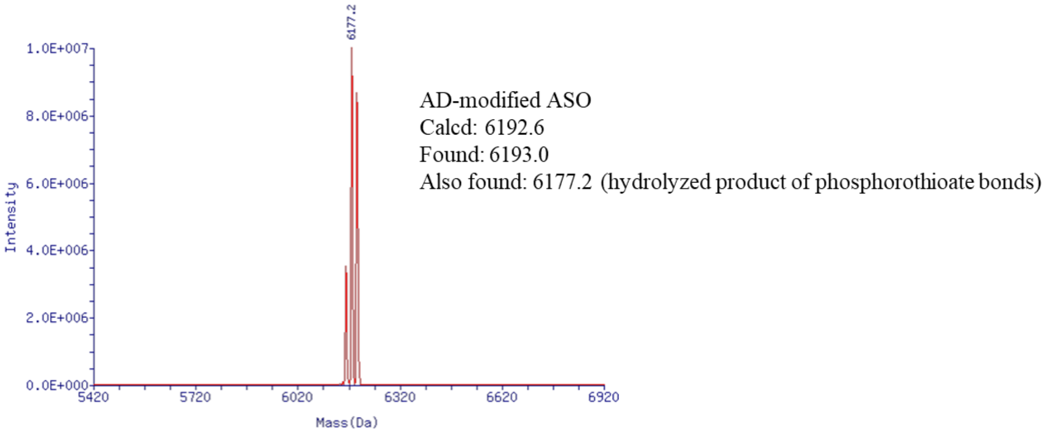

Supplementary Fig. 53. Mass spectra of the adenine-modified 19 nt ODN for the ASO analysis.

Supplementary Figures (Uncropped gel images)

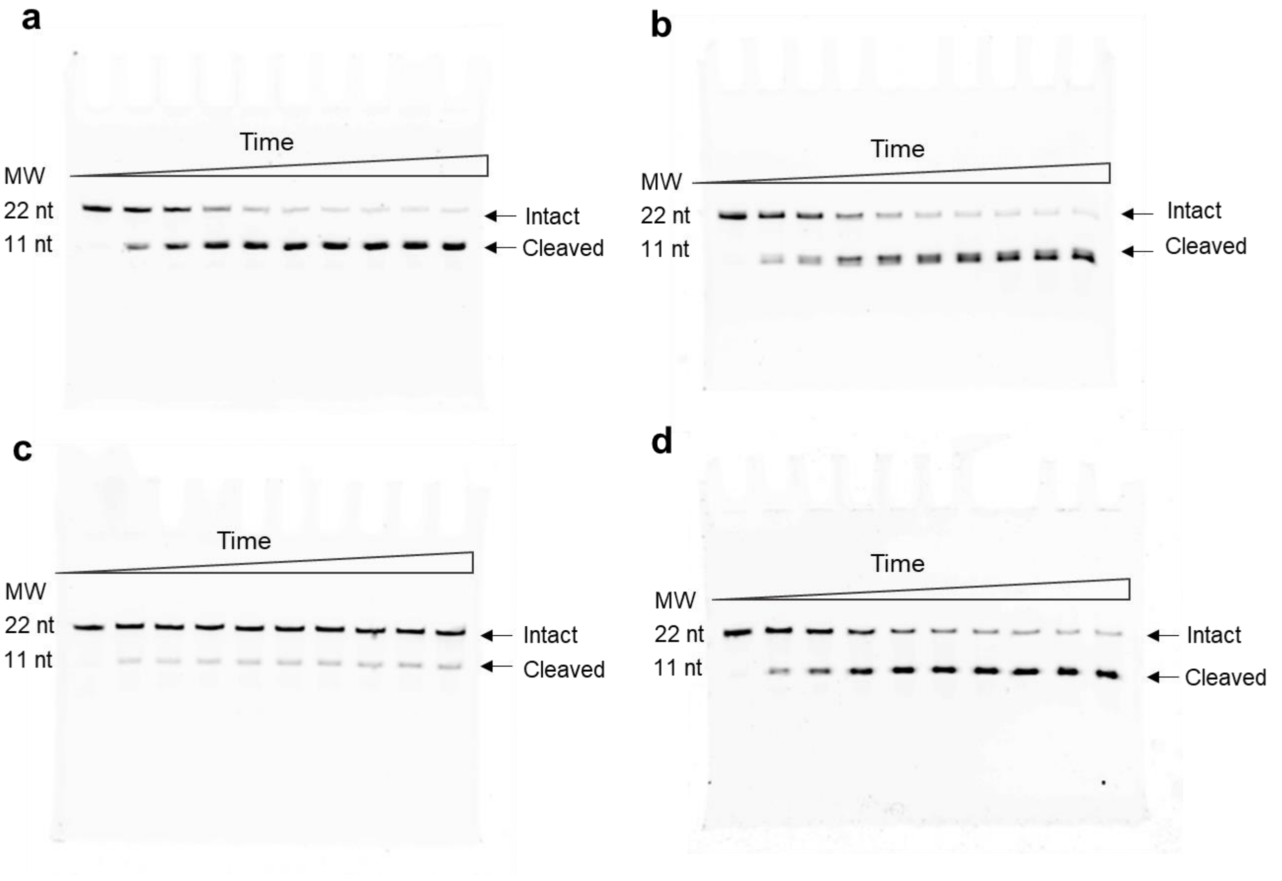

Supplementary Fig. 54. Uncropped gel images for Supplementary Fig. 16. (**a**) Unmodified DNAzyme. (**b**) Fc-containing DNAzyme. (**c**) Fc-containing DNAzyme + CB[7]. (**d**) Fc-containing DNAzyme + CB[7] and Guest.

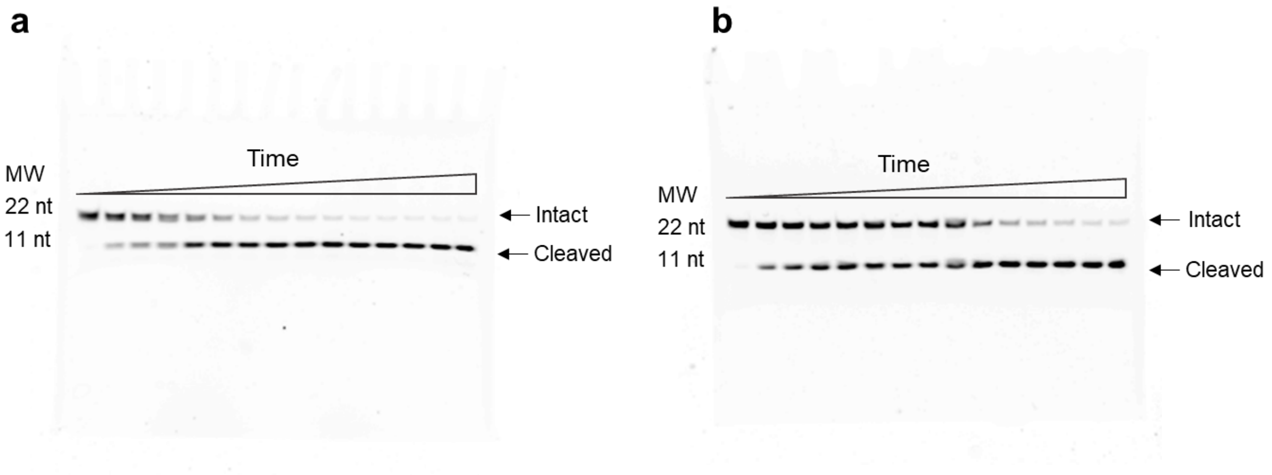

Supplementary Fig. 55. Uncropped gel images for Supplementary Fig. 17. (**a**) Control. (**b**) Manipulated.

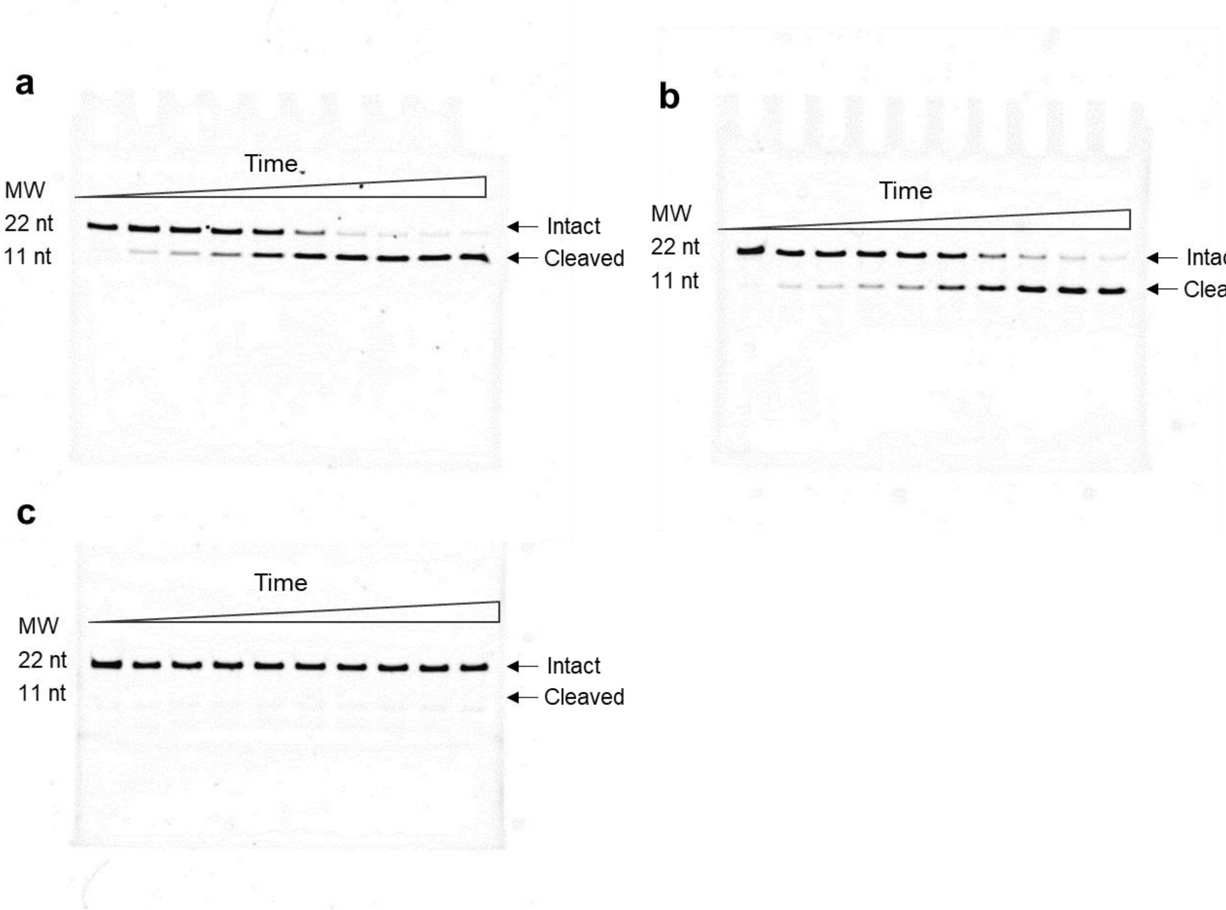

Supplementary Fig. 56. Uncropped gel images for Supplementary Fig. 18. (**a**) Ad-containing DNAzyme. (**b**) Ad-containing DNAzyme + CB[7] and Guest. (**c**) Ad-containing DNAzyme + CB[7].

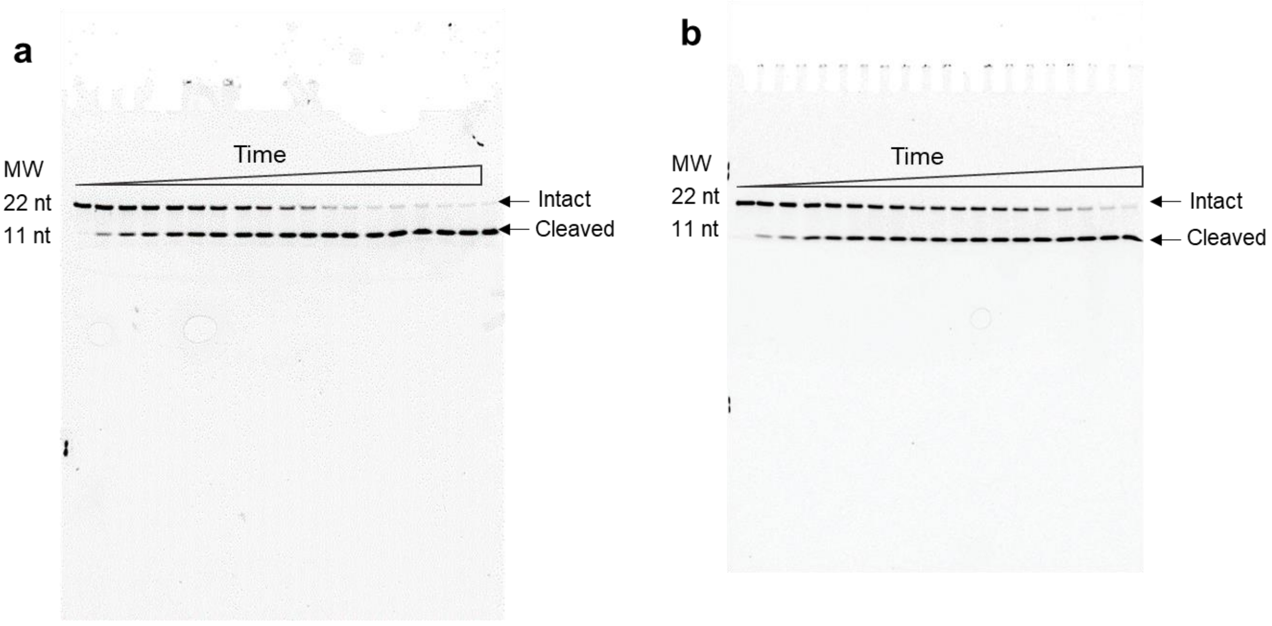

Supplementary Fig. 57. Uncropped gel images for Supplementary Fig. 19. (**a**) Control. (**b**) Manipulated.

Supplementary References

1 Bae, S., & Lakshman, M. K. . O 6-(Benzotriazol-1-yl) inosine derivatives: easily synthesized, reactive nucleosides. *Journal of the American Chemical Society* **129**, 782-789. (2007).

2 Seela, F., & Kaiser, K. Phosphoramidites of base-modified 2′-deoxyinosine isosteres and solid-phase synthesis of d (GCI* CGC) oligomers containing an ambiguous base. *Nucleic acids research* **14**, 1825-1844. (1986).

3 Kierzek, E. & Kierzek, R. The thermodynamic stability of RNA duplexes and hairpins containing N6-alkyladenosines and 2-methylthio-N6-alkyladenosines. *Nucleic Acids Res* **31**, 4472-4480 (2003).

4 Roost, C. *et al.* Structure and thermodynamics of N6-methyladenosine in RNA: a spring-loaded base modification. *J Am Chem Soc* **137**, 2107-2115 (2015).
